# Membrane lipids drive formation of KRAS4b-RAF1 RBDCRD nanoclusters on the membrane

**DOI:** 10.1038/s42003-024-05916-0

**Published:** 2024-02-28

**Authors:** Rebika Shrestha, Timothy S. Carpenter, Que N. Van, Constance Agamasu, Marco Tonelli, Fikret Aydin, De Chen, Gulcin Gulten, James N. Glosli, Cesar A. López, Tomas Oppelstrup, Chris Neale, Sandrasegaram Gnanakaran, William K. Gillette, Helgi I. Ingólfsson, Felice C. Lightstone, Andrew G. Stephen, Frederick H. Streitz, Dwight V. Nissley, Thomas J. Turbyville

**Affiliations:** 1 RAS Initiative, The Cancer Research Technology Program, Frederick National Laboratory, Frederick, MD 21701 USA; 2 Physical and Life Sciences (PLS) Directorate, Lawrence Livermore National Laboratory, Livermore, CA 94550 USA; 3 National Magnetic Resonance Facility at Madison, Biochemistry Department, University of Wisconsin-Madison, Madison, WI 53706 USA; 4 Theoretical Biology and Biophysics Group, Los Alamos National Laboratory, Los Alamos, NM 87545 USA

**Keywords:** Membrane biophysics, Machine learning

## Abstract

The oncogene RAS, extensively studied for decades, presents persistent gaps in understanding, hindering the development of effective therapeutic strategies due to a lack of precise details on how RAS initiates MAPK signaling with RAF effector proteins at the plasma membrane. Recent advances in X-ray crystallography, cryo-EM, and super-resolution fluorescence microscopy offer structural and spatial insights, yet the molecular mechanisms involving protein-protein and protein-lipid interactions in RAS-mediated signaling require further characterization. This study utilizes single-molecule experimental techniques, nuclear magnetic resonance spectroscopy, and the computational Machine-Learned Modeling Infrastructure (MuMMI) to examine KRAS4b and RAF1 on a biologically relevant lipid bilayer. MuMMI captures long-timescale events while preserving detailed atomic descriptions, providing testable models for experimental validation. Both in vitro and computational studies reveal that RBDCRD binding alters KRAS lateral diffusion on the lipid bilayer, increasing cluster size and decreasing diffusion. RAS and membrane binding cause hydrophobic residues in the CRD region to penetrate the bilayer, stabilizing complexes through β-strand elongation. These cooperative interactions among lipids, KRAS4b, and RAF1 are proposed as essential for forming nanoclusters, potentially a critical step in MAP kinase signal activation.

## Abstract (web summary)

RAS binding to RAF’s RBDCRD domain on lipid bilayers cooperatively induces nanoclustering, slowing complex diffusion—an essential mechanism in initial signal activation.

## Introduction

The plasma membrane (PM) has been historically described as an equilibrated two-dimensional fluid composed of lipids and proteins. Numerous biophysical studies now give a more nuanced view of the PM as an active, non-equilibrated system with a complex, heterogeneous and dynamic composition^1^. The active membrane maintains an asymmetrical lipid bilayer composed of thousands of lipid classes, sterols, ion channels, and transmembrane and peripheral proteins that create dynamic nano and meso-scale platforms for sorting proteins and signaling. One such signaling pathway commonly dysregulated in cancer is the MAPK signaling pathway, which is initiated by membrane localized RAS proteins^2^.

RAS proteins are small GTPases that cycle between an inactive “off” state and an active “on” state. In MAPK signaling, membrane-tethered and GTP-bound RAS recruits the effector protein, RAF kinase (ARAF, BRAF and RAF1), in its autoinhibited state to the membrane and promotes conformational changes leading to dimerization of RAF protomers, and activation. The activated homo and heterodimers of RAF bind and phosphorylate MEK, and subsequently ERK. Activated ERK phosphorylates numerous substrates in both the cytoplasm and nucleus leading to cell signaling, cell proliferation and cell growth, and to negative feedback on the MAPK pathway itself. Mutations in RAS, most commonly in codons 12 and 61, lock RAS in a GTP “on” state, constitutively activating the pathway, and leading to enhanced proliferation and dysregulated cell growth^3^. Finding a therapeutic pocket on RAS proteins has been a challenge and only limited number of RAS inhibitors are available to patients in the clinic or in clinical trials. An alternative strategy to limit oncogenic signaling would be to disrupt RAS-mediated activation of RAF on the PM; and therefore, understanding how this protein interacts and assembles on the PM is of fundamental importance.

Recent high-resolution crystal and cryo-EM structures of the recombinant KRAS protein by itself and in complex with RAF have provided new insights into the structural regulation of RAS and RAF proteins^4–7^. However, these studies were performed in the absence of membrane, and therefore, are missing details of the membrane mediated RAS-RAF assembly during the activation cycle. One model is that the formation of RAS nanoclusters on the membrane induce RAF dimerization by bringing two effectors into proximity with each other^8^. Electron microscopy (EM) studies on PM sheets ripped from the apical surface of cells have revealed that RAS proteins form nanoclusters consisting of 7-8 protomers with a diameter of ~18 nm^9^. Other studies have shown these regions to be enriched with certain species of negatively charged lipids, such as phosphatidylserines (PS) and phosphatidylinositols (PIP2)^10,11^. A current hypothesis is that the hypervariable region (HVR) of RAS proteins functions (like a zip code) to localize RAS to regions of the PM enriched with these lipids so that RAS molecules co-localize and form nanoclusters^10^.

Additional evidence for nanocluster formation comes from tracking studies of RAS dynamically diffusing on lipid membranes. Single particle tracking (SPT) experiments of fluorescently tagged RAS have revealed that its diffusion on the PM of live cells is not random and is instead highly heterogenous^12^. For example, KRAS4b shows an isoform-specific, three-state diffusion system comprised of interchanging fast, intermediate, and slow states, while, in contrast, HRAS shows a two-state diffusion system^12,13^. Similarly, in non-stimulated cells, HRAS and KRAS4b molecules exhibit lipid-like diffusion on the PM, while after stimulation with EGF, there is a decrease in the diffusion rate of both HRAS and KRAS4b^14,15^. On artificial membranes composed of two lipids (PC/PS), KRAS4b shows Brownian diffusion; however, on a more complex 8-lipid bilayer, RAS shows cell-like, three-state diffusion, even in the absence of downstream effectors and other components of the signaling complex^16–18^. Altogether, these studies with different membrane systems point to a hierarchical diffusion process regulated by the local lipid composition.

Despite the advancements in imaging technology that facilitate the tracking of individual molecules to nanoscopic levels of spatial resolution and millisecond timescales, the spatial and temporal resolutions of these technologies are unable to resolve the molecular dynamics of proteins and lipids that occur at atomic resolution and nanosecond timescales. It is at these temporal and spatial scales that we expect to identify the precise molecular details that lead to RAS activation of RAF on the plasma membrane. Specifically, it is an open question as to whether active RAS serves as a passive platform for recruitment of RAF to the membrane, or whether RAS and lipids at the membrane participate in regulatory steps, such as nanoclustering, that are necessary for the full activation of RAF. To bridge this gap, we conducted multiscale simulations of RAS and RAF using the Multiscale Machine-Learned Modeling Infrastructure (MuMMI). MuMMI allows us to interrogate RAS and its interactions on the membrane at macro, coarse-grained (CG) and all-atom (AA) spatial and time scales^18,19^ and thus, can capture long-timescale events while preserving detailed atomic descriptions of the relevant ensembles. Most importantly, the simulation studies are guided by experimental results and vice versa, creating an iterative investigation of RAS-RAF biology on the membrane.

In our recent work, we used MuMMI to model the lateral and conformational dynamics of KRAS4b on a model 8-lipid membrane^18^. We reported distinct local lipid micro-environments, called “lipid fingerprints”^20^, corresponding to RAS’s different oligomerization states on the membrane. These lipid fingerprints are also coupled to RAS dynamics and orient RAS into favorable conformations for RAF binding. Here, we extend this work and study how RAF binding to RAS impacts the overall spatiotemporal distribution of the complex on the membrane. We used the RAS binding domain (RBD) and the membrane binding cysteine-rich domain (CRD) domain of RAF1 (amino acid residues 52–192), fully farnesylated and methylated KRAS4b (referred to as KRAS from here onwards), and a supported lipid bilayer composed of eight biologically relevant lipids^21^ to recreate the cellular interaction of KRAS and RAF proteins on the membrane. We employ SPT-TIRF microscopy to measure the lateral diffusion of KRAS and RBDCRD on supported lipid bilayers and paramagnetic relaxation enhancement-nuclear magnetic resonance (PRE-NMR) spectroscopy for the biophysical characterization of RBDCRD interaction with the membrane. The experimental results are complimented with the in silico modeling using the MuMMI framework. Together, we provide a detailed molecular picture of RBDCRD interaction with KRAS on a biologically relevant lipid system. Our findings suggest that the RBD interaction with KRAS and CRD interaction with the lipid membrane cooperatively enhance nanocluster formation.

## Results

### Membrane localization of RBDCRD

**Fig. 1:**
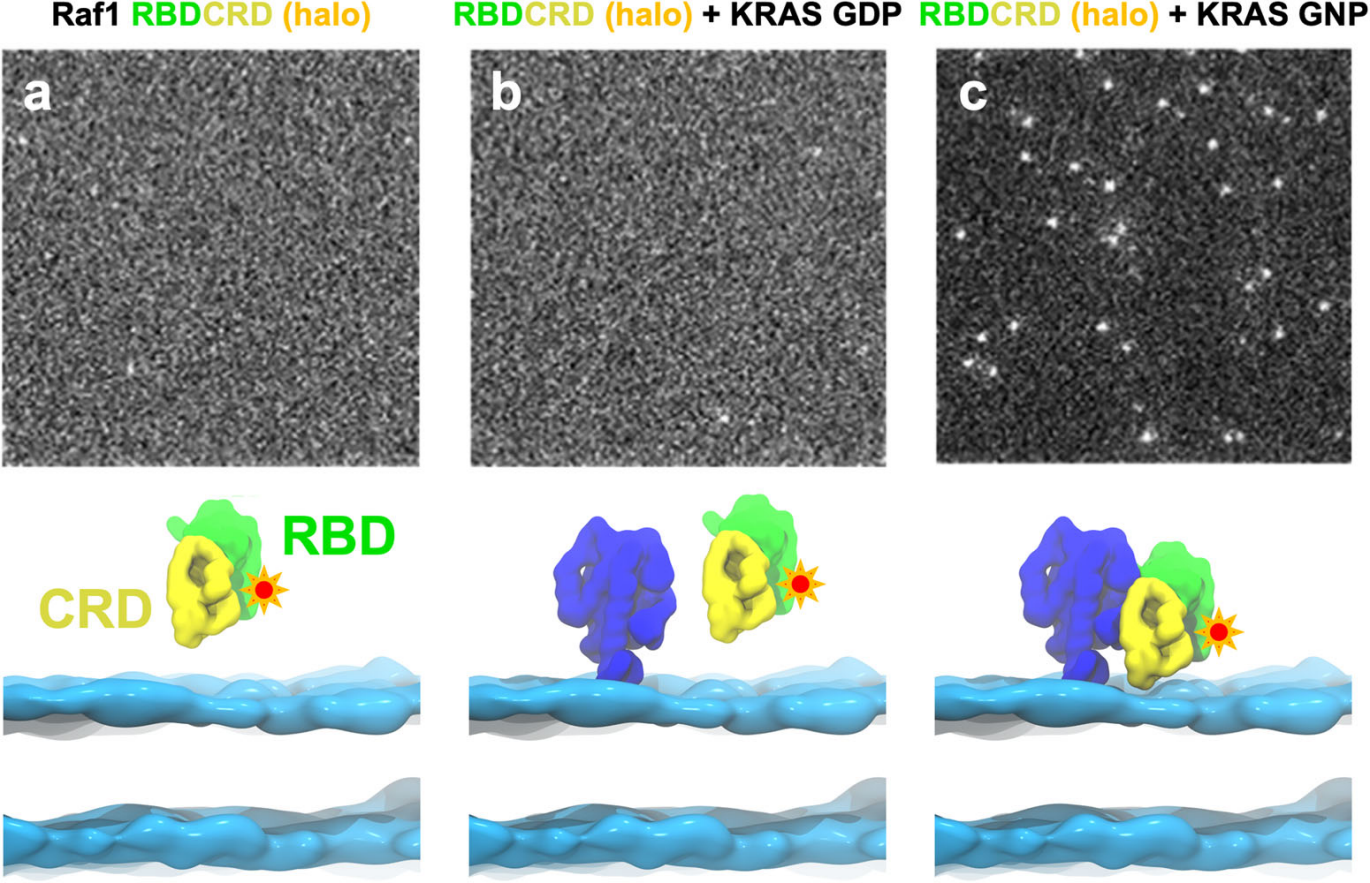
GTP bound KRAS is required for RBDCRD to localize to the supported lipid bilayer. **a** TIRF images (150 pixel X 150 pixel, where 1 pixel is 0.16 µm) of JF646 labeled HaloTag RAF1 RBDCRD deposited onto a supported lipid bilayer composed of eight lipids in the absence of KRAS (**b**) in the presence of KRAS GDP (**c**) in the presence of KRAS GppNHp (non-hydrolysable analog of GTP), abbreviated as GNP in the figure. The bottom panel is the pictorial representation of the proteins and lipid bilayer shown throughout the manuscript. The cyan-blue ribbon-like structure represents the headgroups of the lipid bilayer, the dark blue globular structure represents KRAS, the green represents RBD and the yellow represents CRD.

It is well established biochemically, both in vitro and in cells, that the RBDCRD domain of all RAF proteins binds with high affinity to GTP-bound KRAS^5^. To validate that our experimental system recapitulates the known biology, we first investigated the localization of the RAF1 RBDCRD domain to supported lipid bilayers via TIRF microscopy. TIRF allows the detection of fluorescent molecules that are in proximity (within 100 nm) to the glass/specimen interface. We purified the RBDCRD protein fused to a HaloTag at the C-terminus and then labeled the HaloTag with the fluorescent ligand, JF646^22^. We prepared a supported lipid bilayer composed of eight lipids (POPC/PAPC/DIPE/POPE/PAPS/PIP2/Sphingomyelin/Cholesterol) (see Supplementary Table 1) and added fluorescently labeled RBDCRD alone, or in the presence of KRAS loaded with either GDP or GppNHp, a non-hydrolysable analogue of GTP (referred to as GNP from here onwards). Of the three conditions, the fluorescent particles were enriched in the field of view only in the presence of active, GNP-bound KRAS, as expected (Fig. 1). Therefore, in subsequent experiments we only used the GNP-bound form of KRAS. We also asked whether the individual domains of RBDCRD, i.e., HaloTag RBD and HaloTag CRD, localize to the membrane on their own and observed minimal membrane engagement in the absence of KRAS (see Supplementary Fig. 1). We, therefore, developed an experimental system, validated that it behaves as expected, and that it parallels other experimental systems.

### RBDCRD binding changes the lateral diffusion of the KRAS-RBDCRD complex

It is hypothesized that one role of RAS nanoclustering on the membrane is to facilitate the formation of signaling complexes. Upon binding to RAS on the membrane, autoinhibited RAF undergoes a significant rearrangement that exposes residues that allow RAF to form a hetero or homo dimer via its kinase domain and interact with the membrane via the CRD region. RAF dimerization stabilizes the catalytically active conformation of the enzyme and facilitates the phosphorylation of MEK. However, it is still unclear whether RAS nanoclusters assemble prior to or after RAF binding. We try to deconvolute this process by measuring the lateral diffusion of KRAS protein before and after RBDCRD binding via TIRF-SPT experiments and, based on the trajectories of the molecules, we infer the distribution of cluster formation. Specifically, changes that result from biophysical processes, such as binding or cluster assembly, can be quantitatively measured by analyzing the trajectories. Here, we collected single molecule tracks of membrane-bound, Alexa647 labeled KRAS C118S/S106C before and after addition of unlabeled RBDCRD on a supported 8-lipid bilayer. Our 8-lipid system provides a PM-like lipid environment that is a key regulator of KRAS membrane organization otherwise not attainable in simpler lipid bilayers^17,18^. The use of recombinant proteins, along with photostable dyes, results in longer tracks and the capacity for prolonged monitoring of protein kinetics.

**Fig. 2:**
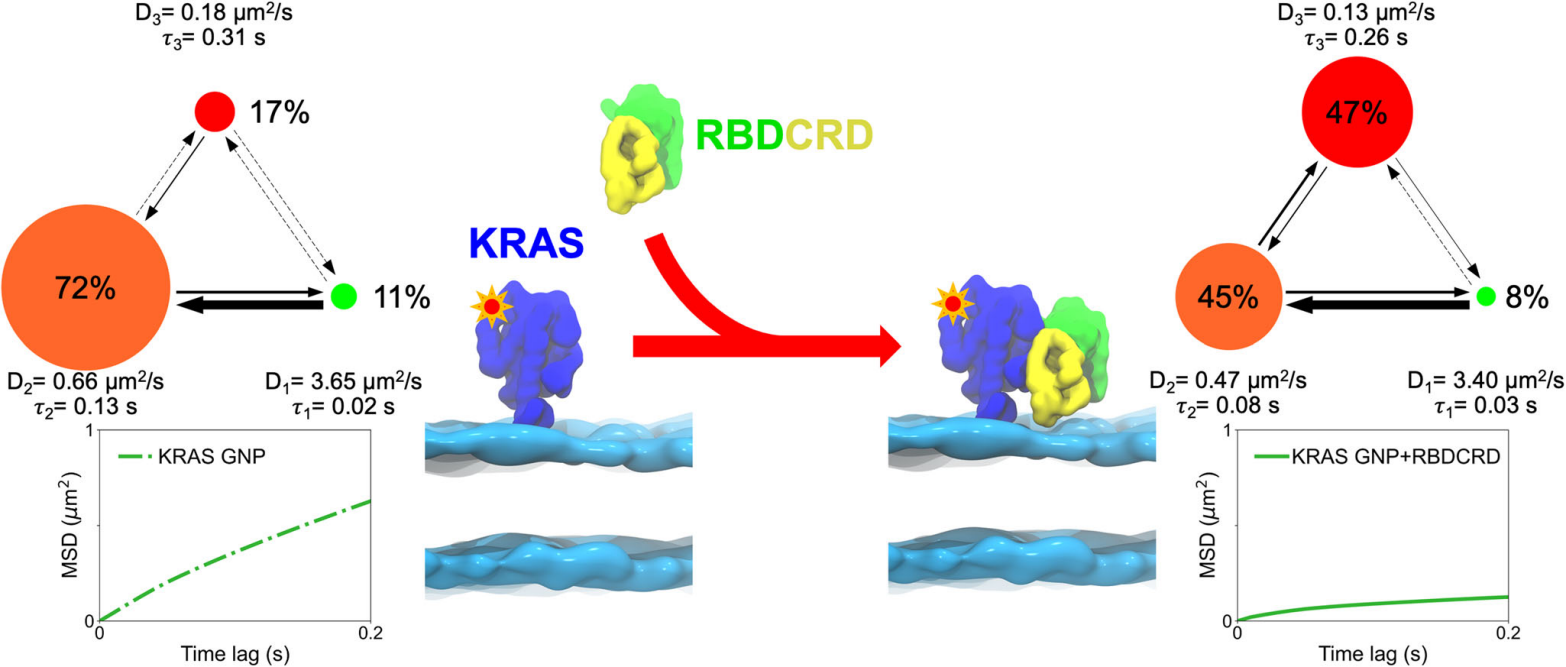
Diffusion analysis of KRAS trajectories on the 8-lipid-supported lipid bilayer in the absence of RBDCRD (left) and in the presence of RBDCRD (right). The top panel shows the three-state diffusion model predicted by Hidden Markov Modeling analysis of the trajectories. The green color represents the fast state, the orange color represents the intermediate state, and the red color represents the slow state. The radius of the circle is drawn proportional to the fractional occupancy in the state, and the width of the arrows is drawn proportional to the transition probability between the two states. The D represents the diffusion coefficient and τ represents the residence time in the state. The bottom panel shows the mean square displacement plots.

The single molecule trajectories were subjected to two types of quantitative analyses: Mean square displacement (MSD) and variational Bayes Single Particle Tracking (vbSPT) analysis of the hidden Markov models (HMM). With respect to MSD analysis, the shape of the MSD plot represents the average diffusion behavior of the ensemble molecules^23,24^. Normal (Brownian) diffusion is depicted by a linear plot, whereas confined diffusion is represented by increasing curvature in the plot (or a bent curve). In the case of normal diffusion, the slope of the curve defines how fast the molecules are moving. KRAS showed confined diffusion on a 8-lipid bilayer as shown by a representative MSD plot in Fig. 2. Upon addition of RBDCRD, the MSD plot of KRAS becomes more confined and exhibits slower diffusion. A careful examination of the individual trajectories reveal that most molecules had shorter diffusive steps with frequent entrapments in the presence of RBDCRD (Supplementary Fig. 2) compared to the longer diffusive steps in the absence of RBDCRD.

To get more insights into these heterogeneous diffusion states, we applied a vbSPT HMM to analyze thousands of trajectories^25^. This approach identifies the underlying diffusion states directly from the experimental data without any prior knowledge and calculates the transition probabilities between those states. In our experiments, the HMM analysis predicted a best-fit, three-state diffusion model for KRAS in both the absence and the presence of RBDCRD, as shown in Fig. 2. The fast (D_1_), intermediate (D_2_) and slow (D_3_) diffusion states are represented by the green, orange and red circles respectively, and the width and the direction of the arrows are drawn in proportion to the transition probability calculated between the states. Similar to our previous observations^17,18^, monomeric KRAS molecules in the fast diffusion state quickly transitioned into the intermediate state (transition probability = 22.5 s^−1^), thus making it the most populated state (72%). The average diffusion constant in the intermediate state is 0.66 µm^2^/s with a lifetime of 0.13 s. Further, KRAS in the intermediate state rarely transitioned into the slow state, which was characterized by a diffusion constant of 0.18 µm^2^/s and a lifetime of 0.31 s.

Intriguingly, upon the addition of the RBDCRD domain, the distribution for the most populated state shifted from the intermediate state to the slow state. Specifically, the fractional occupancy of the slow state increases from 17% to 47% in the presence of RBDCRD. The diffusion constants of all three states decreased slightly, although still within the standard deviation, which, most likely, can be attributed to the increase in mass upon RBDCRD binding. The lifetime of the intermediate state declined and the transition probability from the intermediate to the slow state increased, suggesting a higher transition rate into the slow state. One hypothesis is that upon RBDCRD binding, KRAS-RBDCRD complexes instantaneously assemble into clusters with slow diffusion.

### KRAS-RAF complex diffusion is dependent on individual RAF domains

**Fig. 3:**
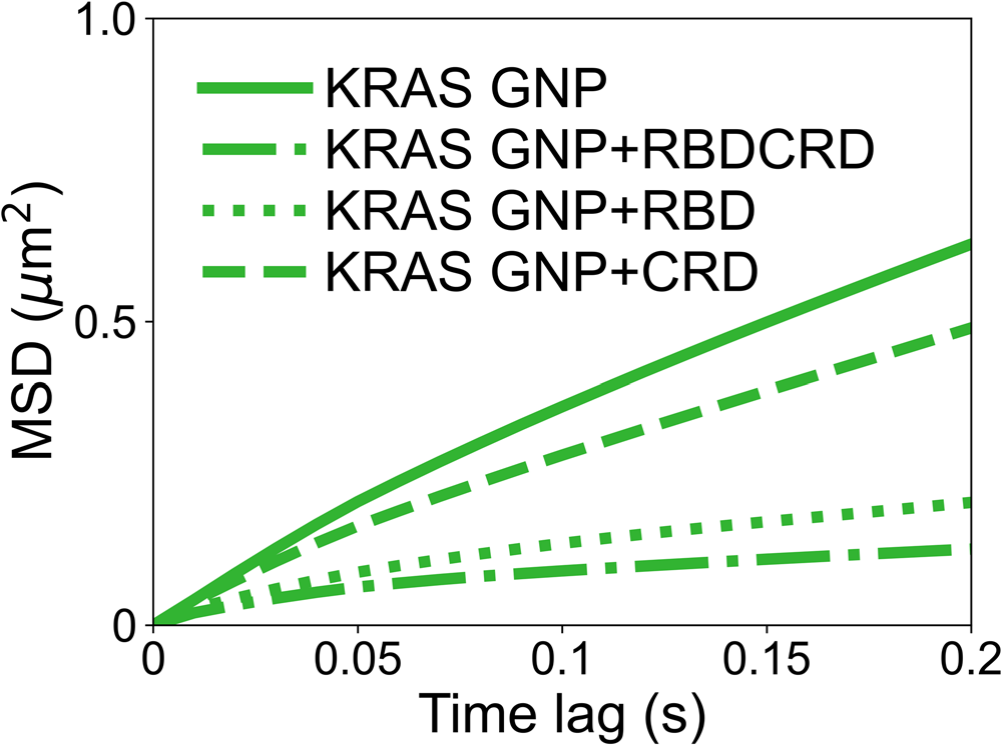
Binding of RBD and RBDCRD to membrane tethered KRAS leads to increased confinement of KRAS. Mean square displacement (MSD) plots of KRAS on its own (long-dashed-dot), in presence of CRD (dash-dash), RBD (dotted) and RBDCRD (solid). The corresponding raw data with associated errors are tabulated in Supplementary Table 3.

We next investigated how the isolated RBD and the isolated CRD domains of RAF1 affect KRAS diffusion on the membrane to dissect the relative contributions of each to the diffusion process. We calculated the mean square displacement (MSD) of KRAS only, KRAS plus RBD, KRAS plus CRD and KRAS plus RBDCRD on an 8-lipid bilayer. As seen in Fig. 3, KRAS shows confined diffusion in all cases, but has a different degree of confinement depending on which of the domains have been added to the system. Increased confinement of KRAS is observed in this ascending order: CRD < RBD < RBDCRD. The greatest degree of confinement is seen when the RBDCRD is added to KRAS. We also see a pronounced increase in confinement with the addition of the RBD domain but very minimal changes with CRD domain.

We interpret these results as follows: The binding affinity of CRD alone to KRAS is weak, yet key residues within the CRD can interact with the membrane, which may hinder the lateral diffusion of KRAS^5,26^. However, the partition coefficient of CRD to the membrane is also weak (Kp ~ 177)^27^ and, hence, in our TIRF experiments (Supplementary Fig. 1), CRD membrane localization is either rare or transitory. This results in a relatively modest change in the MSD plots of KRAS in the absence and the presence of the CRD domain. RBD, on the other hand, binds to the G-domain of KRAS with a binding affinity of 350 nM and is readily recruited to the bilayer, resulting in a more dramatic impact on KRAS diffusion. However, since it has no side chains that directly contact the lipid bilayer, it has less effect on KRAS diffusion relative to RDBCRD, which combines high affinity for active KRAS through the RBD and affinity for lipids via CRD residues (Kp ~ 2815)^27^. Altogether the results suggest that RBD engagement with KRAS G-domain and the CRD interaction with the membrane have the maximum impact on KRAS lateral diffusion on the membrane.

### KRAS-RAF complex diffusion is dependent on lipid composition

To understand the role of lipid composition on RAS-RBDCRD interactions on the supported lipid bilayers, we systematically varied the lipid composition of our artificial membranes. In addition to our standard 8-lipid mixture, we created four other lipid compositions to test the effects of lipid complexity, charge density, and the presence or absence of cholesterol and sphingomyelin. We prepared bilayers as follows: (i) POPC/POPS (80/20) (ii) POPC/PIP2 (95/5) (iii) POPC/PAPC/DIPE/DOPE/PAPS/PIP2 (termed ‘6-lipid’) and (iv) POPC/PAPC/DIPE/DOPE/PAPS/PIP2/SM (termed ‘7-lipid’ bilayers). In all cases, the charge distribution was kept consistent with the charge distribution of the lipids in the 8-lipid composition.

**Fig. 4:**
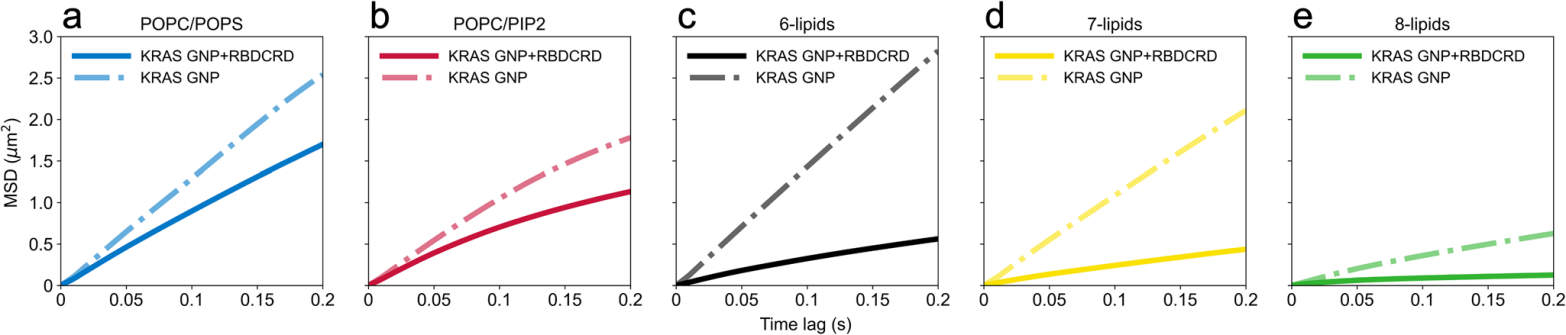
RBDCRD binding to the membrane tethered KRAS increases confinement on various lipid compositions. Mean square displacement (MSD) plots calculated from the trajectories of KRAS diffusing on various types of lipid bilayers collected before and after addition of RBDCRD represented by long-dash-dot lines and solid lines respectively. **a** The blue line represents POPC/POPS (20%), **b** the red line represents POPC/PIP2 (5%), **c** the black line represents the 6-lipid bilayers (POPC/PAPC/PAPE/DIPE/PAPS/PIP2), **d** the yellow line represents the 7-lipid bilayers (POPC/PAPC/PAPE/DIPE/PAPS/PIP2/DPSM), and **e** the green line represents the 8-lipid bilayers (POPC/PAPC/PAPE/DIPE/PAPS/PIP2/DPSM/Cholesterol). The corresponding raw data with associated errors are tabulated in Supplementary Tables 4, 5.

Figure 4a–d shows the ensemble MSD plots of KRAS before (long-dash-dot lines) and after addition of RBDCRD (solid lines) for each of the different lipid compositions, including the 8-lipids in Fig. 4e. The HMM analysis results, including diffusion rates and fractional occupancies, are shown in Supplementary Table 2. KRAS on its own showed lipid-dependent diffusion behavior. For example, on a simple 2-lipid bilayer (POPC/POPS), KRAS showed Brownian diffusion; and, while the diffusion rates were different, KRAS also randomly diffused on cholesterol depleted 6-lipid and 7-lipid bilayers (Supplementary Table 2). In contrast, on the POPC/PIP2 bilayer, KRAS showed confined diffusion compared to the POPC/POPS bilayer, but the diffusion rates were faster than on the 8-lipid bilayer (Supplementary Table 2).

After the addition of RBDCRD, the diffusion slowed down and became more confined on all lipid compositions. However, the extent of confinement was greater for more complex lipid compositions than the 2-lipid compositions. The HMM analysis of the KRAS trajectories revealed that the population of the slow-moving KRAS molecules systematically increased compared to freely-moving monomeric KRAS with the more complex membrane system. The addition of RAF1 domains, RBD only and RBDCRD further increased the population of both the intermediate and slow states in all lipid compositions. However, the greatest incremental increase of the slow state was observed when KRAS bound to the tandem RBDCRD domain resulting in the confined MSD plots. Together the results suggest that RBD binding to the G-domain, the CRD interaction with the membrane, and the complex lipid composition affect how the KRAS/RBDCRD complex diffuses on the membrane.

### RBDCRD binding promotes nanoclustering on the membrane

#### Experimental evidence of nanocluster formation on supported lipid bilayer

In a recent work based on fluorescence correlation spectroscopy (FCS) measurements, Packer et al. showed that RBD binding to KRAS induces dimerization on artificial membranes^28^. Here, the diffusion rate that we measure for KRAS upon RBDCRD binding is much slower than the one reported for dimers in the Packer et al. study. To test if RBDCRD binding induces dimers or higher order multimers, we measured diffusion of KRAS after treatment with a small molecule, BI2582, which based on a crystal structure solved by Ingelheim and coworkers, forms non-functional dimers of KRAS^29^. Later our colleagues identified that the BI2852 compound stabilized KRAS dimer forming a complex of four molecules–two BI2852 compounds and two KRAS molecules^30^. The dimer formation was confirmed with multiple biophysical and biochemical techniques such as size exclusion chromatography and mass spectrometry^30^. We also tested how KRAS diffusion differs in the presence of a multimer-inducing crosslinking reagent, DSSO, which crosslinks any surface exposed primary amines on the protein surface. We have previously showed a dose-dependent increase in confined diffusion of KRAS from which we infer infer cluster formation after treatment with DSSO on a simple POPC/POPS bilayer^17^.

**Fig. 5:**
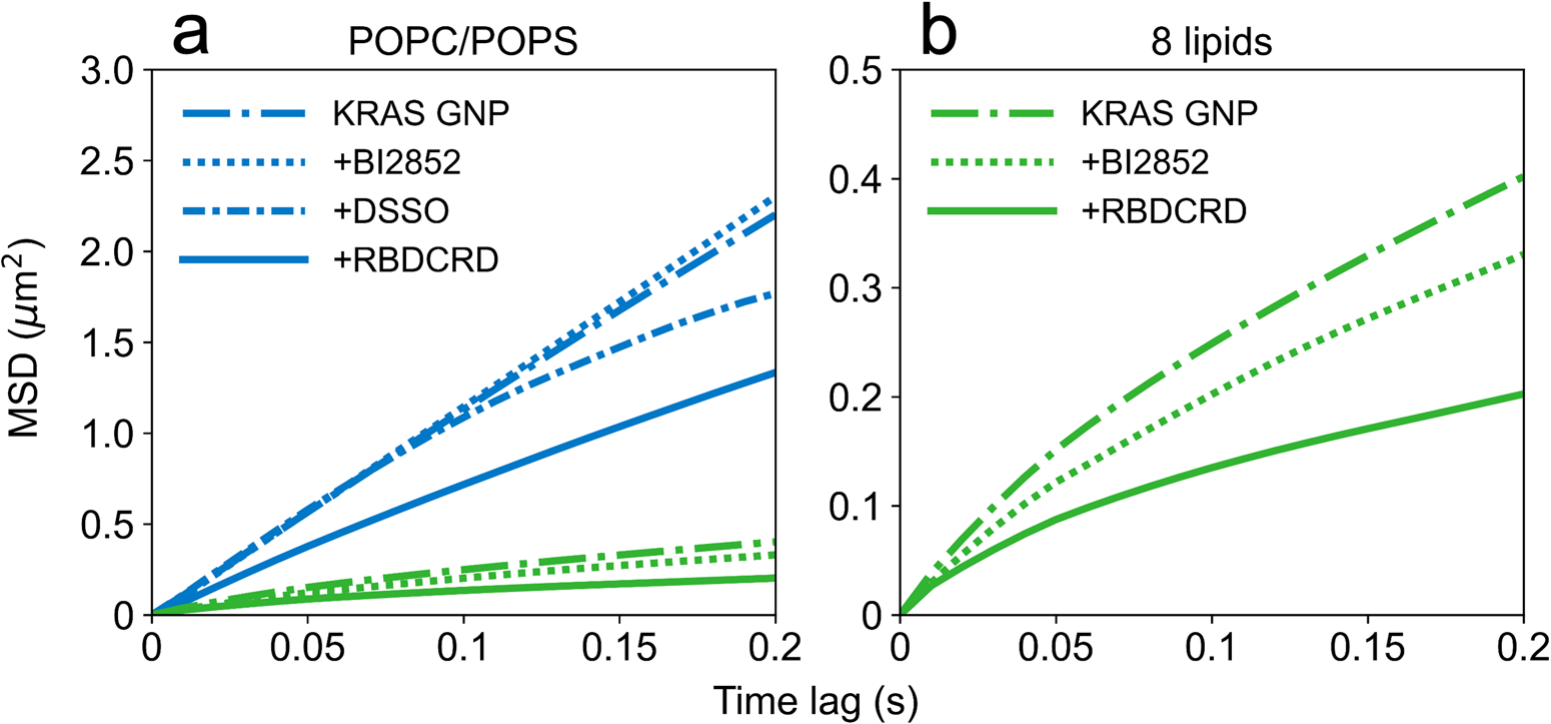
Diffusion analysis of KRAS in the presence of the dimer-inducing compound, BI2852, and clustering reagent, DSSO, compared to KRAS by itself and in the presence of RBDCRD. **a** Mean square displacement plots of KRAS on 2-lipid (blue) and **b** 8-lipid (green) bilayer. The Fig. 5b is KRAS diffusion on a 8-lipid bilayer shown on different y-axis. KRAS diffusion without any treatment is represented by long-dash-dot line and after treatment with BI2852 is represented by a dotted line, DSSO by short-dash-dot line, and RBDCRD by a solid line. The corresponding raw data with associated errors are tabulated in Supplementary Table 6, 7.

We carried out SPT experiments on the POPC/POPS and the 8-lipid bilayers to compare the diffusion behavior of KRAS in various multimeric states on a simple and more complex lipid system (Fig. 5). In agreement with our previous study, KRAS showed monomeric Brownian diffusion on the 2-lipid bilayer but heterogenous, confined diffusion on the 8-lipid bilayer^17^. When we treated KRAS on a simple POPC/POPS bilayer with BI2852, we observed no change in the MSD plot of KRAS, whereas addition of DSSO increased the confinement of KRAS as represented by a curved line in Fig. 5a. On the other hand, the binding of RBDCRD to KRAS on the 2-lipid bilayer did not increase the curvature of the MSD plot (as indicated by the lack of curvature) but reduced the apparent diffusion coefficient. These data show that dimer formation induced by the small molecule BI2852 does not lead to a change in diffusion behavior on a 2-lipid system; however, higher-order clusters induced by the cross-linking reagent or RBDCRD are detectable on a 2-lipid system.

In contrast, on the more complex 8-lipid bilayer (Fig. 5b), KRAS on its own showed confined diffusion. After treatment with BI2852, the MSD plot of KRAS showed even greater confinement, and our data show that confinement of KRAS is more prominent when bound to RBDCRD, suggesting higher-order clusters are highly dependent on the lipid environment. By comparing these graphs, we can see that the slow diffusion upon RBDCRD binding is not a result of KRAS dimer formation, but rather, the additive effect of RBDCRD interaction with KRAS and with the membrane, perhaps resulting in greater membrane viscosity and/or restriction of the diffusion paths through an excluded volume effect.

#### Macro model and CG simulation predicts larger number of KRAS-RBDCRD clusters

Single-molecule tracking studies provide the possibility of monitoring the kinetics of intracellular processes, such as cluster formation. However, because of the limitation posed by the resolution of an optical microscope, we cannot determine the number of molecules in a cluster in tracking experiments. To bridge this gap, we used the MuMMI infrastructure that covers and transitions between three simulation scales and can capture biological processes occurring at micro to femtosecond timescales and across micron to single atom or Ångstrom length scales. The simulation parameters are described in more detail in the Materials and Methods section.

**Fig. 6:**
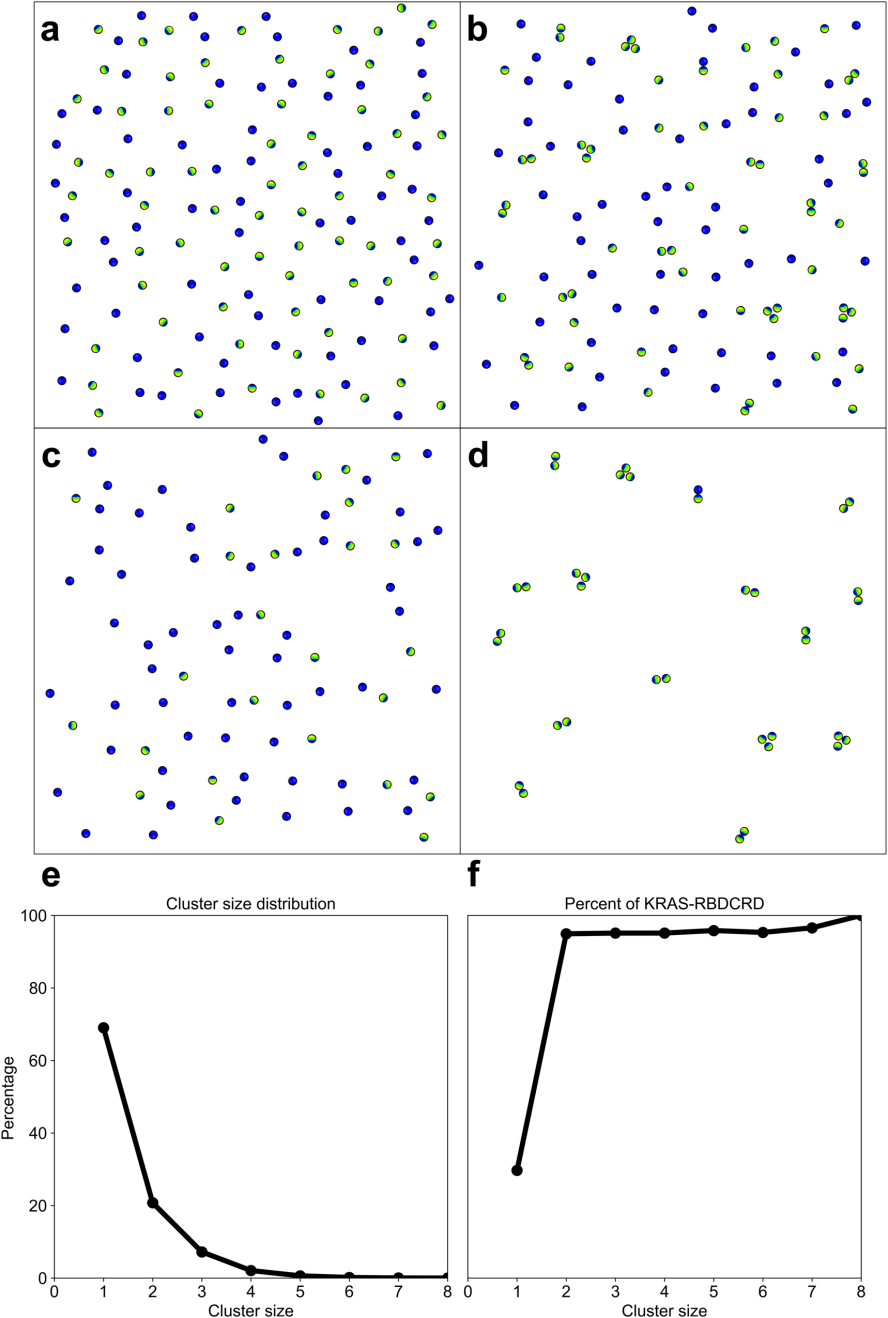
A graphical illustration depicting macro model simulations of KRAS and KRAS-RBDCRD. **a** Representation of the macro model starting positions of KRAS (blue) and KRAS-RBDCRD (blue-green-yellow) and **b** their equilibrated configurations. The illustrative example in **b** is also shown as the separate relative populations of the protein monomers (**c**) and aggregates (**d**). Analysis of the macro model cluster characteristics to show the distribution of cluster sizes (**e**), as well as the average percentage of KRAS proteins within each cluster size that are bound to RBDCRD (**f**).

The continuum macro model simulation comprises a 1000 nm X 1000 nm area of bilayer and 300 randomly placed protein molecules, of which ~50% are KRAS and ~50% are KRAS-RBDCRD (Fig. 6a). There are (*extremely* low) transition rates for the proteins to switch between KRAS and KRAS-RBDCRD (to represent the RBDCRD arriving or leaving), which depend on protein state but is independent of the local lipid environment. In this macro model there are no implicit protein-protein attractions, but each protein type (KRAS or KRAS-RBDCRD) and orientational states interacts uniquely with the lipids in the membrane. Thus, as the proteins diffuse along the freely standing 8-lipid bilayer, this type of simulation can model protein colocalization into somewhat persistent clusters due to their interactions with and reorganization of the lipid environments. This phenomenon is only observable due to the larger and longer size- and timescales sampled in the continuum macro model as compared to, for example, particle-based simulations.

The results are depicted in Fig. 6a–d where the lipid-induced distributions of cluster sizes rapidly decays. After equilibration, an average of ~70% of the KRAS proteins exist as monomers, and there is a negligible number of instances of a cluster larger than five proteins (Fig. 6e). It is worth emphasizing again that there are no direct attractive forces between the proteins in this model, and this level of spatial colocalization is due to interactions with the lipids. Indeed, we can observe significantly more protein colocalization when the lipid-protein interactions are increased (with a peak multimeric aggregate size of ~4-5 KRAS Supplementary Fig. 3). To further characterize the composition of the different clusters we calculated for each cluster size what percentage of proteins in that cluster size are complexes of KRAS-RBDCRD (Fig. 6f, graphically illustrated in Fig. 6c, d). Of all the KRAS that exist individually by themselves as a monomer, only ~30% of the KRAS are bound to RBDCRD (Fig. 6c, f). Remarkably, of all the KRAS that are colocalized into *any* multimeric cluster, ~90–95% of the KRAS are bound to RBDCRD (Fig. 6d, f). Thus, when aggregated with any other KRAS, the protein is almost always bound to RBDCRD. Similarly, the macro model with increased lipid-protein interactions displays almost no monomeric KRAS-RBDCRD, and plateaus at ~80–90% of KRAS in clusters larger than two bound to RBDCRD. Thus, the in silico investigation into the protein-mediated lipid interactions show that the lipid environment around KRAS-RBDCRD causes a ~20-fold increase in the probability of being colocalized (within 5 nm of another KRAS) compared to the lipid environment around RAS only.

**Fig. 7:**
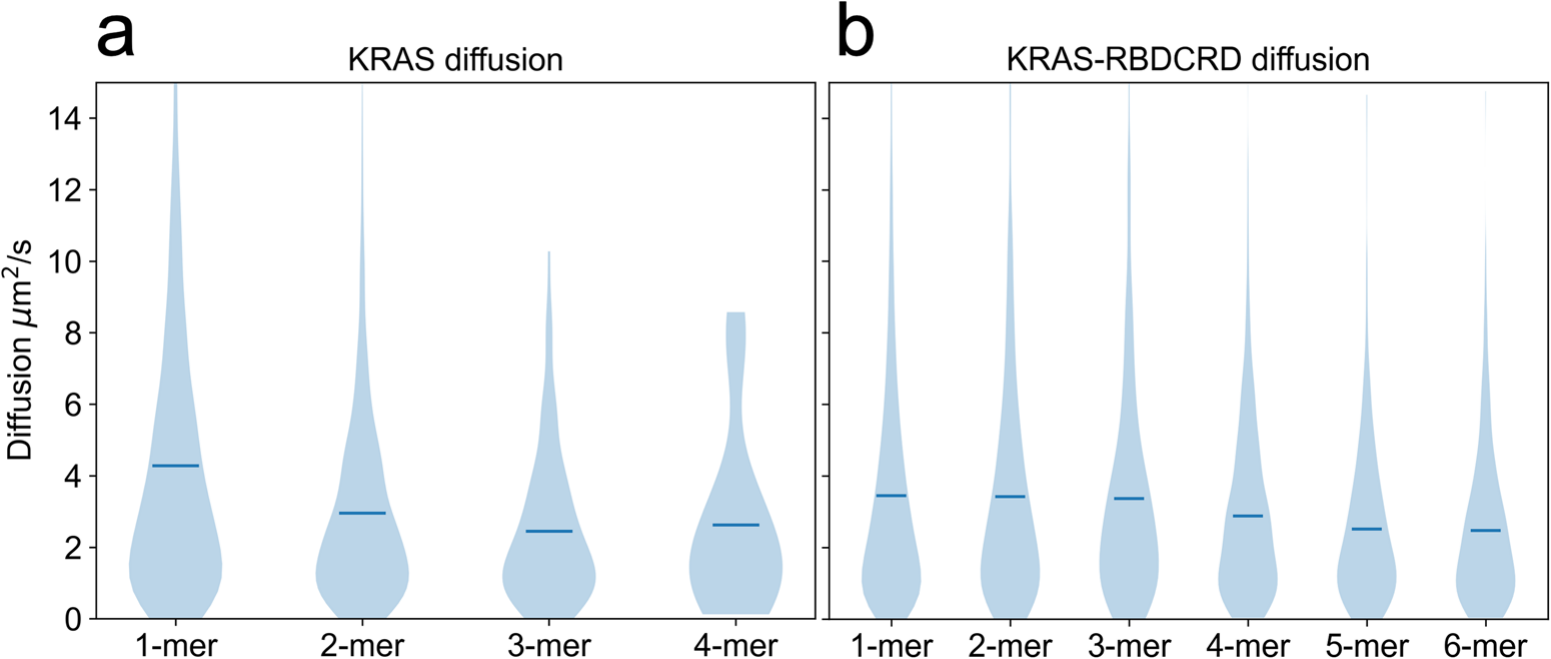
Diffusion analysis of different protein sizes from the CG simulations. **a** The calculated MSD values for the various clusters comprised of KRAS and **b** KRAS-RBDCRD only identified from the CG simulations. The violin plots show the distributions of diffusion and the mean value (blue line).

While the large-scale macro model simulations provide us the more global properties of the nanocluster populations and distributions, the finer-detailed CG simulations are better suited for calculation of molecular scale properties of the clusters. As such, we used analyses of the CG simulations to determine the lateral diffusion values of nanoclusters of homogenous composition (i.e., all KRAS or all KRAS-RBDCRD). Despite the fact that diffusion calculations from simulation data are often extremely noisy and variable, diffusion analyses showed consistent trends; as the size of the nanocluster increased, the lateral diffusion decreased (Fig. 7a), and the addition of RBDCRD decreases the lateral diffusion of KRAS (Fig. 7b). This is consistent with the increase in the slow state population in the HMM analysis of KRAS in presence of RBDCRD (Fig. 2, Supplementary Table 2). The KRAS diffusion data fits to a decay curve (Supplementary Fig. 5), as is the expected correlation between protein size and membrane diffusion^31^. KRAS-RBDCRD diffusion did not allow for such fitting – likely due to both insufficient sampling of the simulations and additional complexities associated with diffusion calculation of the more intricate, multi-domain systems.

It is worth noting that the calculated diffusion from the KRAS-only 4-mer simulations appears *faster* than the 3-mer, which counter to what we would expect, and differs from the trends seen in the rest of the data. However, the distribution of the data for the 4-mer is unusual in that it does not have a decaying tail like all the other plots, but almost a bimodal distribution (Fig. 7a, Supplementary Fig. 4). In our simulation, there are few 4-mer simulations (~30, compared to >3,000 for 1-mer), thus this is likely a statistical aberration, potentially due to some outliers (see Supplementary Fig. 4 for further details). If we exclude the few outlier data points, the average calculated diffusion for 4-mers is 1.74 µm^2^/s compared to 2.45 µm^2^/s for 3-mers.

### Mechanistic insight into RBDCRD membrane interaction

**Fig. 8:**
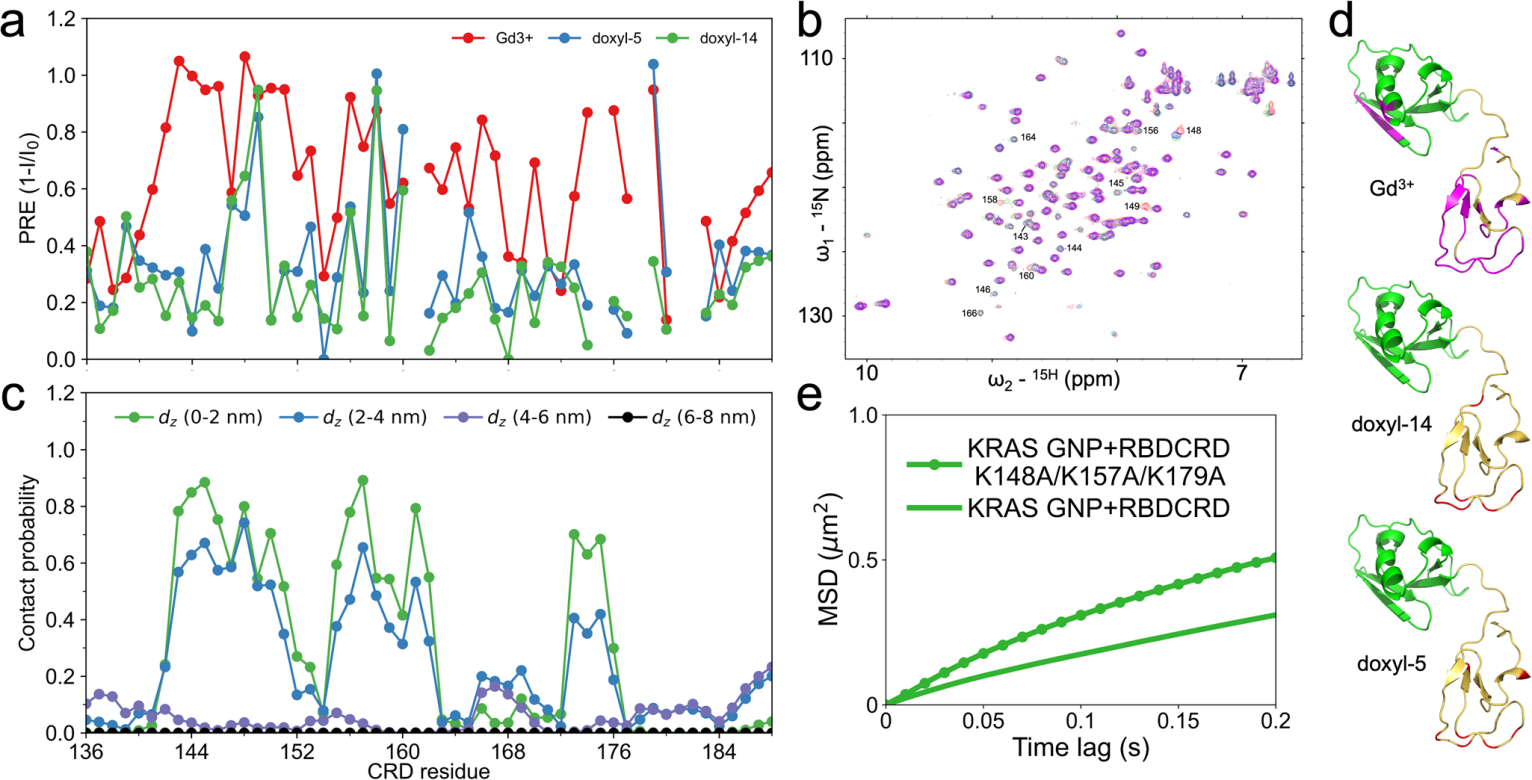
Mechanistic insights into RBDCRD-membrane interactions. **a** Dot plot representation of NMR-PRE ratios (I/I0) plotted as (1-(I/I0) to compare with contact probabilities obtained from the MuMMI AA simulations. **b** HSQC spectra showing reduction in intensities of CRD residues of RBDCRD bound to DMPC:DMPS bicelles (red) upon the addition of doxyl-5 (blue), doxyl-14 (green) and Gd^3+^ (magenta). **c** Contact probability distribution of CRD residues in KRAS-RBDCRD as a function of the distance between the COM of hydrophobic/cationic CRD loop residues and the COM of the bilayer along the bilayer normal (dz), obtained from MuMMI AA simulations. **d** CRD residues with PRE ratios of ≤50% are mapped onto a model of RBDCRD: doxyl-5 and doxyl-14 (red) and Gd^3+^ (magenta). **e** Mean square displacement plots of KRAS on 8-lipid bilayer when bound to wild type RBDCRD (solid) and mutated RBDCRD K148A/K157A/K179A (dotted). The corresponding raw data with associated errors are tabulated in Supplementary Table 8.

To gain mechanistic insight to the molecular interaction between RBDCRD and the membrane, we performed NMR experiments with ^15^N labelled RAF1 RBDCRD in the presence and absence of bicelles composed of DMPC/DMPS (70/30). When bound to bicelles, almost the entire CRD domain (residues 137–185) showed considerable marked peak intensity reduction, (Supplementary Fig. 6A). Strong chemical shift perturbations in residues F146, L147, K148, L149, A150, F151, L159 and L160 were observed in the presence of bicelles (Supplementary Fig. 6B). These residues correspond to the two flexible loop regions within the CRD that have previously been identified as interacting with lipid bilayers^27,32,33^. The residues in the RBD domain showed negligible chemical shift perturbations (Supplementary Fig. 6B), hence only CRD residues were considered in further analysis. To gain further insight into how these two loop regions engage with the lipid bilayer, we performed NMR Paramagnetic Relaxation Enhancement (PRE) experiments. In these experiments, we prepared bicelles with a gadolinium (Gd^3+^) spin label chelated on the lipid headgroup, or with lipids containing doxyl spin labels on the fifth (doxyl-5) or fourteenth (doxyl-14) acyl carbon. Spin labels at different positions within the lipid bilayer can provide information on the relative depth of insertion of residues within RBDCRD^34^. The PRE ratios of the residue-specific intensities in the presence and absence of bicelles containing spin labels were calculated (Fig. 8a, Supplementary Fig. 7, 8). A decrease in signal intensity indicates that residue is in proximity (20–30 Å) to the spin label^35^.

In our experiments, most of the CRD residues lose significant signal intensity when gadolinium is on the lipid headgroup, indicating that the CRD is within 30 Å of the lipid headgroup (Fig. 8a). In addition, R67, T68 and V69 within RBD also show a decrease in intensity (Supplementary Figs. 7, 8), confirming earlier observations that these residues spend some time adjacent to the lipid headgroup^33,36^. Interestingly, when the doxyl spin labels are placed on the fifth carbon of the acyl chain, only residues L147, K148, and L149 in loop 1 and Q156, F158, and L160 in loop 2 from CRD show a significant signal decrease, suggesting the backbone of these residues are inserting into the acyl chains of the bilayer (Fig. 8a, b). The signal of these same residues is also attenuated when the doxyl spin label is on C14 of the acyl chain, providing further support to the interpretation that these residues can penetrate the lipid bilayer. Moreover, in the doxyl-14 sample within loop 1, L149 and loop2, F158 have the largest signal decrease indicating the deepest insertion into the lipid bilayer, whereas K148, L147 from loop 1 and F156, L160 from loop 2 have more modest signal loss, suggesting they are unable to penetrate the bilayer as deeply. Fig. 8d shows the models of RBDCRD mapped with the results from PRE using different spin labels. Together these data show that loops 1 and 2 are responsible for the interaction of CRD with the lipid bilayer, with residues L147, K148, L149, Q156, F158, and L160 making the strongest contacts.

In our NMR-PRE experiments, we were unable to analyze a complex of KRAS bound to RBDCRD as the size of the complex caused the ^15^N/^1^H cross peaks to broaden severely. To compliment the NMR results, we used the last parts of all MuMMI AA simulations (>40 ns) of KRAS and RBDCRD bound to 8-lipid bilayer and assessed the contacts between CRD residues from 136 to 188 and the lipid bilayer. We considered CRD residues to be in contact with lipid membrane if the distance between the center of mass (COM) of a Cα atom of a residue and P atom in the lipid headgroup is less than or equal to 0.85 nm. Contact probability was calculated by summing the probabilities of having more than zero contacts.

**Fig. 9:**
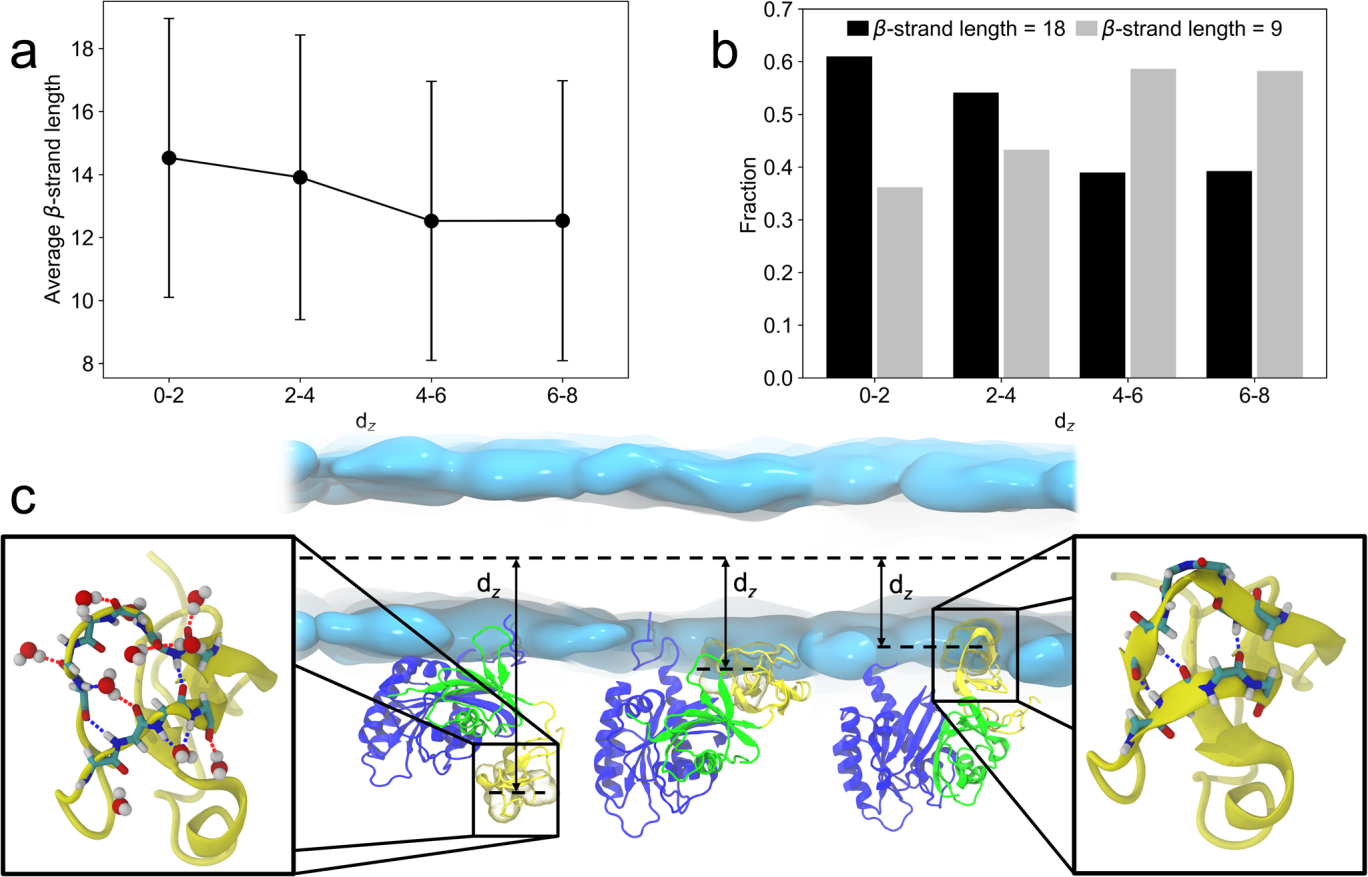
The secondary structure of CRD changes with membrane insertion. **a** The average β-strand length in loops 1 and 2 of the CRD as a function of dz. **b** Histogram of the populations of β-strand length as a function of dz. **c** Example orientations of the KRAS-RBDCRD complex at different dz values. KRAS is shown in blue, RBD in green, and CRD in yellow. The headgroups of the lipid bilayer leaflets are shown as cyan surfaces. Examples of hydrogen bonding networks are shown inset.

Figure 8c shows the contact probability calculated for each CRD residue at different distances between the COM of hydrophobic/cationic CRD loop residues and the COM of the bilayer along the bilayer normal (d_z_) (see Fig. 9c for a visual representation of d_z_). The d_z_ of 0–2 nm spans from the center of the lipid bilayer to cover almost all of the lipid tails and aligns with the NMR PRE experiments performed with doxyl-5 lipids and doxyl-14 lipids. The d_z_ of 2–4 nm includes the outermost layer of the lipid headgroups and a small part of the lipid tails which mostly aligns with NMR experiments with gadolinium spin label on the lipid headgroup. Lastly, the d_z_ of 4–6 nm and 6–8 nm represents RBDCRD structures that are not in contact with the lipid bilayer. We found the CRD contact probabilities get lower with longer d_z_. The comparison between changes in intensities from NMR measurements and contact probabilities shows consistent results, especially in the highest intensity change/contact probability regions. For example, within d_z_ of 0–2 nm, both NMR and AA simulation identify high contacts between the residues along the two flexible CRD residues (144 to 150, 158, 159 and 176). At longer distances between the CRD and the bilayer (d_z_ = 2–4 nm), there is overlap between residues 149, 159 and 176 whereas there is the least overlap at the largest distances (d_z_ = 4–6 nm and d_z_ = 6–8 nm) since this d_z_ is beyond the realm of PRE experiments. A subtle discrepancy between simulation and experiments are due to the absence of KRAS in the NMR-PRE experiments. Additionally, our NMR experiments use bicelles composed of 2-lipids whereas the simulation studies use the more complex 8-lipid system, which alters the lipid acyl chain packing and may also contribute to these differences and hence greater variation.

To test if these CRD-membrane contacts impact the lateral mobility of the KRAS-RBDCRD complex on the membrane, we performed SPT studies of KRAS bound to RBDCRD with mutations in the residues K148A/K157A/K179A. The residues were selected based on the intensity changes in NMR, high contact probability with the lipid bilayer in AA simulation, and mutations that would neutralize the charge. Clearly, the mutations greatly alter the MSD plot of the complex as shown in Fig. 8e. The diffusion is faster and less confined compared to the wild type RBDCRD.

### Secondary structure changes in the CRD loop upon membrane embedding

To assess the influence of CRD-lipid interactions on the overall structure of the KRAS-RBDCRD complex, we next examined the secondary structure of the CRD domain (residues 141 to 164) as a function of the z-distance from the center of the lipid membrane (d_z_) in MuMMI AA simulations, see Fig. 9c. Specifically, an average β-strand length was obtained by calculating the total length of β-strands in the loop 1 and 2 of the CRD region of each structure and averaging over all the structures found within bin size of 2 nm (d_z_). A total of 3,067,000 structures from 306.7 microseconds of MuMMI AA simulations were analyzed. The average β-strand length was found to increase as the CRD loops lie deeper (smaller d_z_) into the membrane (Fig. 9a). In addition, the distribution of the secondary structures demonstrates that the structures with the longer β-strands in the CRD loops become the most populated state as the CRD region gets closer to the center of the membrane (Fig. 9b and Supplementary Fig. 9). Therefore, the secondary structure of the CRD loops strongly depends on the local environment. The elongation of the secondary β-strand structure upon membrane insertion could be the molecular mechanism for the formation of stable KRAS-RBDCRD complex on the membrane.

## Discussion

In order to initiate signaling in the MAPK signaling cascade, membrane-bound and active RAS recruits autoinhibited RAF from the cytoplasm, releasing it from its autoinhibited state, and enabling RAF to form fully active dimers. Recently, Martinez Fiesco et al. ^6^ superimposed a cryo-EM structure of the full-length, autoinhibited, monomeric BRAF-14-3-3 complex on a KRAS structure and proposed that RAS-RAF binding is a highly dynamic event. According to their model, BRAF binds to KRAS by forming high-affinity ionic bonds via the exposed basic residues in the RBD region (R158, R166, K183, R188), thus creating a steric clash and electrostatic repulsion between RAS and 14-3-3 at the RBD-14-3-3 interface. This steric clash results in partial dissociation of BRAF from 14-3-3 and releases CRD from its sequestered conformation, making it available to bind to RAS and the membrane to further stabilize the RAS-RAF complex on PM. The dynamic rearrangement also orients the kinase domain in a suitable conformation for dimer formation. However, the structural studies did not include the plasma membrane and hence the role of membrane in RAS mediated activation of RAF remains uncharacterized.

In this study, we aim to elucidate the molecular mechanism of RAF1 activation by KRAS on the cell membrane. Our focus is on investigating the molecular interactions between fully processed KRAS and the RBDCRD domain of RAF1, which is reconstituted onto the artificial membrane bilayer. Our findings reveal that the binding of RBDCRD to KRAS promotes slower diffusion of the complex, and that this is particularly pronounced when diffusing on an 8-lipid membrane.

Utilizing tool compounds that either facilitate dimerization or induce higher-order clustering through crosslinking of primary amines, we deduce that the slower diffusion is a result of nanoclustering of KRAS-RBDCRD complexes, rather than dimerization. Macro model and coarse-grained simulations of KRAS and RBDCRD on an 8-lipid bilayer confirm cluster formation with slower diffusion rates compared to KRAS alone.

Additional insights from AA simulations and NMR PRE experiments demonstrate a direct interaction between key residues on CRD region and the lipid bilayer. Mutations in these residues led to faster diffusion of the complex compared to the wildtype RBDCRD. Furthermore, AA simulations predict secondary structure changes in the CRD region upon membrane insertion. Together, the results underscore the significant role of the membrane, not only in spatially clustering RAS-RAF complexes, but also in the stabilization of the complex via CRD-membrane interaction thereby promoting nanoclustering.

In an earlier study on simple a DOPC/DOPS bilayer, Packer et al., showed that RBD binding reduces the diffusion of KRAS from 4 µm^2^/s by half to 2 µm^2^/s and promotes KRAS dimerization^28^. In contrast, our study includes both the RBD and the membrane binding CRD domain which enhances the effector interaction with both KRAS and membrane. Our studies also differ in terms of the membrane composition. In this study, we use a more complex 8-lipid composition, as we have previously demonstrated its ability to replicate a cell-like three-state diffusion of KRAS, and it more closely resembles the composition of the inner leaflet of plasma membrane^21^. Using the Hidden Markov Modelling analysis of single particle trajectories of KRAS molecules, we show three-state diffusion classified into the fast (~4 µm^2^/s), intermediate (~0.7 µm^2^/s) and slow diffusion (~0.16 µm^2^/s) states. Our analysis reveals that only a small percentage of KRAS exhibits fast diffusion, with the majority diffusing in the intermediate and slow states when bound to RBDCRD. We do not observe the diffusion rate of 2 µm^2^/s attributed to dimer diffusion in the Packer study.

The differences between our study and the Parker study merit some discussion. On an isotropic two-dimensional fluid, like the 2-lipid POPC/POPS bilayer, the lateral diffusion is only weakly dependent on the hydrodynamic radius (R) of the protein (D~ ln(1/R)). Hence formation of dimers is insufficient to bring significant change in the shape of the MSD plot and the apparent diffusion coefficient. Confined diffusion arises when the protein concentration is large enough to impede diffusion by increasing the overall membrane viscosity and/or restricting diffusion paths by forming clusters through an excluded volume effect^37,38^. Using the simple 2-lipid POPC/POPS bilayer, we tested the two scenarios. We created artificial non-functional KRAS dimers using the BI2852 and KRAS nanoclusters using the crosslinker molecule DSSO. KRAS dimers showed no significant change in the MSD plot, whereas cross-linked KRAS nanoclusters showed confined diffusion (Fig. 5). When bound to RBDCRD, KRAS diffusion became significantly slower and more confined, indicating formation of clusters rather than dimers. The effect gets more prominent as we increase the complexity of membrane by using a 8-lipid composition. This result highlights two major findings (i) RBDCRD binding promotes nanoclustering and (ii) that the membrane composition directly contributes to the extent of nanoclustering.

Previous electron microscopy experiments have shown that KRAS nanoclusters selectively enrich negatively charged PS and PA lipids but not phosphoinositides or cholesterol lipids^10^. Similarly, in atomic force microscopy experiments, KRAS proteins partitioned into cholesterol-poor liquid disordered domains and formed nanoclusters that were capable of binding to RBDCRD domains of different RAF isoforms^39^. Here, using various lipid compositions, we show that both electrostatic interaction and the heterogeneity of the membrane plays a role in RBDCD binding and nanocluster formation. First, before adding RAF1, we show that KRAS diffusion on its own is regulated by the membrane composition. KRAS remains monomeric with very fast diffusion (~3 µm^2^/s) on a simple 2-lipid POPC/POPS bilayer, in agreement with previous publications. However, if we replace the POPS with PIP2 lipids, KRAS now shows confined diffusion with a mixture of fast (3 µm^2^/s) and slow (~0.74 µm^2^/s) moving states. The enhanced interaction between the positively charged lysine residues in the HVR of KRAS and the negatively charged PIP2 could lead to the clustering of KRAS proteins. Secondly, as we introduce more complexity into the membrane by incorporating diverse lipids with varying acyl chains and saturations, we observe a systematic increase in the population of slower-moving KRAS. This increase surpasses the proportion of the fast state observed when measured on the 8-lipid composition. Although we do not directly observe phase separation or domain formation of the 8-lipid mixture in the TIRF experiments, it is likely that the inclusion of cholesterol may create transient, mesoscale domains that promote RAS clustering. Upon RAF1 binding, the fraction of slower-moving KRAS increases dramatically in all lipid compositions and is higher when bound to the tandem RBDCRD domain compared to RBD only. Similarly, the ensemble diffusion is most confined in the case of KRAS-RBDCRD complexes diffusing on the most heterogeneous 8-lipid mixture. This suggests that the combination of CRD interaction with the membrane and the degree of membrane heterogeneity favors clustering behavior.

In the pursuit of understanding the molecular details of RAS activation of RAF, Tran et al. recently solved the first crystal structure of GTPase domain of KRAS(1-169) and RAF1 RBDCRD (52-188) ternary complex and showed that RBDCRD binds to KRAS as one structural entity linked together by a short linker, and not as distinct domains^5^. In binding studies, the isolated CRD as a standalone domain does not exhibit binding to KRAS proteins. However, when the CRD coexists with the RBD, forming the RBDCRD domain, there is a notable increase in its binding affinity to KRAS. We also observe similar effects in our single particle tracking imaging experiments. CRD on its own has minimal effect on the diffusion of KRAS, while the tandem RBDCRD domain dramatically alters KRAS diffusion on the membrane. They also aligned their high-resolution crystal structure of KRAS-RBDCRD onto previously published MD simulations of KRAS on an anionic lipid bilayer composed of POPC and 30% POPS to predict membrane-interacting CRD residues. Based on their model, they observed that few key residues such as K144 and L160 in the hydrophobic loop of CRD insert into the membrane, in agreement with NMR studies of CRD only on nanodiscs^32^. To further elucidate on RBDCRD interaction with the membrane, we also employed NMR PRE experiments of the tandem RBDCRD bound to bicelles to define the key interactions between CRD residues and the lipids. These biophysical measurements were complemented by an analysis of our AA simulations. Phospholipids with spin labels systematically positioned at the headgroup and different lipid tail position act as molecular rulers to identify residues in CRD that insert deep into the lipid bilayer. More specifically, the hydrophobic residues L147, K148, L149, F158, L160, N161, C176 in the loop 1 and loop 2 region show enriched interaction with the membrane in both NMR and AA simulation structures. These identified residues are also in general agreement with previously reported NMR and simulation studies^26,32,33,40^. Correspondingly, charge reversal mutations in K148A/K157A/K179A of the RBDCRD hindered the membrane interaction and completely changed the diffusion pattern of the complex. The diffusion became faster and less confined compared to the wild-type RBDCRD. Altogether, the results emphasize that CRD-membrane interaction is a key mechanism in creating slow-moving KRAS-RBDCRD complex on the membrane. Additionally, our AA simulation predicts that the insertion of the hydrophobic residues in the lipid bilayer stabilizes the longer β-strands, thus anchoring the CRD domain into the membrane and favoring cluster formation.

In silico modelling of our system in the MuMMI macro model and CG simulations identified a distribution of monomers and multimers of KRAS-RBDCRD complexes, and convincingly supports our nanoclustering hypothesis. First, the large-scale continuum model simulation demonstrates that the lipid environment around KRAS-RBDCRD causes a ~20 fold increase in protein colocalization compared to the lipid environment around KRAS-only. This indicates that the addition of RBDCRD can cause an increase in colocalization via membrane reorganization such that the proteins will stay in the same vicinity longer than for KRAS by itself. There is the possibility that lipid composition-induced colocalization could bring the proteins close together for the stronger, more direct protein-protein contacts to take over once the CRD encounters KRAS and membrane lipids. Secondly, the CG simulation data also shows that the presence of RBDCRD and increased protein aggregation both decrease the lateral diffusion of the complex. Thus, we propose a mechanism that the binding of RBDCRD makes the ‘binding target’ (of KRAS) bigger, slower moving, ‘stickier’ (due to the lipid environments), and easier to ‘hit’. From a signaling perspective, these factors act to enhance the local concentration of the protein and increase the chance of a ‘reaction’ occurring where multiple KRAS form a nanocluster. In concordance with these simulation results, a logarithmic decay fit of the experimental diffusion coefficients and the CG simulation values predicts cluster sizes of greater than 8 protein complexes with diffusion rates corresponding to intermediate and slow diffusion in the SPT experiments. This cluster size is in agreement with the RAS nanoclusters reported on PM sheets^9^ but smaller than the clusters of KRAS-RBDCRD observed on artificial DOPC/DPPC/DOPS/Cholesterol (20:45:5:25) lipid bilayer via atomic force microscopy^39^. A direct extrapolation of the cluster size based on the experimental diffusion coefficient is not possible because of the differences in the experimental setup of the in silico and in vitro studies. For example, in MD simulations, the lipid bilayers are freely standing whereas in SPT experiments, the lipids in the bilayer are diffusing on a glass coverslip, therefore impacting the physical forces such as drag force and viscosity, which may enhance the slower mobility. Likewise, in cells the hierarchical and complex nature of PM impinges on free diffusion of lipid and proteins. Nonetheless, these results are striking and demonstrate the power of combining a state-of-the-art multiscale simulation with quantitative biophysical and imaging experiments.

**Fig. 10:**
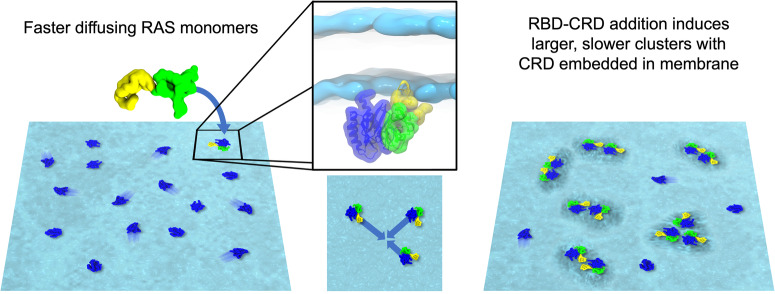
A pictorial representation depicting enhancement of nanoclusters upon RBDCRD binding to KRAS and the subsequent reorganization of lipid environment surrounding the clusters. The shadows around each RAS molecule in blue represents faster diffusion for monomeric KRAS. The absence of shadow around KRAS (blue) and RBDCRD (green-blue) complex indicate slower diffusion of the complex. The grey shadow underneath KRAS-RBDCRD nanoclusters represents lipid reorganization.

Recently, super-resolution fluorescence localization experiments have demonstrated that membrane receptor proteins, such as B cell receptors (BCR), undergo clustering upon membrane insertion. This clustering leads to the reorganization of the surrounding membrane into an ordered membrane domain. Notably, this collective behavior has the potential to initiate BCR activation^41^. The size and the stability of domains positively regulate the accessibility of the BCR receptors and hence the extent of activation and the effect on downstream signaling. In terms of MAPK signaling, no such direct observation of membrane domains composed of signaling complexes in live cells has been reported. However, our experiments do implicate membrane composition-dependent clustering of proteins which is enhanced upon effector binding. Hence, based on the integrated in silico and in vitro findings from our current and previous work, we propose a model to describe how membrane mediates activation of RAF by RAS on the PM, depicted in a pictorial representation in Fig. 10. The positively charged lysine residues and the farnesyl tail in the HVR of KRAS localizes RAS to the membrane and reorganizes the local lipid environment creating a “lipid fingerprint” that promotes nanoclustering. KRAS in this lipid fingerprint is not only concentrated but also more active and accessible to the RBD domain of the RAF1:14-3-3 complex. Once RBD binds and 14-3-3 dissociates, the CRD domain is then free to associate with RAS and membrane via direct insertion of hydrophobic residues. This further alters the local membrane environment as well as stabilizes the longer β-strand secondary structure in the CRD loops. This weak lipid-protein and protein-protein interaction enhances the size and stability of preexisting nanoclusters and increases the near-neighbor interaction between two RAF kinase domains leading to dimerization.

## Methods

### Supported lipid bilayer

Small unilamellar vesicles (SUVs) were prepared using the sonication technique^42^. Briefly, the desired lipid compositions shown in Supplementary Table 1 were aliquoted into a clean 4 mL glass vial. The chloroform was dried off the lipids using a gentle stream of argon gas and then the vial was placed in a lyophilizer overnight to get rid of any residual solvents. The dried lipid mixture was then hydrated in 1 mL of 20 mM HEPES and 200 mM NaCl buffer for 1 h at room temperature. The mixture was then mixed vigorously using a vortex mixer for 5 min and then subjected to 20 freeze-thaw cycles using liquid nitrogen and a warm water bath. The mixture was then sonicated in a room temperature water bath for 90 min or until the milky solution turned clear. The SUVs were then collapsed on a plasma cleaned #1.5 coverslips fitted into the Bioptechs FCS2 chamber (Bioptechs, Butler, PA). The vesicles were incubated at room temperature for an hour and then washed with 10 mL of vesicle buffer. 1 mL of the desired protein mixture was then flowed through the chamber, incubated for an hour, and then washed off with the protein buffer (20 mM HEPES, 300 mM NaCl, 5 mM MgCl_2_). Prior to imaging, the samples were buffer exchanged to the imaging buffer (20 mM HEPES, 300 mM NaCl, 5 mM MgCl_2_ and 50 mM β-mercaptoethanol)

### Bicelles

Bicelles were prepared as followed: 50 mM 1,2-dimyristoyl-sn-glycero-3-phosphocholine (DMPC) and 1,2-dihexanoyl-sn-glycero-3-phosphoserine (DMPS) in chloroform stocks containing either 1,2-dipalmitoyl-sn-glycero-3-phosphoethanolamine-N-diethylenetriaminepentaacetic acid (gadolinium salt) Gd^3+^ (16:0 PE-DTPA Gd), 1-palmitoyl-2-stearoyl-(5-doxyl)-sn-glycero-3-phosphocholine (16:0 Doxyl-5 PC) and 1-palmitoyl-2-stearoyl-(14-doxyl)-sn-glycero-3-phosphocholine (16:0 Doxyl-14 PC) spin labels at a molar ratio of (70:30, 69:30:1 and 69:30:1) were aliquoted into glass vials and dried under the gentle stream of argon gas in a hood and placed on a vacuum lyophilizer for 24 h. Dried lipids were resolubilized with 100 mM 1,2-dihexanoyl-sn-glycero-3-phosphocholine (06:0 PC (DHPC)) dissolved in water to obtain a molar ratio of [(DMPC/DMPS)/with Doxyl-5 or Doxyl-14/ DHPC] *q* = 0.5.

### DNA for protein production

Expression clones for the production of RAF(52-131)^43^, His6-MBP-tev-RAF1(136-188)^32^, His6-MBP-tev-RAF1(52-188) (Addgene #159697^44^), His6-MBP-tev-RAF1(52-192), His6-Halotag-tev-Hs.RAF1(52-131)^44^, and His6-MBP-tevZ-GG-Hs.KRAS4b (2-188)^45,46^ have been described previously. Expression clones for the production of His6-Halotag-tev-RAF1(136-188), His6-Halotag-tev-Hs.RAF1(52-131) and His6-Halotag-tev-RAF1(52-188) were created as described in ref. ^44^. For the production of GG-Hs.KRAS4b(2-188) S106C/C118S as a farnesylated/methylated protein in insect cells and RAF1(52-188) K148A/K157A/K179A, also in the baculovirus expression system, Gateway Entry clones were generated by ATUM (Newark, CA). Entry clones were transferred to baculovirus expression clones containing aminoterminal His6-MBP (maltose-binding protein) fusions by Gateway LR recombination (Thermo Fisher Scientific, Waltham, MA) into pDest-636 (baculovirus, Addgene #159574). Final baculovirus expression clones were used to generate bacmid DNA in strain DE95 Grose et al. using the Bac-to-Bac system (Thermo Fisher Scientific, Waltham, MA). An expression clone for the production of GG-Hs.KRAS4b(2-188) as a farnesylated/methylated protein in insect cells under the control of the p10 promoter was made using Multisite Gateway^47^.

### Protein expression

His6-MBP-tev-RAF1(136–188), His6-Halotag-tev-RAF1(136-188), and His6-Halotag-tev-RAF1(52-188) were expressed as described in ref. ^32^ with modifications. Specifically, for His6-MBP-tev-RAF1(136–188), a 300 mL of overnight MDAG culture was used to inoculate 15 L of LB medium in a 20 L BioFlow IV bioreactor (Eppendorf/New Brunswick Scientific, Edison, NJ), with an airflow of 15 LPM and an agitation rate of 350 RPM and for His6-Halotag-tev-RAF1(136-188) and His6-Halotag-tev-RAF1(52-188), 1.0 L expression cultures were grown in 4 L baffled flasks. His6-MBP-tev-RAF1(52-192) was expressed as described in ref. ^32^ with modifications. Specifically, *Vibrio natriegens* was the expression host (VMAX X2, Telesis Bio, San Diego, CA), incubation was 30 °C for all culture steps, the overnight seed medium was ZYM 20050^48^ plus 1.5% w/v Instant Ocean Sea Salt (Instant Ocean, Spectrum Brands, Blacksburg, VA), minus lactose, shaken at 250 RPM. After 12.5 h, the 50 mL seed culture was used to inoculate 2L of ZYM 20050 medium plus 1.5% (w/v) NaCl, agitation rate of 481 RPM, airflow of 2.5 LPM, induction at an OD_600_ of 6.0 with 1.0 mM IPTG, and induction time of 8.0 hr. For ^15^N or ^13^C/^15^N incorporation, His6-MBP-tev-RAF1(136–188) and His6-MBP-tev-RAF1(52-188) were expressed as described in ref. ^32^ using a 300 ml overnight culture, the collected pellet was resuspended in 100 mL of Mod M9 medium and used to inoculate 15L of Mod M9 medium in a 20 L BioFlow IV bioreactor (Eppendorf/New Brunswick Scientific, Edison, NJ), with an airflow of 15.6 LPM, and an agitation rate of 350 RPM. His6-MBP-tev-Hs.RAF1(52-131) was expressed as described in ref. ^48^ (auto-induction protocol ZYM medium). For ^13^C/^15^N incorporation, His6-MBP-tev-Hs.RAF1(52-131) was expressed as described in ref. ^32^ for His6-MBP-tev-RAF1(136–188) with modifications. Specifically, to scale to 15 L, the overnight *E. coli* seed was grown in 300 mL of MDAG medium, the collected pellet was resuspended in 100 mL of Mod M9 medium and used to inoculate 15 L of Mod M9 medium (without addition of zinc chloride) in a 20 L BioFlow IV bioreactor (Eppendorf/New Brunswick Scientific, Edison, NJ), with an airflow of 15.6 LPM, and an agitation rate of 350 RPM. His6-Halotag-tev-RAF1-RBD(52-131) was expressed as described in ref. ^44^ Expression of His6-MBP-tev-GG-Hs.KRAS4b (2-188) (for both the polyhedrin promoter and the p10 promoter constructs), His6-MBP-tev-GG-Hs.KRAS4b(2-188) S106C/C118S, and His6-MBP-tev-RAF1(52-188) K148A/K157A/K179A was as described in ref. ^49^.

### Protein purification

Farnesylated KRAS proteins were purified as described^45^. RAF1(52-192) and RAF1(52-188) were purified as described^44^. His6-Halotag-RAF1(52-131) was purified as described^44^. His6-Halotag-tev-RAF1(136-188) was purified as described in ref. ^50^ for lysis and the initial IMAC steps (Protein production section, page S-4, in Supporting Information). The IMAC pool was dialyzed to 20 mM Tris-HCl, pH 8.5, 100 mM NaCl, and 1 mM TCEP and purified by anion exchange chromatography using a HiTrap Q HP column (Cytiva, Marlborough, MA), eluting the protein in a 10 column volume gradient from 0.1 to 1.0 M NaCl. The pooled protein was subjected to purification by SEC as outlined in ref. ^50^ and then further purified by a round of cation exchange chromatography using a HiTrap SP HP column (Cytiva), eluting the protein in a 10 column volume gradient from 0.15 to 1.0 M NaCl. The final pool was dialyzed to a buffer of 20 mM HEPES, pH 7.3, 150 mM NaCl, and 1 mM TCEP. His6-Halotag-tev-RAF1(52-188) was also purified as described in ref. ^50^ for lysis and the initial IMAC step (Protein production section, page S-4, in Supporting Information). The IMAC pool was dialyzed to 20 mM MES, pH 6.0, 75 mM NaCl, and 1 mM TCEP and purified by cation exchange chromatography using a HiTrap SP HP column (Cytiva, Marlborough, MA), eluting the protein in a 20 column volume gradient from 0.75 to 1.0 M NaCl. The pooled protein was purified by SEC as outlined in ref. ^50^ Hs.RAF1(52-131) and Hs.RAF1(136-188) were purified as described in ref. ^50^ for RAF-RBD (52-131). RAF1(52-188) K148A/K157A/K179A was purified as described in ref. ^50^ for RAF-RBD (52-131), with modifications. Specifically, the insect cell pellet was homogenized in a lysis buffer (100 mL per liter of expression culture) of 20 mM HEPES, pH 7.4, 500 mM NaCl, 5 mM TCEP, and 10% glycerol. The 500 mM NaCl and 10% glycerol were maintained throughout the purification.

### Nucleotide exchange

KRAS proteins were exchanged into GppNHp and verified as described in refs. ^17,26^ with the addition of 5 mM MgCl_2_ in the overnight incubation step.

### Single particle tracking (SPT)

SPT movies were acquired on a Nikon NStorm Ti-81 inverted microscope equipped with an Andor iX EMCCD camera (Andor Technologies, USA). The samples were imaged with a 100x 1.49 N.A. oil immersion objective (Nikon, Japan) in TIRF mode by setting the illumination angle at 2980 degrees. Prior to imaging, the camera was cooled to –75 °C. Samples labeled with Alexa647 were excited with the 647 nm laser from the Agilent laser module at 10% power and samples labeled with atto550 were excited with the 561 nm laser from the Agilent laser module at 10% laser power. Time-lapse movies of 5000 frames were collected under continuous illumination at a 10 ms frame rate. Up to 15 movies were acquired per sample. SPT movies were analyzed as described in ref. ^17^. Briefly, the movies were first converted into tiff files in Image J^51^. Particles in each tiff file were then detected and localized using the Localizer plugin in the Igor Pro software^52^. Single particles in each frame were localized as spots based on the eight-way adjacency particle detection algorithm with generalized likelihood ratio test (GLRT) sensitivity of 10 and a point spread function (PSF) of 1.3 pixels. A symmetric 2D Gaussian fit function was used to estimate the position of the PSF in each frame. Any localized particles persistent for more than 6 frames were then linked between the subsequent frames into tracks. The particles were allowed a maximum jump distance of 5 pixels and a maximum of one blinking frame. The tracks from several movies were combined into a single Matlab file and submitted for vbSPT HMM analysis using the batch cluster at the Frederick National Laboratory. The mean square displacement plots were calculated and plotted using a script written in Matlab.

### NMR

NMR data were collected on an Agilent 800 MHz and Bruker Avance II 500, 600, and 900 MHz spectrometer at 25 °C, processed with NMRPipe^53^ and analyzed using CcpNMR^54^. The backbone resonances of RBDCRD(52-188) with and without bicelles were assigned using standard triple resonance data (HNCA, HNCACB, CBCACONH and ^15^N-edited HSQC-NOESY). Data were collected at 25 °C on 300 mM samples in a buffer containing 20 mM HEPES (pH 6.5), 300 mM NaCl, 50 mM Glutamate, 50 mM Arginine, and 1 mM TCEP-HCl. Residues 52–56, 64, 103–105, 158, 175, 178 and four prolines (63, 93, 135, 181) had no assignments. A strong peak for 161 was observed in free RBDCRD but was not detectable in the presence of bicelles. Conversely, 158 was only observed for samples with bicelles. Amide backbone assignments are shown in Supplementary Fig. 6, 7. Chemical shift perturbations were calculated as ${\triangle \delta }_{{NH}}=\scriptstyle\sqrt{(\triangle \delta {H}^{2}+{\left(\frac{\triangle \delta N}{5}\right)}^{2})/2}$, where $\triangle \delta H$ and $\triangle \delta N$ are the ^1^H and ^15^N chemical shift changes, respectively. The criterion for the selection of significantly affected residues was an average plus one standard deviation of all ${\triangle \delta }_{{NH}}$ values.

The PRE and control bicelles were prepared as followed: 50 mM DMPC/DMPS in chloroform stocks containing either Gd^3+^, Doxyl-5 and Doxyl-14 at a molar ratio of (70:30, 69:30:1 and 69:30:1) were aliquoted into glass vials and dried under the gentle stream of argon in the hood. Dried lipids were re-solubilized with 100 mM DHPC dissolved in water to obtain a molar ratio of q = 0.5. For NMR measurements, 100 μM of RBDCRD in 20 mM HEPES, 100 mM NaCl pH 7.4 was flash frozen and lyophilized to remove all liquid. The lyophilized sample was then solubilized in 600 μL of preformed bicelles and rotated gently at 4 °C for 15 min and then centrifuged to remove any precipitation. The samples were then placed in an NMR tube and HSQC data was collected on a Bruker 700 MHz instrument at 25 °C with 64 scans. To determine changes in peak intensity, peak heights were determined using POKY^55^ to obtain PRE ratios of I/I_0_, where I_0_ is peak height in the control experiment with no PRE tags. Errors for PRE ratios were calculated as,

${Error}=\frac{I}{{I}_{o}}\scriptstyle\sqrt{{(\frac{1}{S{N}_{I}})}^{2}+{(\frac{1}{S{N}_{{I}_{o}}})}^{2}}$, where SN is the signal-to-noise. An overlay of all RBDCRD bound to bicelles samples along with normalized PRE ratios are shown in Supplementary Fig. 8. PRE ratios for doxyl-5 and doxyl-14 were normalized using 1.18 and 1.32, respectively. Individual PRE ratio plots with errors are shown in Supplementary Fig. 8. Highly overlapped peaks (74, 76, 82, 83, 96, 97, and 171) and peaks with very low intensities (114, 179, and 182) were excluded from PRE analysis.

### The three-scale MuMMI RAS-RBDCRD simulation campaign

The MuMMI infrastructure was initially developed to bridge two-scales: a large and long timescale macro model simulation with an ensemble to selected higher resolution^56^ coarse-grained (CG) MD simulations^18,57^. Recently, MuMMI was greatly extended to integrate three-scales: the macro scale and CG capabilities were broadened to include All-Atom (AA) simulations to capture the atomistic details of significant events^56^. The new MuMMI was used to simulate RAS and RAS-RBDCRD dynamics on the PM^21^. The main improvements to MuMMI are: support and parameters for an additional protein (RAS-RBDCRD)^26,56^, a new AA with reliable CG-to-AA transformations^58^ to sample changes in protein secondary structure, a new machine learning sampling framework used to select simulations of interest at finer resolutions (macro to CG and CG to AA)^19^, a new faster and higher fidelity macro model^59^, and an updated workflow that is generalizable and has extend scalability and fault tolerance^60^.

The MuMMI RAS-RBDCRD simulation campaign^56^ consisted of a large macro model continuum simulation (1000 nm X 1000 nm) with ~150 KRAS and ~150 KRAS-RBDCRD proteins. The macro model samples realistic fluctuations in the local composition of the PM, KRAS and KRAS-RBDCRD colocalization, their lipid preferences, and changes in protein orientation relative to the membrane (the protein orientational state). From the running macro model, a diversity of proteins (protein number, protein orientations, and different lipid compositions) were sampled automatically by selecting 30 nm X 30 nm local patches, converting them into CG Martini^61^ representations and simulating them. Subsequently, from the CG simulations, snapshots were selected from simulations with a single KRAS-RBDCRD, sampling a diversity of protein orientations and a range of CRD insertions into the membrane. Those selected CG frames were converted from CG Martini to AA CHARMM36^62,63^ and further simulated in full atomistic detail.

#### Macro model simulation

The continuum macro model simulation consisted of a 1000 nm X 1000 nm area of bilayer that contained 8-lipid species designed to mimic an average PM^59^. 300 protein molecules (Fig. 6A) were randomly placed on this membrane. ~50% of the molecules were parameterized to represent the behavior of a KRAS protein, while the other 50% were parameterized to represent the behavior of KRAS bound to RBDCRD. The different proteins (KRAS and KRAS-RBDCRD), and their different orientational states have differing interactions with the lipids. There are transition rates to determine the probability of each orientation transitioning to a different orientation. Furthermore, there are also (*significantly* lower) transition rates for the proteins to switch between KRAS and KRAS-RBDCRD (to represent the RBDCRD arriving or leaving). These state-state and protein-protein transition rates are fixed constants that are independent of the surrounding environment. The macro model^56,59^ does not contain any implicit protein-protein attractions but can represent protein colocalization due to their interactions with the lipid environments, and how the lipids interact with each other. The simulation was run for 20.5 ms and allowed for realistic fluctuations of the lipid compositions relative to the proteins. During the large MuMMI simulation campaign, the accumulated inputs to the macro model parameters from the continuous feedback cycle over-represented certain protein-lipid interactions when evaluating protein nanoclusters. This resulted in predisposition towards protein clustering. As such, a refined version of the macro model was run without the feedback that was analyzed for cluster distributions and compositions.

#### CG simulations

From the running macro model, 30 nm X 30 nm local patches were continually sampled, selected for diversity and then converted into CG Martini^61^ simulations and run using ddcMD^64^. 34,523 local patches were selected that consisted of a range of protein numbers, protein types (KRAS vs KRAS-RBDCRD), and lipid composition. Each simulation consisted of ~140,000 CG particles and was simulated for 0–5 μs each (with an aggregated time of 97.36 ms). The detailed simulation parameters are as described in ref. ^26^.

#### AA simulations

As the CG simulations were running, each snapshot was analyzed and sampled for diversity on the basis protein orientations and how far the hydrophobic CRD loops inserted into the membrane. From the CG simulations, 9623 snapshots were selected and converted from CG Martini to AA CHARMM36^62,63^. Each simulation consisted of ~1.4 M atoms and was simulated for 0–70 ns each (with an aggregated time of 326.26 μs). The detailed simulation parameters are as described in ref. ^58^.

### Analysis of CRD secondary structures

The secondary structure of the RAF CRD region (residues 141 to 164) is computed via the DSSP algorithm using the Bio.PDB.DSSP module of Biopython^65^. A total of 306.7 microseconds of MuMMI AA simulations were analyzed using the pdb files obtained through MuMMI. The distances between the centre of mass (COM) of the hydrophobic/cationic CRD loops (residues 145–148, 158–161) and the COM of the bilayer along the bilayer normal (d_z_) are obtained from the online analysis of AA. The details of AA simulation setup and online analysis modules can be found in ref. ^58^.

### Analysis of CRD contacts

The contacts between RAF CRD (residues 136 to 188) and lipid membrane are computed using the ‘contacts’ algorithm from the ‘measure’ functionality in Visual molecular dynamics (VMD)^66^. The last parts (>40 ns) of all the MuMMI AA simulations are used to obtain contact probability distributions. A CRD residue is considered to be in contact with the lipid membrane if the COM distance between alpha Carbon (Cα) atom of a residue and Phosphorus (P) atom of a lipid molecule is less than or equal to 8.5 Å.

### Coarse-grained diffusion analysis

Diffusion of the RAS proteins on the bilayer was calculated from the CG simulations using the mean-square displacement of the molecule as a function of time as described in ref. ^26^.

### Macro model cluster analysis

A clustering algorithm was used to determine the number and size of protein clusters in each frame of the macro model. Firstly, a protein point is selected, and if it is not already assigned to a cluster, a new cluster label is created. Then we find the set of protein points that have not been assigned a cluster label, and that are within r_max_ distance (5 nm) to at least one particle already assigned to the current cluster label. These new points are then assigned to the current cluster. The point searching is repeated until no new points are found to assign to the current cluster, and the overall process is iterated until all the protein points have been assigned to a cluster.

### Statistics and reproducibility

A minimum of 15 movies, each movie composed of 5000 frames were evaluated for mean square displacement analysis. A minimum of 2 repeats per experiments were performed. Hidden Markov Modeling (HMM) analysis were performed as sets of 5 tracking movies. The diffusion coefficients in the supplementary section are presented as average and standard deviation from a minimum of 10 HMM analyses.

### Reporting summary

Further information on research design is available in the Nature Portfolio Reporting Summary linked to this article.

### Supplementary information

Peer Review File

Supplementary Material

Reporting Summary

## Data Availability

The raw data for each MSD plots are provided in the supplemental information. The raw single particle tracking movies are available on request. NMR spectrum and peak assignments are provided in the supplemental section. Raw NMR-PRE spectra are available on request. All simulation parameter and input files are made accessible at https://bbs.llnl.gov/. All simulation raw data are hosted on the NIH MoDaC server (https://modac.cancer.gov/assetDetails?returnToSearch=true&&dme_data_id=NCI-DME-MS01-18439109). Alternatively, it can also be accessed at modac.cancer.gov with the asset id: mummi_ras-raf_campaign3_052821.

## Code availability

Single particle tracking experiments were analyzed using the Localizer plugin in Igor Pro. The mean square displacement plots were obtained using a home written MATLAB code. The HMM analysis were performed using the vbSPT software package. NMR data were processed using NMRPipe and analyzed using CcpNMR and POKY. Simulations were analyzed using MuMMI, MDAnalysis (https://www.mdanalysis.org/), VMD (https://www.ks.uiuc.edu/Research/vmd/) and Biopython (https://biopython.org/).
