# Author Correction: Membrane lipids drive formation of KRAS4b-RAF1 RBDCRD nanoclusters on the membrane

**DOI:** 10.1038/s42003-024-06061-4

**Published:** 2024-04-11

**Authors:** Rebika Shrestha, Timothy S. Carpenter, Que N. Van, Constance Agamasu, Marco Tonelli, Fikret Aydin, De Chen, Gulcin Gulten, James N. Glosli, Cesar A. López, Tomas Oppelstrup, Chris Neale, Sandrasegaram Gnanakaran, William K. Gillette, Helgi I. Ingólfsson, Felice C. Lightstone, Andrew G. Stephen, Frederick H. Streitz, Dwight V. Nissley, Thomas J. Turbyville

**Affiliations:** 1 RAS Initiative, The Cancer Research Technology Program, Frederick National Laboratory, Frederick, MD 21701 USA; 2 Physical and Life Sciences (PLS) Directorate, Lawrence Livermore National Laboratory, Livermore, CA 94550 USA; 3 National Magnetic Resonance Facility at Madison, Biochemistry Department, University of Wisconsin-Madison, Madison, WI 53706 USA; 4 Theoretical Biology and Biophysics Group, Los Alamos National Laboratory, Los Alamos, NM 87545 USA

**Keywords:** Membrane biophysics, Machine learning

Correction to: *Communications Biology* 10.1038/s42003-024-05916-0, published online 28 February 2024

In the Acknowledgements section, where it reads ‘For computing support, we thank OLCF and LCstaff.Release: LLNL-JRNL-845293-DRAFT.’ should have read ‘For computing support, we thank OLCF and LCstaff.Release: LLNL-JRNL-845293’. The original article has been corrected.

This has now been corrected in both the PDF and HTML versions of the Article.

