# Supplementary material for: Membrane lipids drive formation of KRAS4b-RAF1 RBDCRD nanoclusters on the membrane
Source: Commun Biol. 2024 Feb 28;7:242. doi: 10.1038/s42003-024-05916-0 (PMC10902389; doi:10.1038/s42003-024-05916-0)
Supplement: Supplementary file 2 — Supplementary Material [file 42003_2024_5916_MOESM2_ESM.pdf]

Supplementary Information for:

Membrane lipids drive formation of KRAS4b-RAF1 RBDCRD nanoclusters on the membrane.

Rebika Shrestha, Timothy S. Carpenter, Que N. Van, Constance Agamasu, Marco Tonelli, Fikret Aydin, De Chen, Gulcin Gulten, Jim Glosli, Cesar A. Lopez, Tomas Oppelstrup, Chris Neale, Sandrasegaram Gnanakaran, William Gillete, Helgi I. Ingólfsson, Felice C. Lightstone, Andrew G. Stephen, Frederick H. Streitz, Dwight V. Nissley, and Thomas J. Turbyville

Lipids	Molecular Structure	%
POPC (16:0-18:1 PC) 1-palmitoyl-2-oleoyl-*sn*-glycero-3-phosphocholine	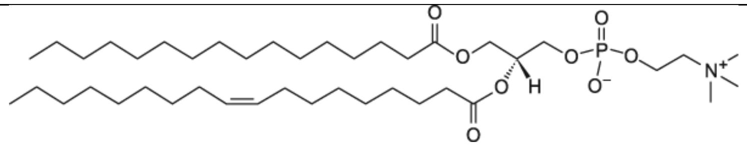	13.9
PAPC (16:0-20:4 PC) 1-palmitoyl-2-arachidonoyl-*sn*-glycero-3-phosphocholine	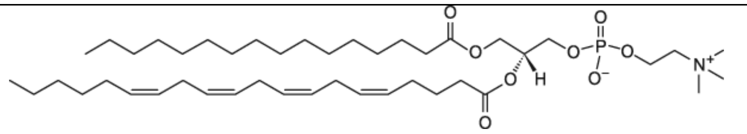	7.5
POPE (16:0-18:1 PE) 1-palmitoyl-2-oleoyl-*sn*-glycero-3-phosphoethanolamine	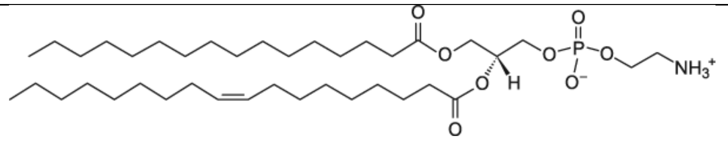	5.4
DIPE (18:2 PE) 1,2-dilinoleoyl-*sn*-glycero-3-phosphoethanolamine	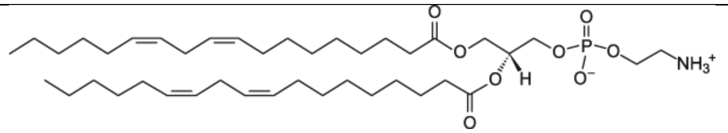	16.1
DPSM (16:0 SM (d18:1/16:0)) N-palmitoyl-D-erythro-sphingosylphosphorylcholine	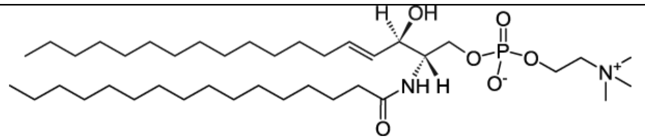	10.8
PAPS (18:0-20:4 PS) 1-stearoyl-2-arachidonoyl-*sn*-glycero-3-phospho-L-serine	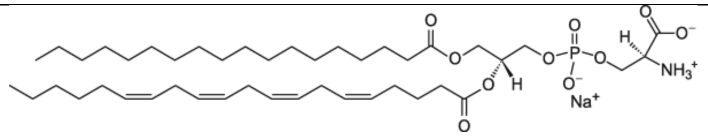	16.1
PIP2 (18:0-20:4 PI(4,5)P2) 1-stearoyl-2-arachidonoyl-*sn*-glycero-3-phospho-(1'-myo-inositol-4',5'-bisphosphate)	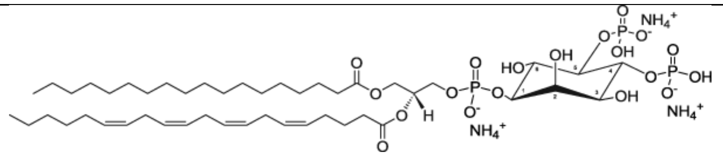	2.2
Cholesterol	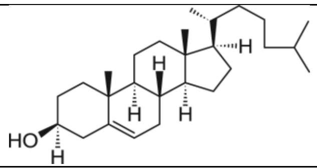	28.0

Supplementary Table 1. The lipid structure and composition used in the 8-lipid bilayer.

Lipid composition	Protein	Diffusion coefficient (µm^2/s)			Fractional Occupancy			# of trajectories
		**D1**	**D2**	**D3**	**F1**	**F2**	**F3**	
**POPC/POPS (80/20)**	KRAS only	0.11 ± 0.02	0.5 ± 0.3	3.1 ± 0.4	0.04 ± 0.02	0.06 ± 0.04	0.90 ± 0.03	103296
**POPC/PIP2 (95/5)**	KRAS only	0.16 ± 0.03	0.74 ± 0.17	3.1 ± 0.1	0.10 ± 0.06	0.16 ± 0.03	0.73 ± 0.08	56879
**6-lipids**	KRAS only	0.18 ± 0.03	1.4 ± 0.1	3.0 ± 0.1	0.010 ± 0.007	0.13 ± 0.07	0.86 ± 0.06	65712
**7-lipids**	KRAS only	0.14	1.3	2.9	0.04	0.28	0.69	86908
**8-lipids**	KRAS only	0.18 ± 0.04	0.66 ± 0.01	3.7 ± 0.2	0.2 ± 0.1	0.7 ± 0.1	0.11 ± 0.02	89236
**POPC/POPS (80/20)**	KRAS + RBDCRD	0.11 ± 0.03	0.9 ± 0.1	2.9 ± 0.2	0.19 ± 0.04	0.34 ± 0.04	0.48 ± 0.07	21187
**POPC/PIP2 (95/5)**	KRAS + RBDCRD	0.135 ± 0.001	0.84 ±0.04	3.21 ± 0.02	0.206 ± 0.008	0.296 ± 0.009	0.50 ± 0.01	50528
**6-lipids**	KRAS + RBDCRD	0.16 ± 0.03	0.55 ± 0.03	2.5 ± 0.4	0.20 ± 0.07	0.60 ± 0.06	0.21 ± 0.04	25806
**7-lipids**	KRAS + RBDCRD	0.15	0.5	2.8	0.26	0.6	0.14	14060
**8-lipids**	KRAS + RBDCRD	0.1530 ± 0.0002	0.6 ± 0.1	3.6 ± 0.01	0.45 ± 0.01	0.43 ± 0.04	0.13 ± 0.06	62471
**POPC/POPS (80/20)**	KRAS + RBD	0.14 ± 0.02	0.43 ± 0.05	3.6 ± 0.1	0.3 ± 0.1	0.24 ± 0.04	0.5 ± 0.1	15899
**POPC/PIP2 (95/5)**	KRAS + RBD	0.17 ± 0.04	0.44 ± 0.07	3.49 ± 0.06	0.058 ± 0.008	0.11 ± 0.02	0.84 ± 0.01	24848
**6-lipids**	KRAS + RBD	0.2 ± 0.1	0.99 ± 0.08	2.64 ± 0.03	0.03 ± 0.01	0.25 ± 0.01	0.71 ± 0.02	27315
**7-lipids**	KRAS + RBD	0.13	0.68	2.4	0.1	0.51	0.38	18649
**8-lipids**	KRAS + RBD	0.19 ± 0.02	0.6 ± 0.03	3.6 ± 0.1	0.42 ± 0.03	0.50 ± 0.04	0.08 ± 0.01	50305

Supplementary Table 2. Diffusion coefficients and fractional occupancies for different diffusion states of KRAS with and without RAF1 proteins on different lipid compositions calculated via vbSPT analysis. The last column shows the number of trajectories used for the diffusion analysis.

Time (s)	KRAS GPPNHP	stdev	KRAS GPPNHP + RBDCRD	stdev	KRAS GPPNHP + RBD	stdev	KRAS GPPNHP + CRD	stdev
0	0	0	0	0	0	0	0	0
0.01	0.04193	8.46E-05	0.020486	7.13E-05	0.026813	8.88E-05	0.040234	0.000137
0.02	0.085197	0.00015	0.033189	0.000117	0.044056	0.000149	0.073598	0.000235
0.03	0.12679	0.000221	0.044512	0.000162	0.059862	0.000207	0.10547	0.000336
0.04	0.16659	0.000293	0.05442	0.000205	0.074407	0.000267	0.13584	0.000436
0.05	0.20382	0.000366	0.06295	0.000245	0.087461	0.000325	0.16376	0.000533
0.06	0.23735	0.000436	0.069449	0.000281	0.098241	0.000379	0.18846	0.000624
0.07	0.26962	0.000509	0.075317	0.000316	0.10825	0.000434	0.21254	0.000717
0.08	0.30073	0.000584	0.080597	0.00035	0.11767	0.000486	0.23568	0.000814
0.09	0.33112	0.000662	0.085272	0.000381	0.12659	0.000538	0.25837	0.000912
0.1	0.36045	0.000741	0.089653	0.00041	0.135	0.00059	0.28058	0.001009
0.11	0.38905	0.000819	0.093759	0.000438	0.14286	0.00064	0.3021	0.001107
0.12	0.41735	0.000902	0.097678	0.000465	0.1504	0.000691	0.32362	0.00121
0.13	0.44496	0.000986	0.10145	0.000492	0.15745	0.00074	0.3446	0.001312
0.14	0.47222	0.001075	0.1052	0.00052	0.16426	0.000789	0.36556	0.001416
0.15	0.49892	0.001164	0.1089	0.000549	0.17074	0.000834	0.38642	0.00153
0.16	0.52523	0.001258	0.11234	0.000577	0.17701	0.000878	0.40705	0.001647
0.17	0.55117	0.001352	0.1158	0.000607	0.18329	0.000927	0.42744	0.001766
0.18	0.57696	0.00145	0.11915	0.000635	0.18971	0.000978	0.44796	0.001887
0.19	0.60244	0.001551	0.12244	0.000663	0.19612	0.001033	0.46866	0.002013
0.2	0.62765	0.001653	0.12559	0.00069	0.20256	0.001092	0.48945	0.002141
0.21	0.65277	0.001761	0.12877	0.000719	0.20901	0.00115	0.50964	0.002272
0.22	0.67758	0.001875	0.13204	0.00075	0.21534	0.001208	0.53027	0.002414
0.23	0.70203	0.001992	0.13507	0.000781	0.22154	0.001267	0.5508	0.002558
0.24	0.72608	0.002115	0.13807	0.000811	0.22764	0.001323	0.57075	0.002707
0.25	0.75018	0.002244	0.14107	0.000844	0.23316	0.001376	0.59016	0.002857
0.26	0.77404	0.002377	0.14396	0.000874	0.23852	0.001431	0.60889	0.003003
0.27	0.79744	0.002511	0.14694	0.000906	0.24373	0.001487	0.62708	0.003156
0.28	0.82053	0.002652	0.14978	0.000938	0.24866	0.001545	0.64581	0.003325
0.29	0.84408	0.002802	0.15276	0.000971	0.2537	0.001605	0.66471	0.003505
0.3	0.86732	0.002957	0.15577	0.001006	0.25849	0.001662	0.68402	0.003687

Supplementary Table 3. Data points for mean square displacement (MSD) plots of KRAS only, KRAS in presence of RAF1 RBDCRD, RBD and CRD as depicted in Fig. 3.

Time (s)	POPC/POPS	stdev	POPC/PIP2	stdev	6-lipids	stdev	7-lipids	stdev	8-lipids	stdev
0	0	0	0	0	0	0	0	0	0	0
0.01	0.10808	0.000452	0.099041	0.000221	0.12344	0.000129	0.098092	0.000102	0.04193	0.0000846
0.02	0.24325	0.001026	0.21187	0.000472	0.27129	0.000287	0.21422	0.000218	0.085197	0.00015
0.03	0.37959	0.001682	0.32428	0.000756	0.41792	0.000466	0.32879	0.000346	0.12679	0.000221
0.04	0.51516	0.002413	0.43557	0.00107	0.56444	0.000663	0.44187	0.000484	0.16659	0.000293
0.05	0.64913	0.003239	0.5444	0.001422	0.71009	0.000881	0.55286	0.000633	0.20382	0.000366
0.06	0.77829	0.004164	0.65047	0.001825	0.85444	0.001125	0.66061	0.000793	0.23735	0.000436
0.07	0.90745	0.005167	0.75414	0.002276	0.99848	0.001391	0.76759	0.000964	0.26962	0.000509
0.08	1.0349	0.006262	0.85514	0.002774	1.1423	0.00168	0.8741	0.001147	0.30073	0.000584
0.09	1.1614	0.007464	0.95408	0.003326	1.2859	0.001994	0.98058	0.001343	0.33112	0.000662
0.1	1.2895	0.008804	1.0486	0.00392	1.4286	0.002336	1.0861	0.001551	0.36045	0.000741
0.11	1.4204	0.010316	1.1401	0.004569	1.5712	0.002703	1.1919	0.001775	0.38905	0.000819
0.12	1.5527	0.011982	1.2288	0.005272	1.7135	0.003097	1.2969	0.002014	0.41735	0.000902
0.13	1.6832	0.013783	1.3113	0.006025	1.8561	0.003525	1.4011	0.002268	0.44496	0.000986
0.14	1.8144	0.015728	1.3893	0.006831	1.9971	0.003987	1.5048	0.002536	0.47222	0.001075
0.15	1.9428	0.017851	1.4626	0.007689	2.1374	0.004487	1.608	0.002823	0.49892	0.001164
0.16	2.0711	0.020301	1.5326	0.00861	2.2773	0.005022	1.7098	0.003123	0.52523	0.001258
0.17	2.2	0.022992	1.5991	0.009581	2.4167	0.005593	1.8108	0.003442	0.55117	0.001352
0.18	2.3184	0.02574	1.6639	0.010622	2.5546	0.006197	1.9111	0.003779	0.57696	0.00145
0.19	2.4317	0.02857	1.7227	0.011693	2.6915	0.006845	2.0101	0.004134	0.60244	0.001551
0.2	2.5397	0.031667	1.78	0.012825	2.8275	0.007542	2.1079	0.004504	0.62765	0.001653

Supplementary Table 4. Data points for mean square displacement (MSD) plots of KRAS only on (i) POPC/POPS, (ii) POPC/PIP2, (iii) 6-lipids, (iv) 7-lipids and (v) 8-lipids as shown in Fig. 4.

time (s)	POPC/POPS*	stdev	POPC/PIP2*	Stdev	6-lipids*	Stdev	7-lipids*	Stdev	8-lipids*	stdev
0	0	0	0	0	0	0	0	0	0	0
0.01	0.082285	0.000184	0.076371	0.000156	0.0386	0.000205	0.031753	0.000135	0.020486	0.0000713
0.02	0.17972	0.00039	0.15967	0.000323	0.07713	0.000407	0.059982	0.000246	0.033189	0.000117
0.03	0.27583	0.000618	0.24035	0.000505	0.11376	0.000617	0.086812	0.000357	0.044512	0.000162
0.04	0.37066	0.000862	0.31846	0.0007	0.14856	0.000834	0.1122	0.000468	0.05442	0.000205
0.05	0.46293	0.001122	0.39243	0.000908	0.18117	0.001052	0.13603	0.000578	0.06295	0.000245
0.06	0.5518	0.001402	0.46118	0.001134	0.21139	0.001273	0.15785	0.000687	0.069449	0.000281
0.07	0.64003	0.001699	0.52656	0.001372	0.24052	0.001498	0.17949	0.000801	0.075317	0.000316
0.08	0.72707	0.002009	0.58889	0.001619	0.26901	0.001726	0.20063	0.000918	0.080597	0.00035
0.09	0.81249	0.002337	0.64856	0.001884	0.29754	0.001969	0.22146	0.001039	0.085272	0.000381
0.1	0.89728	0.002688	0.7041	0.002158	0.32493	0.002215	0.24221	0.001167	0.089653	0.00041
0.11	0.98168	0.003067	0.75651	0.002446	0.35129	0.002456	0.26274	0.001301	0.093759	0.000438
0.12	1.0652	0.003465	0.80619	0.002748	0.37703	0.002702	0.28298	0.001444	0.097678	0.000465
0.13	1.1481	0.003885	0.85391	0.003063	0.40236	0.002964	0.30299	0.001589	0.10145	0.000492
0.14	1.2302	0.004331	0.89905	0.00339	0.42655	0.003227	0.32288	0.001745	0.1052	0.00052
0.15	1.3113	0.004797	0.94239	0.003731	0.4504	0.00349	0.34269	0.00191	0.1089	0.000549
0.16	1.3902	0.005274	0.98341	0.004076	0.47305	0.003755	0.36213	0.002083	0.11234	0.000577
0.17	1.4693	0.005776	1.0228	0.004434	0.4963	0.004051	0.38163	0.002267	0.1158	0.000607
0.18	1.5457	0.006296	1.0616	0.00481	0.51848	0.00433	0.40056	0.002458	0.11915	0.000635
0.19	1.6233	0.00685	1.0977	0.005182	0.54104	0.004617	0.41888	0.002653	0.12244	0.000663
0.2	1.6994	0.007435	1.1333	0.005562	0.56203	0.004895	0.43655	0.002847	0.12559	0.00069

Supplementary Table 5. Data points for mean square displacement (MSD) plots of KRAS plus RBDCRD on (i) POPC/POPS, (ii) POPC/PIP2, (iii) 6-lipids, (iv) 7-lipids and (v) 8-lipids as shown in Fig. 4.

Time (s)	KRAS GPPNHP	Stdev	10 mM BI2852	Stdev	1 mM DSSO	Stdev	10 mM RBDCRD	Stdev
0	0	0	0	0	0	0	0	0
0.01	0.1062	0.000138	0.1001	0.000368	0.10994	0.000376	0.068672	0.000204
0.02	0.2228	0.000346	0.2178	0.000922	0.22977	0.000813	0.14899	0.000425
0.03	0.3379	0.000631	0.3348	0.0017	0.34781	0.001308	0.22743	0.00066
0.04	0.4523	0.000986	0.451	0.0026	0.46455	0.001868	0.30356	0.000909
0.05	0.566	0.0014	0.567	0.0037	0.57873	0.002503	0.37693	0.001169
0.06	0.6796	0.0019	0.6829	0.005	0.6867	0.003247	0.44747	0.001444
0.07	0.7928	0.0025	0.7995	0.0065	0.79279	0.004079	0.51651	0.001731
0.08	0.9052	0.0031	0.9157	0.0082	0.89568	0.00499	0.58462	0.002023
0.09	1.0176	0.0039	1.0298	0.01	0.99369	0.00596	0.65178	0.002331
0.1	1.1294	0.0047	1.1449	0.0121	1.0863	0.006984	0.71727	0.002652
0.11	1.2398	0.0056	1.2605	0.0145	1.1742	0.008114	0.78205	0.002986
0.12	1.3495	0.0067	1.3768	0.0172	1.2561	0.009315	0.84663	0.003336
0.13	1.4587	0.008	1.4924	0.0202	1.3305	0.010516	0.91078	0.003707
0.14	1.5677	0.0094	1.6076	0.0236	1.4024	0.011784	0.97373	0.004093
0.15	1.6758	0.0111	1.722	0.0275	1.4729	0.013188	1.0354	0.004492
0.16	1.782	0.0132	1.8367	0.0321	1.5422	0.014698	1.0963	0.004912
0.17	1.8873	0.0158	1.9518	0.0374	1.6082	0.016286	1.1566	0.005349
0.18	1.9919	0.019	2.0685	0.0438	1.6665	0.01788	1.2168	0.005809
0.19	2.0967	0.0235	2.1848	0.0516	1.7224	0.019502	1.2755	0.006291
0.2	2.2011	0.0303	2.3021	0.0617	1.7687	0.021151	1.3333	0.006793

Supplementary Table 6. Data points for mean square displacement (MSD) plots of KRAS only and after treatment with 10 μM BI2852, 1 mM DSSO and 10 μM RBDCRD on simple POPC/POPS bilayer as shown in Fig. 5.

Time (s)	KRAS GPPNHP	Stdev	10 µM BI2852	Stdev	1 µM RBDCRD	Stdev
0	0	0	0	0	0	0
0.01	0.038171	5.75E-05	0.029402	0.000217	0.026813	8.88E-05
0.02	0.069788	9.72E-05	0.055505	0.000389	0.044056	0.000149
0.03	0.099231	0.000137	0.079619	0.000566	0.059862	0.000207
0.04	0.12656	0.000177	0.10191	0.000731	0.074407	0.000267
0.05	0.15142	0.000216	0.12169	0.000876	0.087461	0.000325
0.06	0.17311	0.000252	0.13774	0.000985	0.098241	0.000379
0.07	0.1935	0.000288	0.15442	0.001112	0.10825	0.000434
0.08	0.21282	0.000324	0.17068	0.001242	0.11767	0.000486
0.09	0.23123	0.00036	0.18664	0.001389	0.12659	0.000538
0.1	0.24883	0.000395	0.20219	0.001535	0.135	0.00059
0.11	0.26601	0.00043	0.21727	0.001682	0.14286	0.00064
0.12	0.28253	0.000465	0.23187	0.001831	0.1504	0.000691
0.13	0.29866	0.0005	0.24573	0.001984	0.15745	0.00074
0.14	0.31432	0.000536	0.25903	0.002144	0.16426	0.000789
0.15	0.32961	0.000572	0.27164	0.002308	0.17074	0.000834
0.16	0.34443	0.000607	0.28361	0.002457	0.17701	0.000878
0.17	0.35907	0.000643	0.29547	0.002612	0.18329	0.000927
0.18	0.3736	0.000681	0.30692	0.002766	0.18971	0.000978
0.19	0.38788	0.000719	0.31855	0.002929	0.19612	0.001033
0.2	0.40182	0.000758	0.3305	0.003115	0.20256	0.001092

Supplementary Table 7. Data points for mean square displacement (MSD) plots of KRAS only and after treatment with 10 µM BI2852 and 1 µM RBDCRD on 8-lipid bilayer as shown in Fig. 5.

**Time (s)**	**RBDCRD (K148A/K157A/K179A)**	**Stdev**	**RBDCRD WT**	**Stdev**
0	0	0	0	0
0.01	0.035794	0.000172	0.022988	0.00017
0.02	0.074389	0.000336	0.04392	0.000313
0.03	0.11096	0.00051	0.063756	0.000461
0.04	0.14534	0.000688	0.082429	0.00061
0.05	0.17724	0.000865	0.099617	0.000748
0.06	0.20575	0.001038	0.11524	0.000881
0.07	0.2338	0.001226	0.13082	0.001023
0.08	0.26046	0.001417	0.14599	0.001164
0.09	0.28547	0.001606	0.16046	0.001298
0.1	0.30898	0.001791	0.17454	0.001432
0.11	0.33161	0.001979	0.18839	0.001578
0.12	0.35339	0.002165	0.20242	0.001743
0.13	0.3752	0.002375	0.21673	0.001918
0.14	0.39589	0.002583	0.23036	0.002083
0.15	0.4159	0.002801	0.24412	0.002259
0.16	0.4356	0.003027	0.25767	0.002438
0.17	0.45496	0.003265	0.27094	0.002623
0.18	0.47367	0.003506	0.28399	0.002811
0.19	0.49114	0.003747	0.29684	0.002997
0.2	0.50704	0.003986	0.30971	0.003198

Supplementary Table 8. Data points for mean square displacement (MSD) plots of KRAS plus mutant RBDCRD K148A/K157A/K179A and the wildtype RBDCRD on 8-lipid bilayer as shown in Fig. 8e.

.

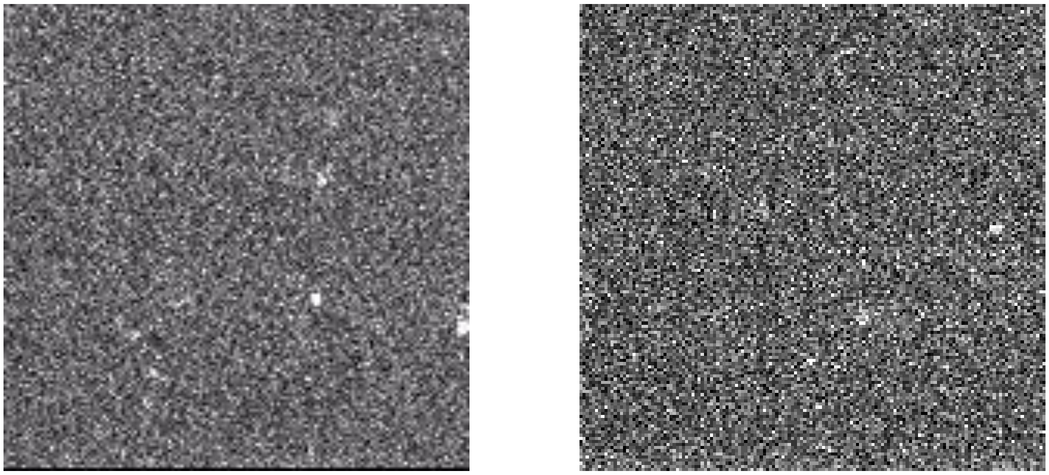

Supplementary Fig. 1. TIRF images of JF646 labeled HaloTag RBD (left) and HaloTag CRD (right) deposited onto a supported lipid bilayer composed of 8-lipid bilayer. The dimensions of the images are 150 pixel x 150 pixel (1 pixel = 0.16 µm).

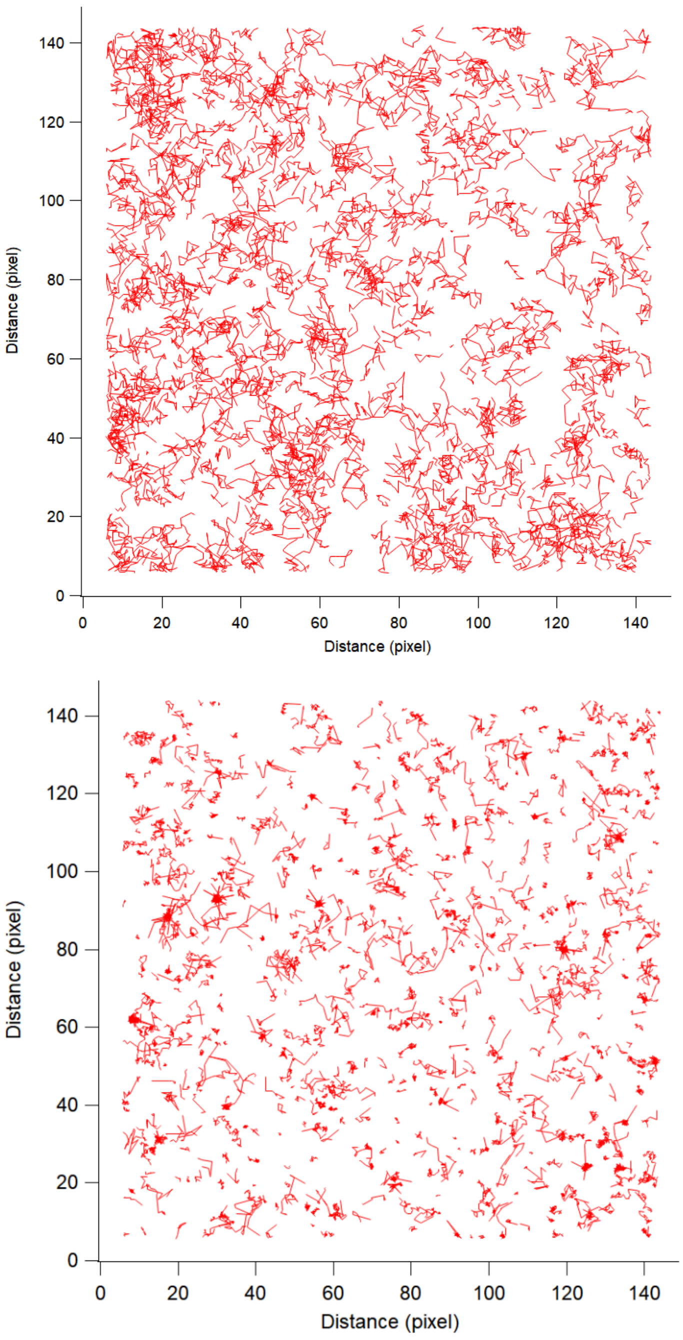

Supplementary Fig. 2. Single particle trajectories obtained for KRAS on 8-lipid bilayer before (top) and after (bottom) addition of RAF1 RBDCRD on 150 pixel x 150 pixel (1 pixel = 0.16 µm) image.

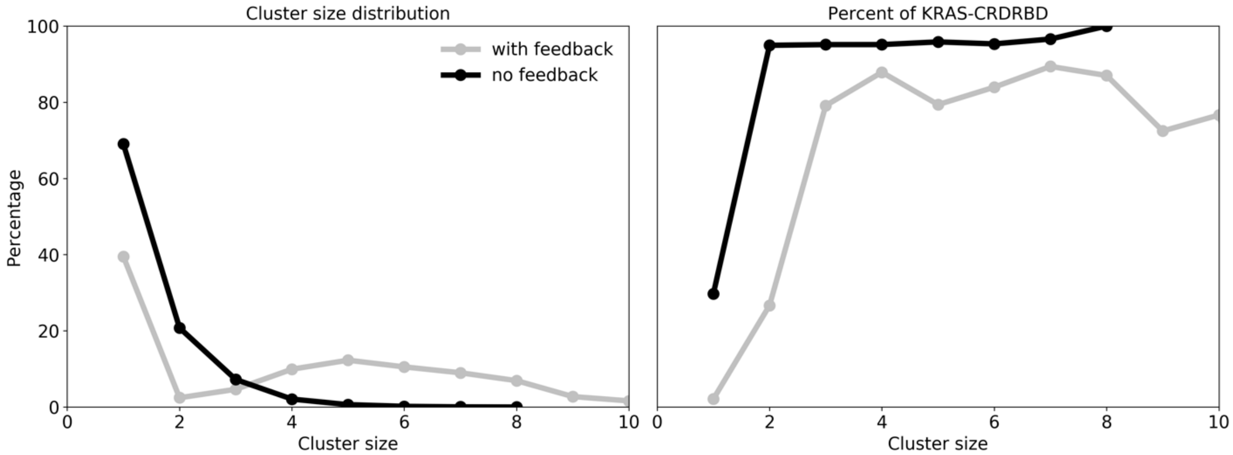

Supplementary Fig. 3. As initially described in the main methods section, during the large MuMMI simulation campaign, the accumulated ‘feedback’ inputs to the macro model parameters from the continuous analysis of the simulations over-represented certain protein-lipid interactions. This feedback loop resulted in predisposition towards protein clustering. This simulation output is plotted above (grey line) and treated as a pseudo-model to represent circumstances of increased protein-lipid interaction leading to further protein aggregation/colocalization. Compared to the version of the macro model without the feedback (black line), the increased aggregation peaks at a cluster size of ~5-6 KRAS molecules, with a similar saturating percentage of KRAS being bound to RBDCRD (~80-90%).

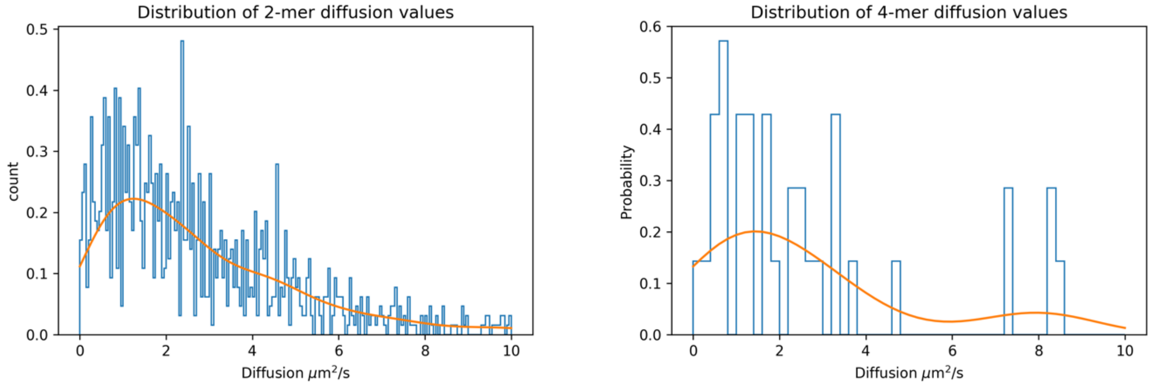

Supplementary Fig. 4. The distribution of diffusion data for 2-mer (left) and 4-mer (right) CG KRAS simulations. The raw histograms are plotted in blue, with a Gaussian kernel density estimation in orange. The distribution of the data for the 4-mer is unusual when compared to the 2-mer data (as an example), in that it doesn’t have a decaying tail like all the other plots, but almost a bimodal distribution. There are very few 4-mer simulations (~30, compared to >3,000 for 1-mer and ~1,300 for the 2-mer data). When looking in more detail at the distribution of the 4-mer diffusion data, there is a mini cluster of 3-4 simulations that seem to have unusually high diffusion when compared to the other 4-mer data, and there is a clear ‘gap’ in diffusion values such that the data falls into two categories – either <5, or >7 (see above, right). Compared to the distribution of the 2-mer diffusion values (left), the 4-mer values continue to look even more unusual. The 2-mer data shows a clear decay, and there is a continuous sampling of diffusion values, with no gaps. Thus, for some additional comparisons, we excluded the outlier simulations with higher diffusion. These outliers are likely because of sub-optimal sampling, either due to shorter simulation time that a cluster is formed, or reduced number of simulations.

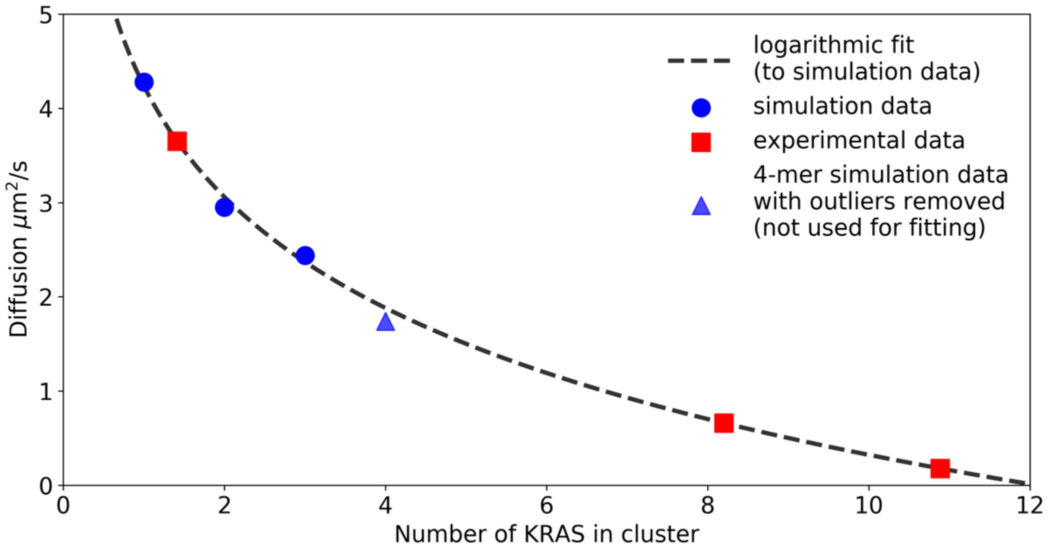

Supplementary Fig. 5. The simulation data was compared with the experimental results for KRAS-only systems. A logarithmic decay line with R^2 value of 0.99 was fitted through the simulation data for the KRAS-only clusters (as similar formalisms have shown to relate protein size and membrane diffusion [58]). The 4-mer data points were excluded due to sampling issues previously addressed in Figure S4. The KRAS-only clusters were chosen for evaluation as it gave the cleanest and most direct comparison between the protein cluster size and the diffusion values (when experimentally measuring diffusion of KRAS-RBDCRD systems, the fraction of the KRAS population bound to RBDCRD is not known). The equation of this fitted line is then used to extrapolate to a hypothetical estimate of the size of the molecular cluster that would correspond to diffusion values observed in the experiments. The exact composition of these clusters could not be determined with our experimental techniques and require further investigation. A similar, direct comparison from the computational KRAS-RBDCRD diffusion did not allow for such a prediction as the values do not decrease enough to fit to an exponential decay – likely due to both insufficient sampling of the simulations and additional complexities associated with diffusion calculation of the more complex systems.

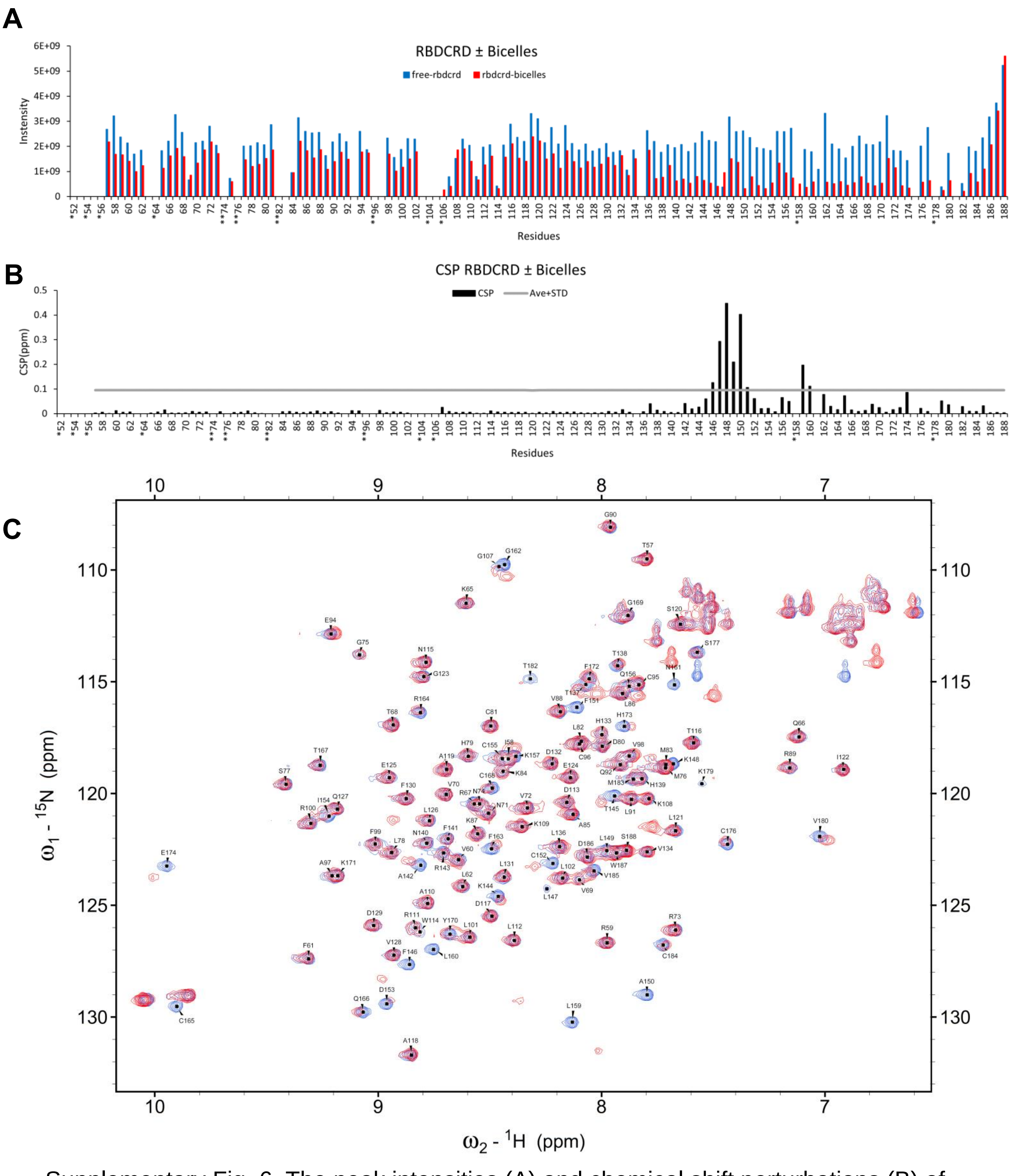

Supplementary Fig. 6. The peak intensities (A) and chemical shift perturbations (B) of RAF1 RBDCRD alone vs RBDCRD bound to 70:30 DMPC:DMPS bicelles. (C) The HSQC spectrum of free RBDCRD (blue) with amide backbone assignments and the spectrum of RBDCRD bound to bicelles (red). Residues with no assignments are marked with a single asterisk (*), and excluded overlapped peaks are marked with two asterisks (**).

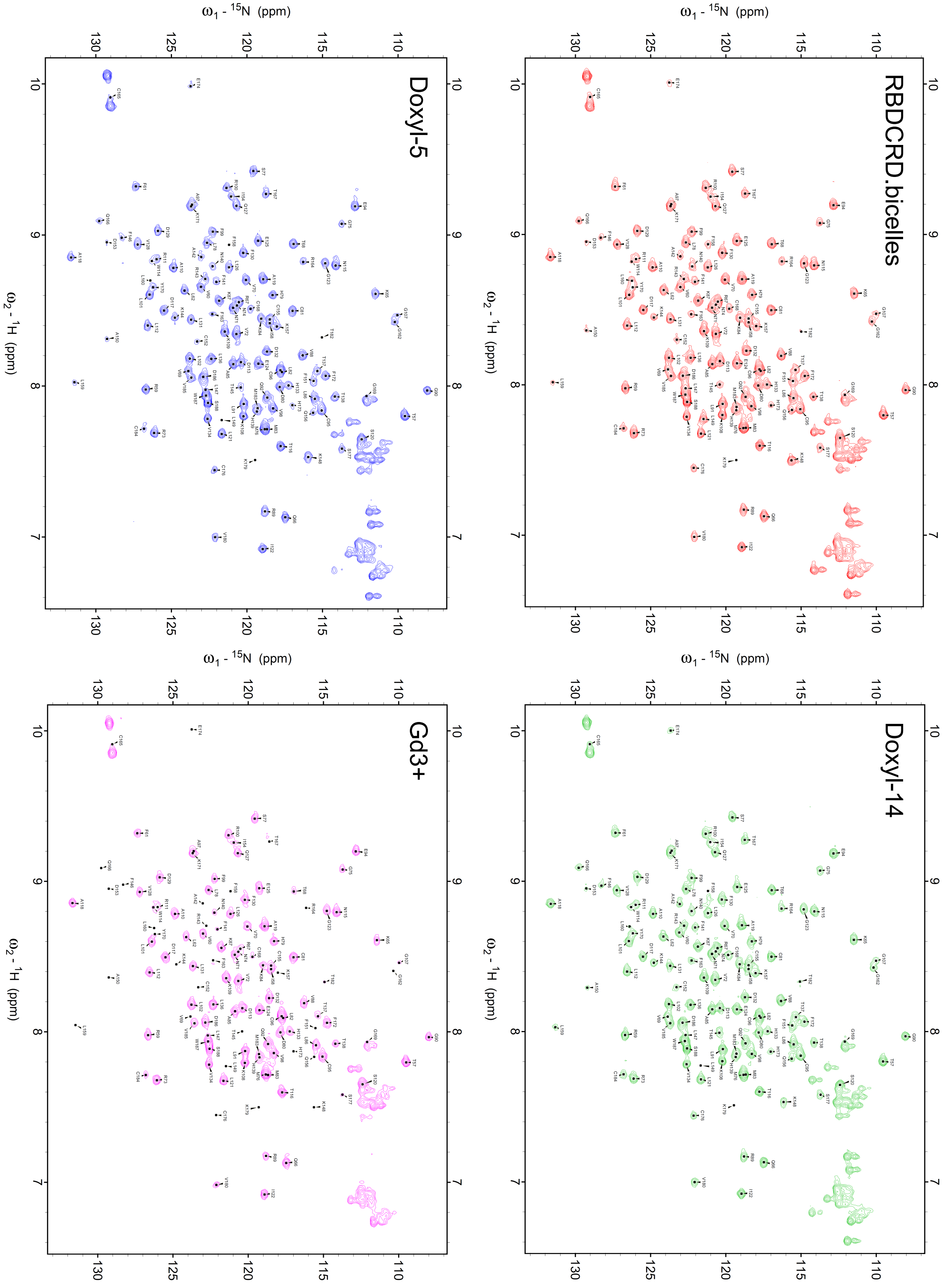

Supplementary Fig. 7. Spectra and assignments of RBDCRD bound to DMPC/DMPS bicelles with doxyl-5, doxyl-14 and Gd^{3+}.

A

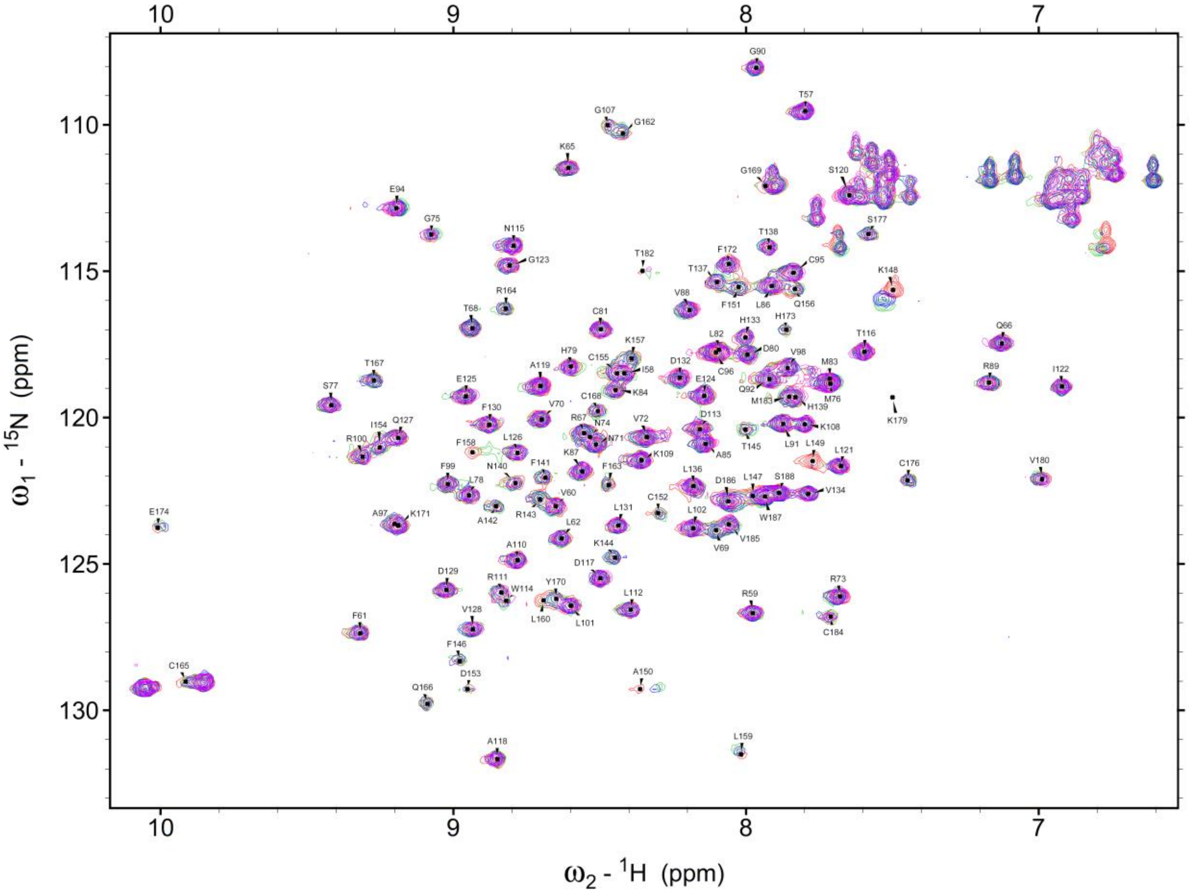

ω1 - 15N (ppm)
ω2 - 1H (ppm)

B

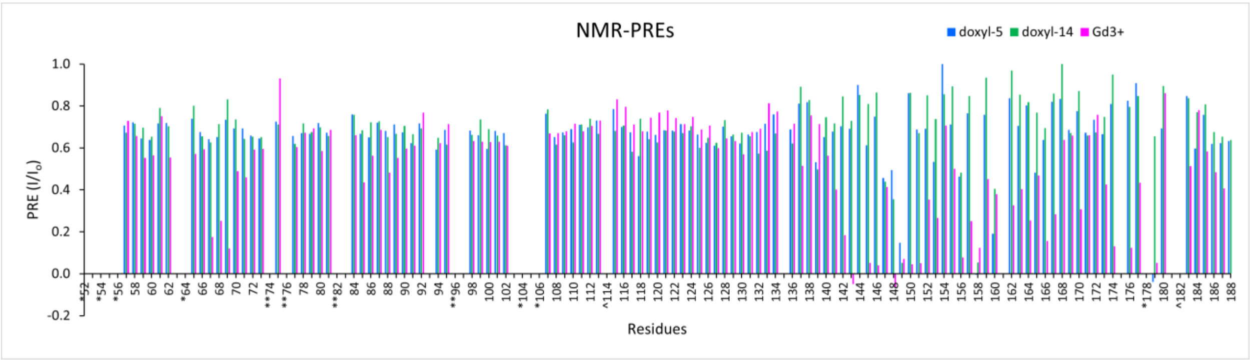

NMR-PREs
doxyl-5
doxyl-14
Gd3+
PRE (I/I0)
Residues

C

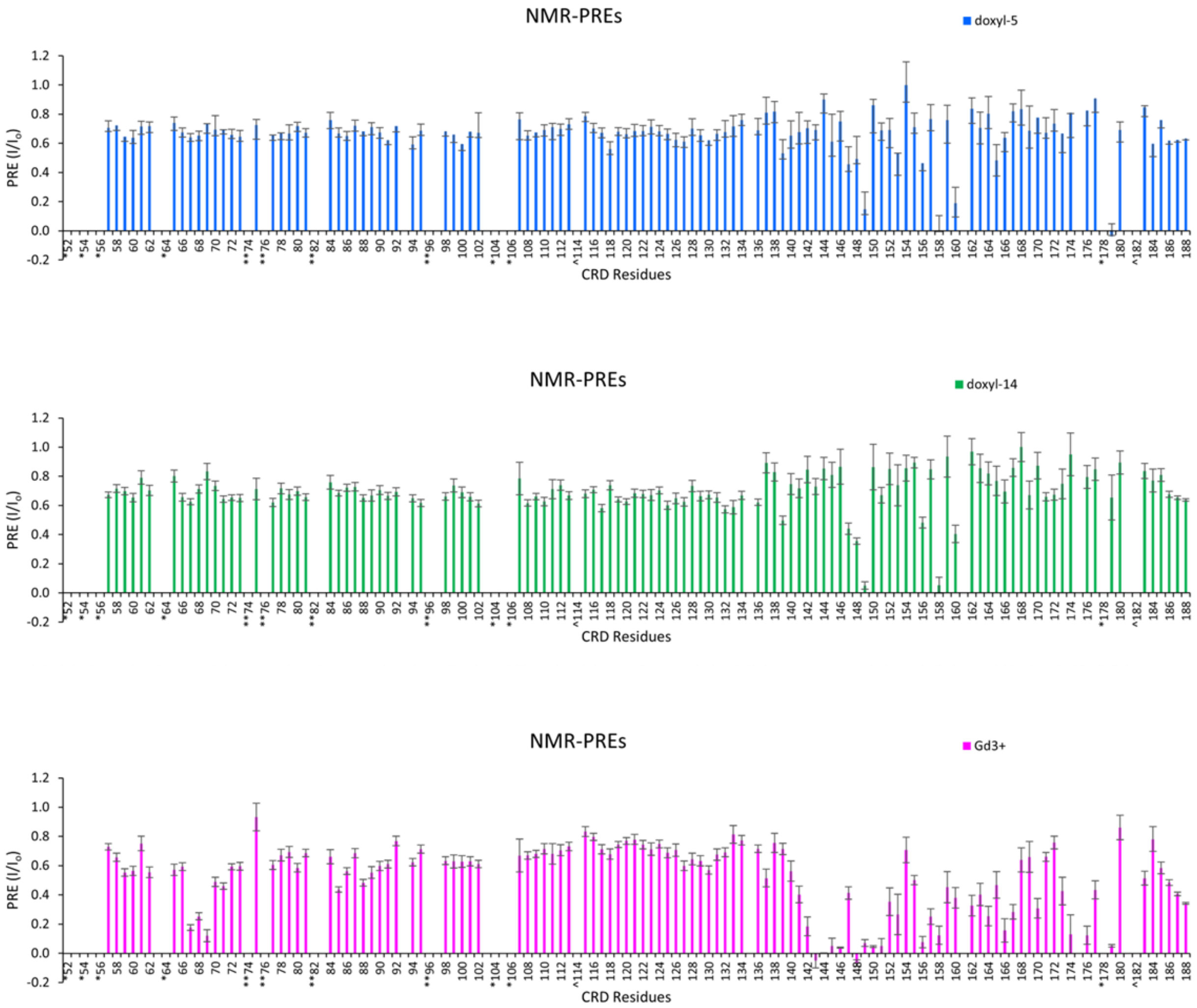

Supplementary Fig. 8. (A) Spectral overlay of RBDCRD bound to bicelles with doxyl-5, doxyl-14, and Gd^{3+} . (B) Normalized PRE ratios for RBDCRD on bicelles with doxyl-5, doxyl-14 and Gd^{3+} showing reduced PRE ratios for RBD residues 67, 68, 69 along with the majority of CRD residues. Residues with no assignments are marked with a single asterisk (*), and excluded overlapped peaks are marked with two asterisks (**). (C) Normalized PRE ratios for RBDCRD on bicelles with doxyl-5 (top), doxyl-14 (middle) and Gd^{3+} (bottom) with error bars.

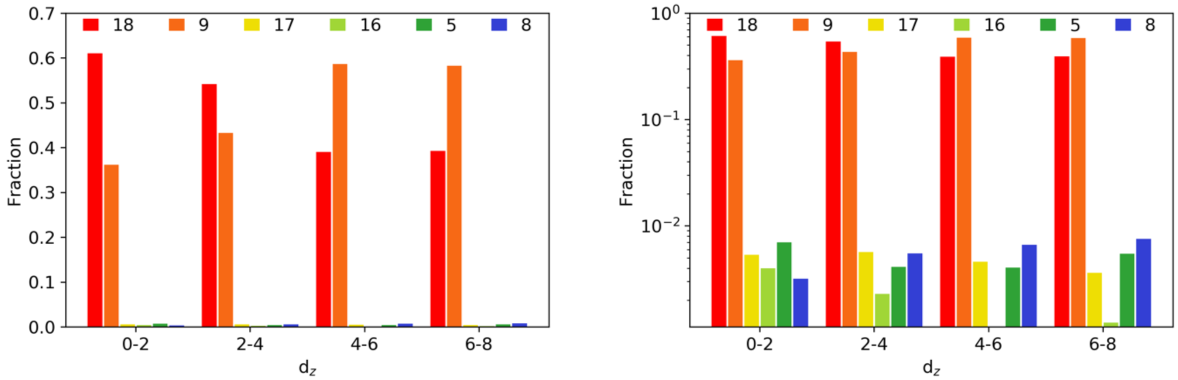

Supplementary Fig. 9. A clustering approach was employed on the total lengths of the β-strand in the loop 1 and 2 of the CRD region with bin size of 2 nm. The cluster analysis identified 6 different populations of which β-strand length of 18 (11E-7E: EEEEEEEEEEECTTTCSEEEEEEE) and length of 9 (5E-4E: EEEEECCSCCCCTTTCSCCSEEEE) were the two most populated states. Each amino acid was assigned a secondary structure code, i.e., E: extended strand, S: bend, T: turn, and C: coil. The length of the β-strand is estimated by summing the occurrences of the extended strands (E) along the sequence. The longer β-strands in the CRD loops are favored between $d_z = 0$ and $d_z = 4$ nm, while shorter β-strands are favored between $d_z = 4$ nm and $d_z = 8$ nm. The two major populations are β-strands of length 18 and 9, with minor populations of length 17, 16, 8, and 5. The data is shown on linear (left) and log scales (right).
