# Supplementary material for: Membrane lipids drive formation of KRAS4b-RAF1 RBDCRD nanoclusters on the membrane
Source: Commun Biol. 2024 Feb 28;7:242. doi: 10.1038/s42003-024-05916-0 (PMC10902389; doi:10.1038/s42003-024-05916-0)
Supplement: Supplementary file 3 — Reporting Summary [file 42003_2024_5916_MOESM3_ESM.pdf]

nature portfolio

Corresponding author(s): Thomas J. Turbyville

Last updated by author(s): Jan 22, 2024

Reporting Summary

Nature Portfolio wishes to improve the reproducibility of the work that we publish. This form provides structure for consistency and transparency in reporting. For further information on Nature Portfolio policies, see our Editorial Policies and the Editorial Policy Checklist.

Statistics

For all statistical analyses, confirm that the following items are present in the figure legend, table legend, main text, or Methods section.

n/a	Confirmed	
☐	☒	The exact sample size (*n*) for each experimental group/condition, given as a discrete number and unit of measurement
☐	☒	A statement on whether measurements were taken from distinct samples or whether the same sample was measured repeatedly
☒	☐	The statistical test(s) used AND whether they are one- or two-sided<br>*Only common tests should be described solely by name; describe more complex techniques in the Methods section.*
☒	☐	A description of all covariates tested
☒	☐	A description of any assumptions or corrections, such as tests of normality and adjustment for multiple comparisons
☐	☒	A full description of the statistical parameters including central tendency (e.g. means) or other basic estimates (e.g. regression coefficient) AND variation (e.g. standard deviation) or associated estimates of uncertainty (e.g. confidence intervals)
☒	☐	For null hypothesis testing, the test statistic (e.g. *F*, *t*, *r*) with confidence intervals, effect sizes, degrees of freedom and *P* value noted<br>*Give P values as exact values whenever suitable.*
☒	☐	For Bayesian analysis, information on the choice of priors and Markov chain Monte Carlo settings
☒	☐	For hierarchical and complex designs, identification of the appropriate level for tests and full reporting of outcomes
☒	☐	Estimates of effect sizes (e.g. Cohen's *d*, Pearson's *r*), indicating how they were calculated

Our web collection on statistics for biologists contains articles on many of the points above.

Software and code

Policy information about availability of computer code

Data collection	Single particle tracking movies were acquired using NIS Element AR 5.21.03 software. NMR data were collected on an Agilent 800 MHz and Bruker Avance II 500, 600, and 900 MHz spectrometer. Simulation campaign was performed using MuMMI. MuMMI consists of many sub-components, both freely available third-party applications and custom codes: - ddcMD (github.com/LLNL/ddcMD), - ddcMDconvertergithub.com/LLNL/ddcMDconverter), - MDAnalysis modified to support ddcMD (github.com/XiaohuaZhangLLNL/mdanalysis), - Maestro (github.com/LLNL/maestrowf), - Flux (github.com/flux-framework), - Pytaridx (github.com/LLNL/pytaridx), - dynIm (github.com/LLNL/dynim), - MuMMIcore (github.com/mummi-framework/mummi-core), and MuMMI-ras (github.com/mummi-framework/mummi-ras). In addition, external software such as Gromacs (https://www.gromacs.org/) and Amber (https://ambermd.org/) were used.
Data analysis	Single particle tracking experiments were analysed using the Localizer plugin in Igor Pro. The mean square displacement plots were obtained using a home written matlab code. The HMM analysis were perfomed uisng the vbSPT software package. NMR data were processed using NMRPipe and anayzed using CcpNMR and POKY. Simulations were analyzed using MuMMI, MDAnalysis (https://www.mdanalysis.org/), VMD (https://www.ks.uiuc.edu/Research/vmd/) and Biopython (https://biopython.org/).

For manuscripts utilizing custom algorithms or software that are central to the research but not yet described in published literature, software must be made available to editors and reviewers. We strongly encourage code deposition in a community repository (e.g. GitHub). See the Nature Portfolio guidelines for submitting code & software for further information.

Data

Policy information about availability of data

All manuscripts must include a data availability statement. This statement should provide the following information, where applicable:

- Accession codes, unique identifiers, or web links for publicly available datasets
- A description of any restrictions on data availability
- For clinical datasets or third party data, please ensure that the statement adheres to our policy

The raw data for each MSD plots are provided in the supplemental information. The raw single particle tracking movies are available on request. NMR spectrum and peak assignments are provided in the supplemental section. Raw NMR-PRE spectra are available on request. All simulation parameter and input files will be made accessible at https://bbs.llnl.gov/. All simulation raw data will be hosted on the NIH MoDaC server (https://modac.cancer.gov/).

Research involving human participants, their data, or biological material

Policy information about studies with human participants or human data. See also policy information about sex, gender (identity/presentation), and sexual orientation and race, ethnicity and racism.

Reporting on sex and gender	*Use the terms sex (biological attribute) and gender (shaped by social and cultural circumstances) carefully in order to avoid confusing both terms. Indicate if findings apply to only one sex or gender; describe whether sex and gender were considered in study design; whether sex and/or gender was determined based on self-reporting or assigned and methods used. Provide in the source data disaggregated sex and gender data, where this information has been collected, and if consent has been obtained for sharing of individual-level data; provide overall numbers in this Reporting Summary. Please state if this information has not been collected. Report sex- and gender-based analyses where performed, justify reasons for lack of sex- and gender-based analysis.*
Reporting on race, ethnicity, or other socially relevant groupings	*Please specify the socially constructed or socially relevant categorization variable(s) used in your manuscript and explain why they were used. Please note that such variables should not be used as proxies for other socially constructed/relevant variables (for example, race or ethnicity should not be used as a proxy for socioeconomic status). Provide clear definitions of the relevant terms used, how they were provided (by the participants/respondents, the researchers, or third parties), and the method(s) used to classify people into the different categories (e.g. self-report, census or administrative data, social media data, etc.) Please provide details about how you controlled for confounding variables in your analyses.*
Population characteristics	*Describe the covariate-relevant population characteristics of the human research participants (e.g. age, genotypic information, past and current diagnosis and treatment categories). If you filled out the behavioural & social sciences study design questions and have nothing to add here, write "See above."*
Recruitment	*Describe how participants were recruited. Outline any potential self-selection bias or other biases that may be present and how these are likely to impact results.*
Ethics oversight	*Identify the organization(s) that approved the study protocol.*

Note that full information on the approval of the study protocol must also be provided in the manuscript.

Field-specific reporting

Please select the one below that is the best fit for your research. If you are not sure, read the appropriate sections before making your selection.

☒ Life sciences ☐ Behavioural & social sciences ☐ Ecological, evolutionary & environmental sciences

For a reference copy of the document with all sections, see nature.com/documents/nr-reporting-summary-flat.pdf

Life sciences study design

All studies must disclose on these points even when the disclosure is negative.

Sample size	At least 15 movies of 5000 frames each were collected per tracking experiments. At least 2 repeats of each experimental conditions were performed. Only one sample per NMR-PRE experiment was used.
Data exclusions	No data were excluded.
Replication	At least 2 repeats of each experimental conditions were performed for single particle tracking studies.
Randomization	N/A
Blinding	N/A

Reporting for specific materials, systems and methods

We require information from authors about some types of materials, experimental systems and methods used in many studies. Here, indicate whether each material, system or method listed is relevant to your study. If you are not sure if a list item applies to your research, read the appropriate section before selecting a response.

Materials & experimental systems

n/a	Involved in the study
☒	☐ Antibodies
☒	☐ Eukaryotic cell lines
☒	☐ Palaeontology and archaeology
☒	☐ Animals and other organisms
☒	☐ Clinical data
☒	☐ Dual use research of concern
☒	☐ Plants

Methods

n/a	Involved in the study
☒	☐ ChIP-seq
☒	☐ Flow cytometry
☒	☐ MRI-based neuroimaging
