# Supplementary material for: Membrane lipids drive formation of KRAS4b-RAF1 RBDCRD nanoclusters on the membrane
Source: Commun Biol. 2024 Feb 28;7:242. doi: 10.1038/s42003-024-05916-0 (PMC10902389; doi:10.1038/s42003-024-05916-0)
Supplement: Supplementary file 1 — Peer Review File [file 42003_2024_5916_MOESM1_ESM.pdf]

nature portfolio

Peer Review File

Membrane lipids drive formation of KRAS4b-RAF1 RBDCRD nanoclusters on the membrane

Open Access This file is licensed under a Creative Commons Attribution 4.0 International License, which permits use, sharing, adaptation, distribution and reproduction in any medium or format, as long as you give appropriate credit to the original author(s) and the source, provide a link to the Creative Commons license, and indicate if changes were made. In the cases where the authors are anonymous, such as is the case for the reports of anonymous peer reviewers, author attribution should be to 'Anonymous Referee' followed by a clear attribution to the source work. The images or other third party material in this file are included in the article's Creative Commons license, unless indicated otherwise in a credit line to the material. If material is not included in the article's Creative Commons license and your intended use is not permitted by statutory regulation or exceeds the permitted use, you will need to obtain permission directly from the copyright holder. To view a copy of this license, visit http://creativecommons.org/licenses/by/4.0/.

Reviewers' comments:

Reviewer #1 (Remarks to the Author):

The manuscript by Shrestha et al. investigates the cooperative interaction and clustering of KRAS and RAF1 RBDCRD at the membrane by single particle tracking, nuclear magnetic resonance spectroscopy, and multiscale simulation techniques. This paper reports new information on the complex interplay between the proteins and bilayers with different lipid compositions, which is important for understanding the nanoclustering and cell signaling. While this study is of interest to readers in this field, the following questions and comments need to be addressed:

The authors used a logarithmic curve, obtained by fitting of the simulation data for KRAS-only clusters, for estimating the experimental cluster size of KRAS-RBDCRD. However, as it has been observed that RBDCRD binding can significantly slow down the diffusion, wouldn't this calculation introduce systematic errors?

The simulation data for KRAS-RBDCRD are also reported in the manuscript, which in principle are better suited for deriving such a curve. However, they do not appear to follow a logarithmic decay (Fig. 7B), which brings into question the reliability of the simulation data and estimated cluster sizes.

p23. the expression "... the center of mass (COM) of a Cα atom of a residue ..." should be revised, as only one atom is considered here.

p.24 The description of the contact probability as a function of dz is puzzling in this section. The definition of dz indicates that smaller values correspond to deeper insertion, opposite to how it is explained for Figure 8.

Reviewer #3 (Remarks to the Author):

The manuscript under consideration explores the molecular interactions between KRAS and RAF in the context of membrane reconstitution, shedding light on spatiotemporal organization of the RAS-RAF signaling complex at the membrane. The study employs a combination of experimental techniques, such as single particle tracking and NMR PRE experiments, along with multiscale molecular dynamics simulations, to investigate the dynamics and interactions of KRAS and RAF at the membrane. The work expands on previous findings by this group employing MuMMI to model KRAS4b diffusion and conformational dynamics associated with more complex model membranes to correlate distinct local lipid microenvironments and RAS clustering at the membrane. While considerable progress has been made in structural elucidation of RAF complexes, most studies have been conducted in the absence of biological membranes. The work herein focuses on RAF RBD-CRD interactions with membranes of differing composition in the absence and presence of RAS, which adds an additional level of complexity. As such, the findings provide new insights into the role of membrane composition, interactions, clustering and spatial/temporal distribution that provide insight into understanding how RAS-mediates RAF activation at the membrane. However, some concerns exist with the lack of 1) errors associated with some of the figures, 2) methodological details, 3) molecular models and 4) detailed description of how the work expands on previous findings. Detailed comments are listed below.

1. In Figure 1 (and S1), the dimension of sections used is not denoted.
2. The authors should show the NMR HSQC overlay data (CSP and broadening) in the main text, and map changes onto the structure of the RBD-CRD complex. The PRE raw data and errors associated with intensity changes are not shown.

3. MSD data associated with Figures 3, 4 and 5 should be consolidated.
4. In Figure 5, to test if KRAS dimer formation alters diffusion, BI2852 was used to form a non-productive KRAS dimer. Was RAS dimerization confirmed under the conditions studied? Some discussion regarding how the 8-lipid system shows more confinement over the 2-lipid system is needed.
5. Given that the experimental temperature given is 35 °C, and the temperature for backbone assignments is 25 °C, the authors should clarify how assignments were obtained.
6. Fig 10 legend ..” wild type RAF1 RBDCRD (color) and…” The authors should replace the word color with solid and dotted for the mutant.
7. Fig S6 legend should read linear (left) and log scales (right).
8. The method of nucleotide loading and loading verification is not described.
9. NMR backbone resonance methods and assignments are not listed/described.
10. The methods for the PRE experiments are not fully described.
11. The CRD interaction residues and mutants listed in the manuscript closely resemble those already reported in a previously published work (DOI: https://doi.org/10.1038/s41467-021-21422-x). The authors should highlight any novel insights gained from the additional experiments or analyses presented in this study. Furthermore, while the manuscript discusses the role of membrane composition in regulating RAS-RAF interactions and nanocluster formation, it could benefit from a deeper comparison and integration of its results with the recent publication's insights regarding the selective interaction of RAF1(CRD) with phosphatidylserine (PS) and the detailed mapping of CRD residues involved in membrane interactions.

12. The clustering methodology described appears to be a simplified approach to identifying protein clusters in comparison to more advanced clustering algorithms such as density-based or machine learning approaches. The methodology employed involves selecting a protein point and then assigning neighboring points within a specified distance to the same cluster label. While simplicity may be required/advantageous in complex biological systems, it is unclear whether the methodology provides a robust statistical or analytical basis. For example, it is unclear how additional factors such as density, distribution, or the actual nature of protein-protein interactions influence formation of clusters.

13. The authors performed the conversion of coarse-grained (CG) frames to all-atom (AA) and executed AA simulations using CHARMM36 ff. However, specifics about the selection criteria for this conversion are lacking. The manuscript should provide clear details about the specific criteria, such as a) structurally stable frames, b) frames with diverse protein orientations with different CRD membrane insertions, and c) frames with significant protein-membrane interactions.

14. Given that the membrane composition can vary in different cellular contexts, some discussion on how changes in biologically-relevant lipid composition influence nanocluster formation and the membrane association/activity of the KRAS-RAF complex should be discussed.

15. The MSD plots lack error bars. While the supplemental information contains a data table, the omission of errors on the figures limits the quantifiable assessment of diffusion differences between the wild type and mutant KRAS-RBDCRD complexes.

16. The author's correlation between β-strand length and the depth of CRD loops in the membrane (Fig. 11a) raises concerns. The reported data appears to lack a significant difference (statistically) in β-strand lengths between various depths.

17. Fig. 11C illustrates potential orientations of the KRAS-RBDCRD complex at varying dz values. The authors should provide more detailed information about the interaction network and the criteria for identifying CRD orientation at distinct dz values.

18. In the discussion section, authors propose a model to describe the RAS-RAF interaction at the membrane, yet molecular models of membrane associated RAS-RAF complexes and pictorial

representation(s) of the proposed model are lacking.

19. The manuscript would benefit from a more detailed description that highlights novelty of the findings associated with 1) membrane composition playing a role in RAS/RAF diffusion mechanisms and clustering, 2) how simpler artificial membranes promote Brownian diffusion and more complex membranes exhibit a hierarchical diffusion process, and the 3) use of individual and combined RBD and CRD domains to tease out the RAF1 contributions to membrane association.

Reviewer #4 (Remarks to the Author):

This manuscript describes results from an interesting piece of work combining single-molecule tracking, NMR, and computational analyses of KRAS-RAFRBDCRD interactions in supported and model membranes of complex lipid compositions. Consistent with previous reports by the same group and others, KRAS was found to diffuse at three different rates, suggesting multiple multimeric (and monomeric) forms. This is true for all lipid compositions tested, including binary PC/PS and PC/PIP2 membranes or more complex membranes made up of 6, 7 and 8 lipid species. In the most complex 8-component supported membrane, the dominant form (72%) diffuses at an intermediate rate whereas the fast-diffusing species represent only 11% of the total. These distributions changed when RAF-RBDCRD is present, with the intermediate and slow diffusing species now being about equally populated (45 and 47%). This is interpreted as arising from the formation of KRAS-RBDCRD nanoclusters. Using NMR, the authors also show that a number of hydrophobic and basic residues of the CRD engage membrane lipids, which is supported by data from atomistic molecular dynamics (MD) simulations. CRD-membrane interaction is not a particularly new finding but still useful. Overall, this is an important contribution toward elucidating the organizing principles of RAS/effector complexes on membrane surfaces. The manuscript is well written and, for the most part, the conclusions are consistent with the data. However, there are some issues that require clarification.

1) Although not directly stated by the authors, it is remarkable that diffusion rates change little with lipid composition whereas the proportion of the fast, intermediate, and slow-diffusing species changes dramatically (Table S2). This is true irrespective of KRAS being alone or interacting with RBD or RBDCRD. It would seem, therefore, that clustering is an inherent property of KRAS molecules, with the different lipids helping tip the balance between populations. For example, in the 8-lipids system the fraction of the fast-diffusing species decreased by up to 10-fold whereas those of the slow and intermediate fractions increased by a similar amount. The other lipid mixtures have also altered the distributions albeit to a lesser extent. Would the authors comment on this and/or discuss their data in more detail?

2) Related to the above, it is clear from Fig 4 that the significance of the 8-lipid system lies in increasing confinement. This system differs from the 7-component system by the presence of cholesterol, suggesting that cholesterol is critical for confinement. The authors acknowledge this: "KRAS also randomly diffused in the cholesterol depleted 6-lipid and 7-lipid bilayers" but do not elaborate and explain. What does cholesterol do to confine KRAS? Would it make the bilayer rigid (33% of chol is significant)? How does this relate to the well-established fact that KRAS prefers cholesterol depleted, liquid disordered domains? Also, recent studies suggest that chol is mostly located in the outer leaflet of the plasma membrane, whereas KRAS is in the inner leaflet.

3) The authors state (and have shown previously; ref 17) that their 8-lipid system recapitulates diffusion rates measured in cells. Could this be fortuitous, because confined diffusion in cells is likely due to actin and other membrane components than cholesterol, at least for KRAS. It would be nice if the authors shade light on this issue. This is particularly important now that they show it is cholesterol, not just the complexity of the membrane, that plays a critical in yielding a more confined diffusion (per Fig 4).

4) The authors state “One hypothesis is that upon RBDCRD binding, KRAS-RBDRCRD complexes instantaneously assemble into clusters with slow diffusion” (page 15). As noted in 1 above, it would seem from the data that KRAS itself is able to instantaneously form clusters, so that RBDCRD binding decreases the overall diffusion rate by increasing the population of the slower species rather than driving cluster formation. Would the authors comment on this?

5) The significance of most of the simulations data is unclear (at least to this reviewer). In particular, the data from continuum modeling (Fig 6) does not seem to add much to the discussion, and the diffusion rates from the CG simulations shown in Fig 7 are problematic in many respects. First, although understandable from a technical standpoint, the average diffusion rates from the simulations are about 10-fold larger than that experimentally measured for the dominant species (the extrapolation in Fig 8 shows that the simulation failed to sample larger clusters). Secondly, it is not clear what fraction of molecules in the simulation box or for what fraction of time have “clustered”. The numbers quoted for KRAS-only 4-mers “~30, compared with >3000” suggest a very small (<<1%) fraction of clusters compared to total. This is concerning, and raises the question of whether or not true cluster formation has occurred as compared to random clashes. Thirdly, it appears that the 1-mer, 2-mer and 3-mer KRAS-RBDRDs diffuse with comparable rates and slightly faster than the 4-mer, 5-mer and 6-mer clusters that diffuse at a comparable rate (Fig 7B). This is confusing. Is there any reason for the existence of these two classes despite their different sizes? The same could be said about Fig 7A.

Dear Reviewers, December 21, 2023

We sincerely value the valuable feedback you shared regarding our manuscript, titled "Investigating the Cooperative Interaction and Clustering of KRAS and RAF1 RBDCRD at the Membrane." We have conscientiously incorporated your suggestions, and we believe that your insights have greatly contributed to improving the quality and clarity of the manuscript. Kindly find our responses to your comments inline below.

Reviewer #1 (Remarks to the Author):

The manuscript by Shrestha et al. investigates the cooperative interaction and clustering of KRAS and RAF1 RBDCRD at the membrane by single particle tracking, nuclear magnetic resonance spectroscopy, and multiscale simulation techniques. This paper reports new information on the complex interplay between the proteins and bilayers with different lipid compositions, which is important for understanding the nanoclustering and cell signaling. While this study is of interest to readers in this field, the following questions and comments need to be addressed:

1. The authors used a logarithmic curve, obtained by fitting of the simulation data for KRAS-only clusters, for estimating the experimental cluster size of KRAS-RBDCRD. However, as it has been observed that RBDCRD binding can significantly slow down the diffusion, wouldn't this calculation introduce systematic errors? The simulation data for KRAS-RBDCRD are also reported in the manuscript, which in principle are better suited for deriving such a curve. However, they do not appear to follow a logarithmic decay (Fig. 7B), which brings into question the reliability of the simulation data and estimated cluster sizes.

We thank the reviewer for the comment. The reviewer makes good points regarding the fitting of data to logarithmic decay curves. As such, this section has been almost entirely removed and a condensed, truncated version placed into the supporting document under Figure S5.

Only the following text remains in the main manuscript:

"The KRAS diffusion data fits to a decay curve (Figure S5), as is the expected correlation between protein size and membrane diffusion [58]" (line 665-666)

We have also noted that we attempted a similar process using the KRAS-RBDCRD diffusion, however, the data does not really allow for such a prediction as the values do not decrease enough to fit to an exponential decay. There are several reasons for this:

i) Diffusion calculations from simulation data are often extremely noisy (as seen in the violin plots of Figure7) and can be system-dependent (indeed, being

able to obtain data from the KRAS simulations that is even close to fitting the expected decay trends is quite a challenge)

ii) There is much less sampling of these KRAS-RBDCRD systems.

iii) Longer relaxation times are needed for a big cluster – further compounding the issues in points (i and ii)

iv) The precise metric of how to measure diffusion is complicated – often the center of mass of the protein is chosen and the movement of that coordinate is tracked. However, with so many domains and proteins all in motion, any diffusion measurement become *even more* noisy and muddled. Thus, the increased internal dynamics of the larger clusters (i.e. one KRAS-RBDCRD moving around while still being bound to the others) may likely inflate the diffusion value.

To summarize the above points, the following sections of text have been added:

> “Despite diffusion calculations from simulation data often being extremely noisy and variable…” (line 660)
>
> “KRAS-RBDCRD diffusion did not allow for such fitting – likely due to both insufficient sampling of the simulations and additional complexities associated with diffusion calculation of the more intricate, multi-domain systems.” (line 666-669)

Additionally, it is unclear how to best combine the experimental and computational KRAS-RBDCRD values as we do not know the precise fraction of the KRAS population that are bound to RBDCRD in the experiments.

In the text of the paper, we have emphasized the issues and caveats with the computational diffusion analysis and mentioned that that KRAS-RBDCRD data does not work for this approach.

2. p23. the expression “... the center of mass (COM) of a Cα atom of a residue ...” should be revised, as only one atom is considered here.
We thank the reviewer for pointing this out. We have revised the sentence as follows:

“if the distance between Cα atom of a residue and P atom in the lipid headgroup is less than or equal to 0.85 nm.” (line 729)

3. p.24 The description of the contact probability as a function of dz is puzzling in this section. The definition of dz indicates that smaller values correspond to deeper insertion, opposite to how it is explained for Figure 8.

We thank the reviewer for bringing this to our attention. We have revised the explanation for Figure 8 in Section 3.4 (line 744-762) as follows:

“Figure 8C shows the contact probability calculated for each residue at different depths across the lipid bilayer. The contacts calculated between 0-2 nm align with the NMR PRE experiments performed with a gadolinium spin label on the lipid headgroup; 2-4 nm align with doxyl-5 lipids and 4-6 nm correspond to the doxyl-15 lipids. We found the CRD contact probabilities get lower with longer distances between the COM of hydrophobic/cationic CRD loop residues and the COM of the bilayer along the bilayer normal (dz). The comparison between changes in intensities from NMR measurements and contact probabilities shows consistent results, especially in the highest intensity change/contact probability regions. For example, within 0-2 nm insertion depths, both NMR and AA simulation identify high contacts between the residues along the two flexible CRD residues (144 to 150, 158, 159 and 176). At longer distances between the CRD and the bilayer (2-4 nm), there is overlap between residues 149, 159 and 176 whereas there is the least overlap at the largest distances (4-6 nm and 6-8 nm). This discrepancy may be due to the absence of KRAS in the NMR-PRE experiments and implies that the binding of KRAS decreases the distance between the CRD loops and the bilayer. Additionally, our NMR experiments use bicelles composed of 2-lipids, whereas the simulation studies use the more complex 8-lipid system, which alters the lipid acyl chain packing and may also contribute to these differences and hence greater variation.’

Reviewer #3 (Remarks to the Author):

The manuscript under consideration explores the molecular interactions between KRAS and RAF in the context of membrane reconstitution, shedding light on spatiotemporal organization of the RAS-RAF signaling complex at the membrane. The study employs a combination of experimental techniques, such as single particle tracking and NMR PRE experiments, along with multiscale molecular dynamics simulations, to investigate the dynamics and interactions of KRAS and RAF at the membrane. The work expands on previous findings by this group employing MuMMI to model KRAS4b diffusion and conformational dynamics associated with more complex model membranes to correlate distinct local lipid microenvironments and RAS clustering at the membrane. While considerable progress has been made in structural elucidation of RAF complexes, most studies have been conducted in the absence of biological membranes. The work herein focuses on RAF RBD-CRD interactions with membranes of differing composition in the absence and presence of RAS, which adds an additional level of complexity. As such, the findings provide new insights into the role of membrane composition, interactions, clustering and spatial/temporal distribution that provide insight into understanding how RAS-mediates RAF activation at the membrane. However, some concerns exist with the lack of 1) errors associated with some of the figures, 2) methodological details, 3) molecular models and 4) detailed description of how the work expands on previous

findings. Detailed comments are listed below.

1. In Figure 1 (and S1), the dimension of sections used is not denoted.

We thank the reviewer for pointing this out. We added the dimensions of the region of interest (ROI) "*(150 pixel x 150 pixel)"* in the figure legends of Figure 1 and Figure S1.

2. The authors should show the NMR HSQC overlay data (CSP and broadening) in the main text, and map changes onto the structure of the RBD-CRD complex. The PRE raw data and errors associated with intensity changes are not shown.

We thank the reviewer for the comment. A spectral overlay of RBDCRD bound to DMPC/DMPS bicelles containing doxyl-5, doxyl-14 and Gd^{3+} has been added, Figure 8B. NMR-PRE ratios of ≤50% were mapped onto a model of RBDCRD shown in Figure 8D.

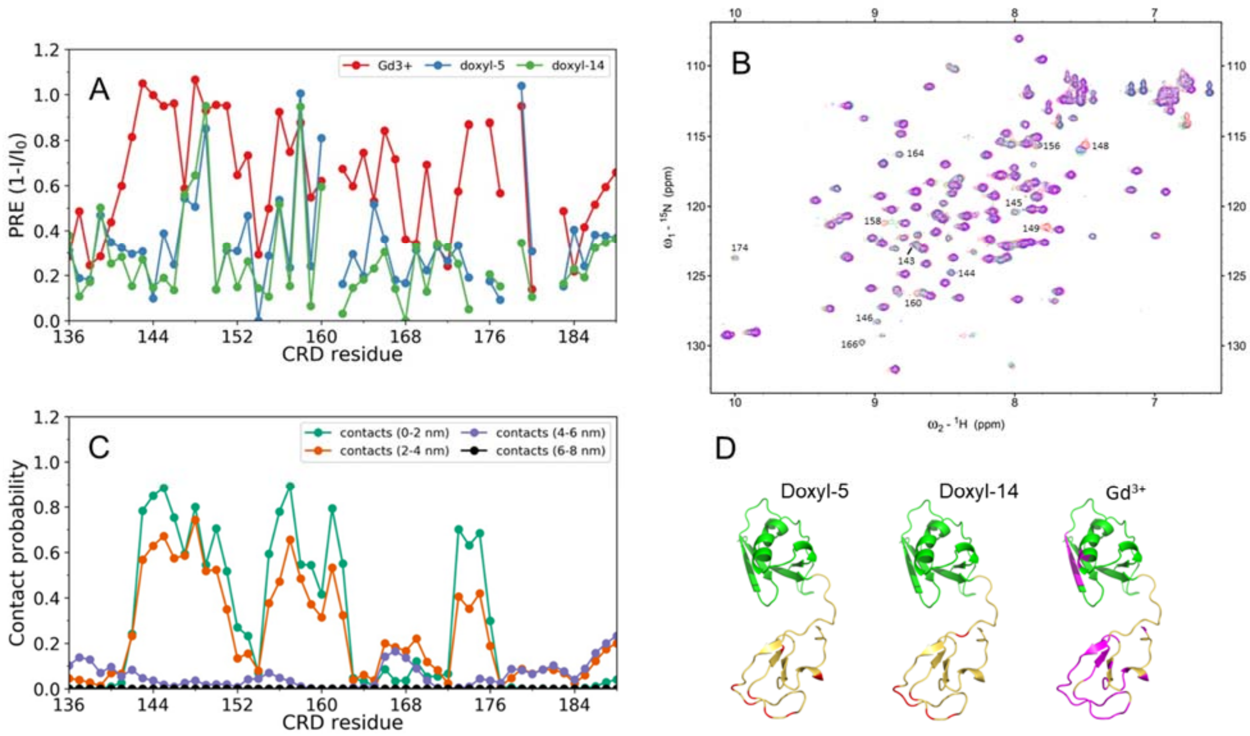

Figure 8. (A) Dot plot representation of NMR-PRE ratios (I/I_0) plotted as (1-(I/I_0) to have a clearer comparison with contact probabilities obtained from the MuMMI AA simulations. (B) HSQC spectra showing reduction in intensities of CRD residues of RBDCRD bound to DMPC:DMPS bicelles (red) upon the addition of doxyl-5 (blue), doxyl-14 (green) and Gd^{3+} (magenta). (C) Contact probability distribution of CRD residues in KRAS-RBDCRD as a function of the distance between the COM of hydrophobic/cationic CRD loop residues and the COM of the bilayer along the bilayer

normal, obtained from MuMMI AA simulations. (D) CRD residues with PRE ratios of ≤50% are mapped onto a model of RBDCRD: doxyl-5 and doxyl-14 (red) and Gd^{3+} (magenta).

All PRE ratios calculated from peak intensities are shown in Figure S8. The errors for the PRE ratios are shown in Figure S8. Residues with no assignments, overlapping peaks, and other peaks excluded from the final PRE analysis are listed in the methods section.

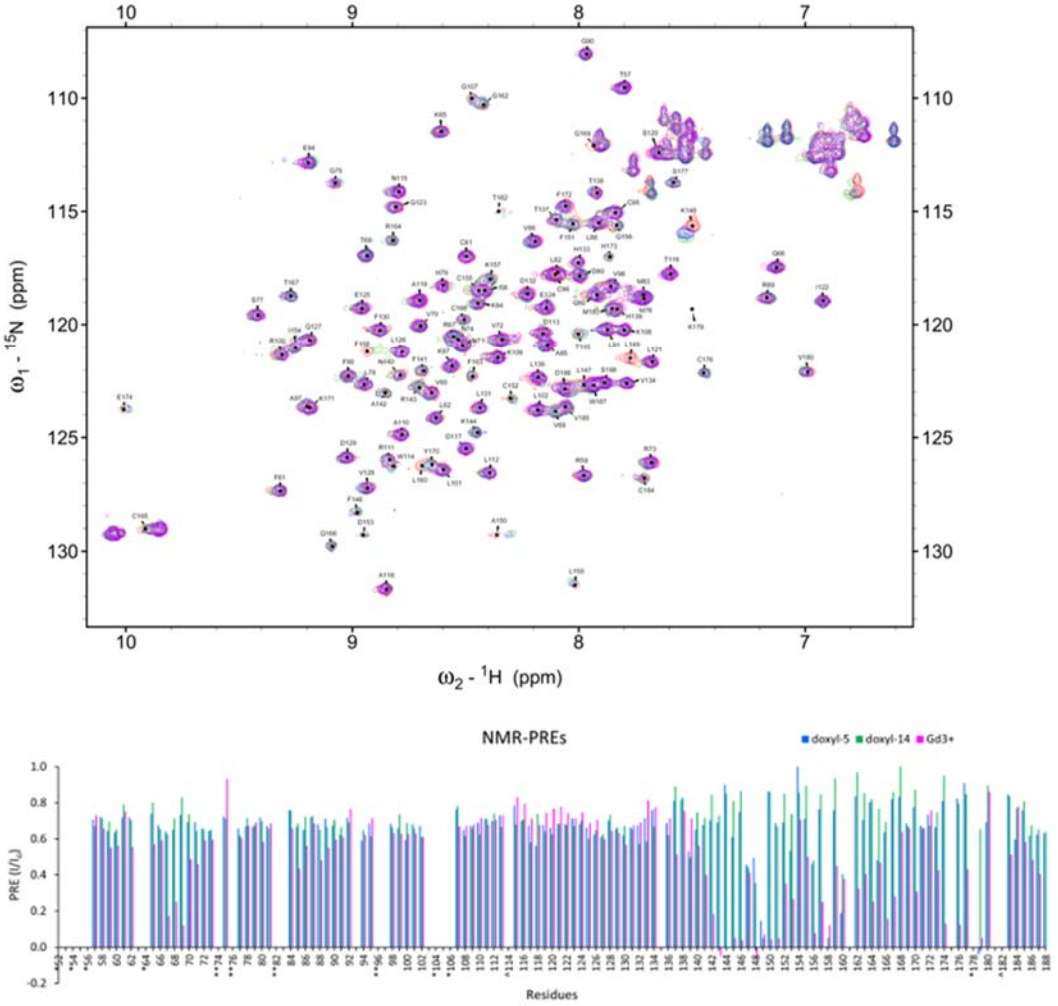

Figure S8. Spectral overlay of RBDCRD bound to bicelles with doxyl-5, doxyl-14, and Gd^{3+} (top). Normalized PRE ratios for RBDCRD on bicelles with doxyl-5, doxyl-14 and Gd^{3+} showing reduced PRE ratios for RBD residues 67, 68, 69 along with the majority of CRD residues (bottom). Residues with no assignments are marked with a single asterisk (*), and excluded overlapped peaks are marked with two asterisks (**).

3. MSD data associated with Figures 3, 4 and 5 should be consolidated.

We thank the reviewer for the comment. The figures were organized in the original submission to describe the results from each corresponding section. Therefore, we feel that the current placement of the figures provides readers with a better flow and will make it easier to follow the manuscript.

4. In Figure 5, to test if KRAS dimer formation alters diffusion, BI2852 was used to form a non-productive KRAS dimer. Was RAS dimerization confirmed under the conditions studied? Some discussion regarding how the 8-lipid system shows more confinement over the 2-lipid system is needed.

We thank the reviewer for the comment. A previous publication from our colleagues showed that BI2852 compound formed a KRAS dimer with rotational symmetry, as revealed in the published crystal structure. They confirmed this using a variety of biophysical techniques such as size exclusion chromatography, native mass spectrometry, isothermal titration calorimetry, and surface plasmon resonance. Based on this extensive and thorough analysis, we presumed that BI2852 also formed dimers in our experiments. We added the detail of the publication in Section 3.3.1 (line 556-564) in the main manuscript.

5. Given that the experimental temperature given is 35 °C, and the temperature for backbone assignments is 25 °C, the authors should clarify how assignments were obtained.

We thank the reviewer for bringing this to our attention. This was an inadvertent typo error in the manuscript. The experimental temperature was indeed performed at 25 °C. This has been corrected in the manuscript. We would also like to mention that the original CSP plot shown for free RBDCRD versus RBDCRD bound to bicelles (original Figure S5) was mistakenly generated using just changes in 1H chemical shifts. This plot has been replaced with one generated using the equation listed in the methods section in the new Figure S6A and B.

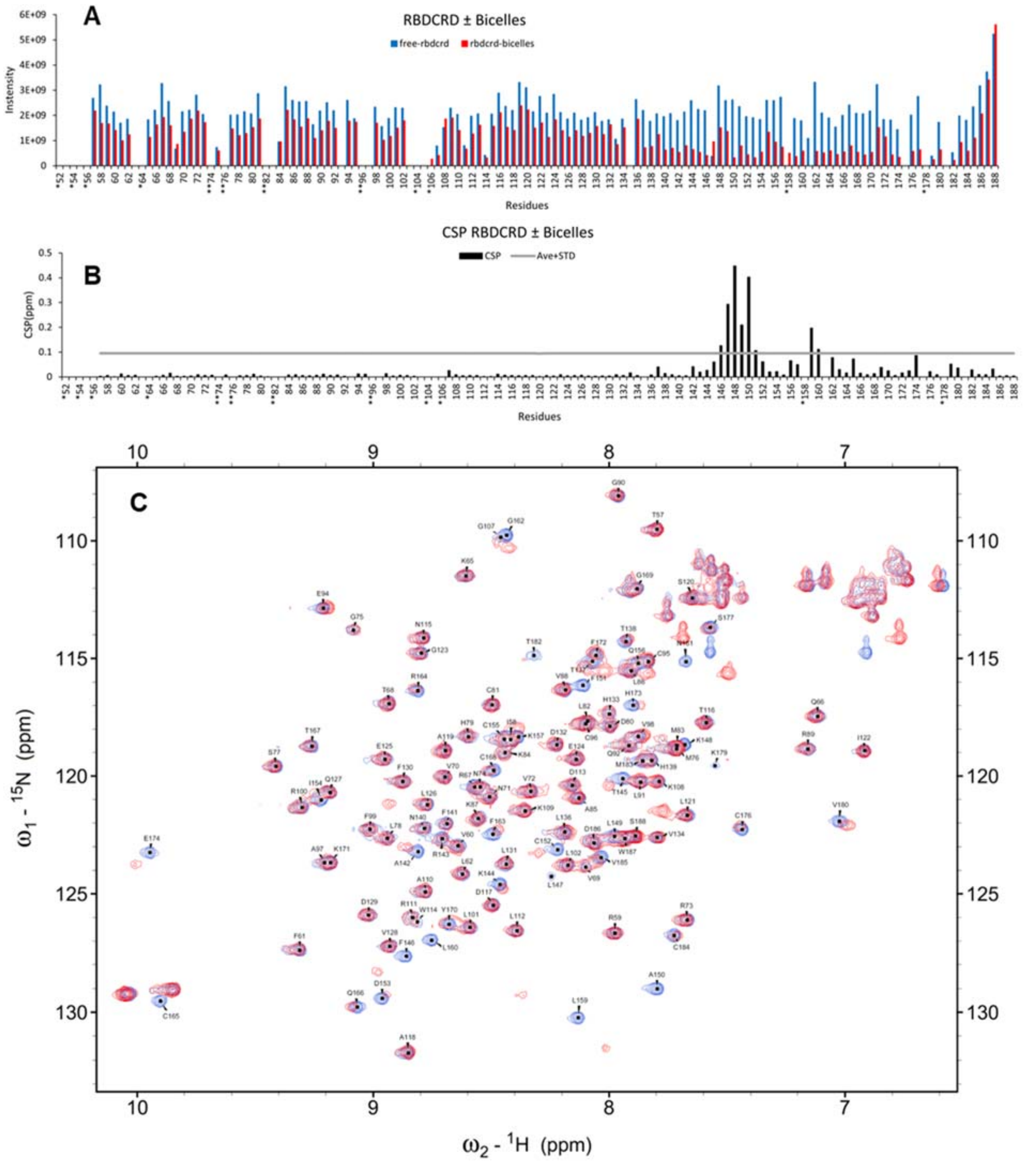

Figure S6.The peak intensities (A) and chemical shift perturbations (B) of RAF1 RBDCRD alone vs RBDCRD bound to 70:30 DMPC:DMPS bicelles. (C) The HSQC spectrum of free RBDCRD (blue) with amide backbone assignments and the spectrum of RBDCRD bound to bicelles (red). Residues with no assignments are marked with a single asterisk (*), and excluded overlapped peaks are marked with two asterisks (**).

6. Fig 10 legend ..” wild type RAF1 RBDCRD (color) and…” The authors should replace the word color with solid and dotted for the mutant.

We thank the reviewer for the comment. The figure legend was edited as follows:

“Figure9. *Mean square displacement plots of KRAS4b on 8-lipid bilayer when bound to wild type RAF1 RBDCRD (solid) and mutated RAF1 RBDCRD K148A/K157A/K179A (dotted)*.”

7. Fig S6 legend should read linear (left) and log scales (right).

We thank the reviewer for the comment. The figure legend in Figure S9 (previously Figure S6) is corrected as suggested.

“Figure S9. A clustering approach was employed on the total lengths of the β-strand in the loop 1 and 2 of the CRD region with bin size of 2 nm. The cluster analysis identified 6 different populations of which β-strand length of 18 (11E-7E: EEEEEEEEEEECTTTCSEEEEEEE) and length of 9 (5E-4E: EEEEECCSCCCCTTTCSCCSEEEE) were the two most populated states. Each amino acid was assigned a secondary structure code, i.e., E: extended strand, S: bend, T: turn, and C: coil. The length of the β-strand is estimated by summing the occurrences of the extended strands (E) along the sequence. The longer β-strands in the CRD loops are favored between $d_z = 0$ and $d_z = 4$ nm, while shorter β-strands are favored between $d_z = 4$ nm and $d_z = 8$ nm. The two major populations are β-strands of length 18 and 9, with minor populations of length 17, 16, 8, and 5. The data is shown on linear (left) and log scales (right).”

8. The method of nucleotide loading and loading verification is not described.

We thank the reviewer for bringing this to our attention. The nucleotide loading of purified loading was performed according to a previously published protocol (reference #17 and 32) and verified using HPLC technique. The reference has been added to the Methods and Material section.

9. NMR backbone resonance methods and assignments are not listed/described.

We thank the reviewer for the comments, we have added more information on the backbone assignments in the methods section (line 266-275). The backbone assignments for RBDCRD alone in solution and all RBDCRD bound to bicelles (Figure S6C) have been added to the supplemental section. (see response to comment# 5)

“NMR data were collected on an Agilent 800 MHz and Bruker Avance II 500, 600, and 900 MHz spectrometer at 25 ^{o}C, processed with NMRPipe [34] and analyzed using CcpNMR[35]. The backbone resonances of RBDCRD(52-188) with and without bicelles were assigned using standard triple resonance data (HNCA, HNCACB, CBCACONH and ^{15}N-edited HSQC-NOESY). Data were collected at 25 ^{o}C on 300 mM samples in a buffer containing 20 mM HEPES (pH 6.5), 300 mM NaCl, 50 mM Glutamate, 50 mM Arginine, and 1 mM TCEP-HCl. Residues 52-56, 64, 103-105, 158, 175, 178 and four prolines (63, 93, 135, 181) had no assignments. A strong peak for 161 was observed in free RBDCRD but was not detectable in the presence of bicelles. Conversely, 158 was only observed for samples with bicelles. Amide backbone assignments are shown in Figure S6 and S7.”

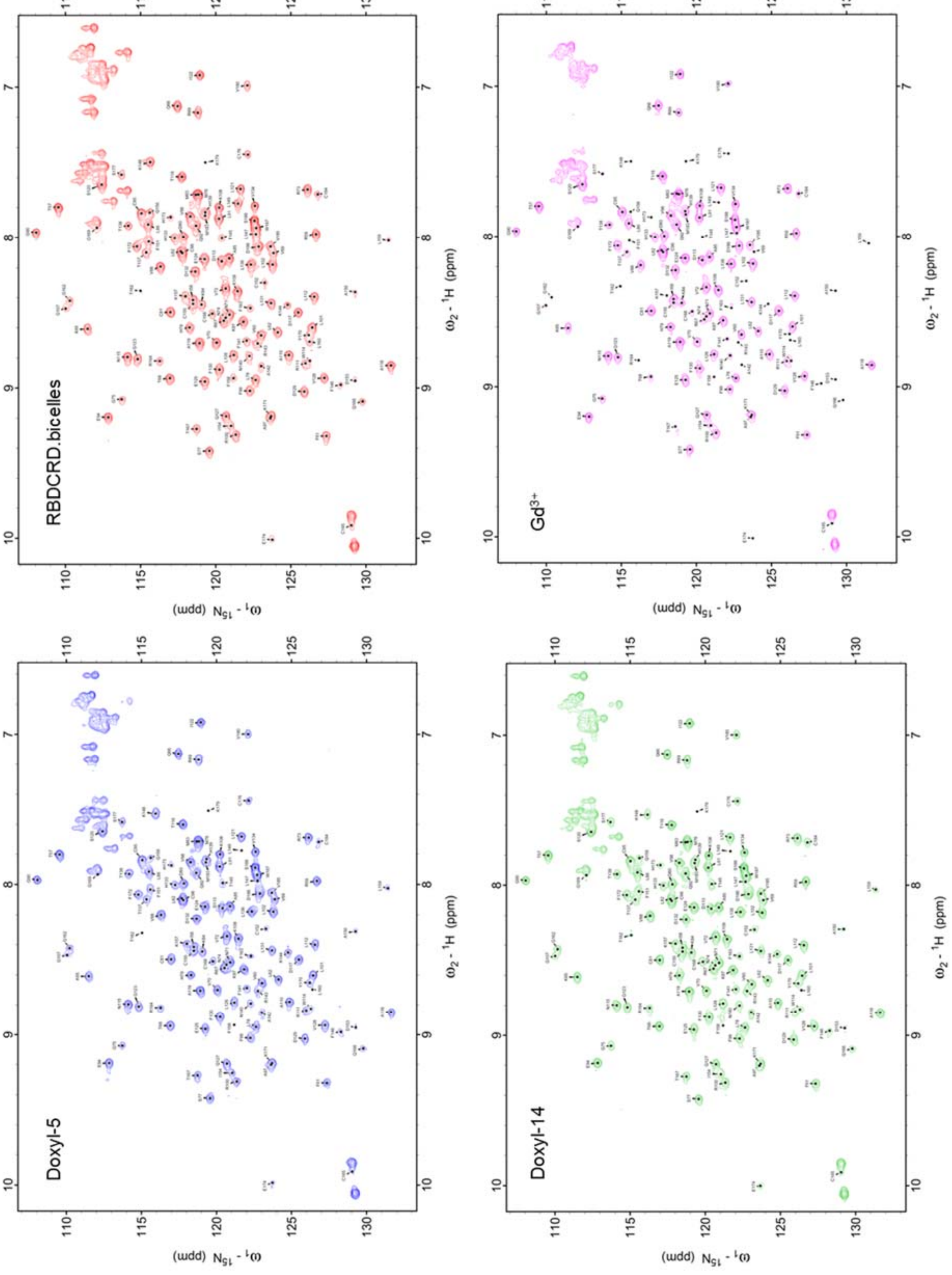

Figure S7. Spectra and assignments of free RBDCRD and RBDCRD bound to bicelles with doxyl-5, doxyl-14 and Gd^{3+}.

10. The methods for the PRE experiments are not fully described.

We thank the reviewer for the comment. The additional details have been provided in the methods (line 281-300) to describe the PRE experiments.

> "The PRE and control bicelles were prepared as followed: 50 mM DMPC/DMPS in chloroform stocks containing either Gd^{3+}, Doxyl-5 and Doxyl-14 at a molar ratio of (70:30, 69:30:1 and 69:30:1) were aliquoted into glass vials and dried under the gentle stream of argon in the hood. Dried lipids were re-solubilized with 100 mM DHPC dissolved in water to obtain a molar ratio of q=0.5. DLS was used to confirm the formation of bicelles (data not shown). For NMR measurements, 100 mM of RBDCRD in 20 mM HEPES, 100 mM NaCl pH 7.4 was flash frozen and lyophilized to remove all liquid. The lyophilized sample was then solubilized in 600 mL of preformed bicelles and rotated gently at 4 °C for 15 mins and then centrifuged to remove any precipitation. The samples were then placed in an NMR tube and HSQC data was collected on a Bruker 700 MHz instrument at 25 °C with 64 scans. To determine changes in peak intensity, peak heights were determined using POKY [37] to obtain PRE ratios = I/I_0, where I_0 is peak height in the control experiment with no PRE tags. An overlay of all RBDCRD bound to bicelles samples along with normalized PRE ratios are shown in Figure S7. PRE ratios for doxyl-5 and doxyl-14 were normalized using 1.18 and 1.32, respectively. Highly overlapped peaks (74, 76, 82, 83, 96, 97, and 171) and peaks with very low intensities (114, 179, and 182) were excluded from PRE analysis."

11. The CRD interaction residues and mutants listed in the manuscript closely resemble those already reported in a previously published work (DOI: https://doi.org/10.1038/s41467-021-21422-x). The authors should highlight any novel insights gained from the additional experiments or analyses presented in this study. Furthermore, while the manuscript discusses the role of membrane composition in regulating RAS-RAF interactions and nanocluster formation, it could benefit from a deeper comparison and integration of its results with the recent publication's insights regarding the selective interaction of RAF1(CRD) with phosphatidylserine (PS) and the detailed mapping of CRD residues involved in membrane interactions.

We thank the reviewer for this suggestion. The team is currently preparing a separate manuscript that is more focused on the specific protein-lipid interactions (which themselves required detailed study). We felt that it would be more appropriate to publish these studies separately as each converged to mostly parallel though connected results and narrative – thus attempting to combine them both would produce a large, complex, and difficult to follow publication. However, a brief discussion on comparison between

the suggested paper and our results has been added to the discussion section (line 893-908).

“In the pursuit of understanding the molecular details of RAS activation of RAF, Tran *et al.* recently solved the first crystal structure of GTPase domain of KRAS(1-169) and RAF1RBDCRD (52-188) ternary complex and showed that RBDCRD binds to KRAS as a one structural entity linked together by a short linker, and not as distinct domains [5]. In binding studies, the isolated CRD as a standalone domain does not exhibit binding to KRAS proteins. However, when the CRD coexists with the RBD, forming the RBDCRD domain, there is a notable increase in its binding affinity to KRAS. We also observe similar effects in our single particle tracking imaging experiments. CRD on its own has minimal effect on the diffusion of KRAS, while the tandem RBDCRD domain dramatically alters KRAS diffusion on the membrane. They also aligned their high-resolution crystal structure of KRAS-RBDCRD onto previously published MD simulations of KRAS on an anionic lipid bilayer composed of POPC and 30% POPS in order to predict membrane interacting CRD residues. Based on their model, they observed that few key residues such as K144 and L160 in the hydrophobic loop of CRD insert into the membrane, in agreement with NMR studies of CRD only on nanodiscs[24].”

12. The clustering methodology described appears to be a simplified approach to identifying protein clusters in comparison to more advanced clustering algorithms such as density-based or machine learning approaches. The methodology employed involves selecting a protein point and then assigning neighboring points within a specified distance to the same cluster label. While simplicity may be required/advantageous in complex biological systems, it is unclear whether the methodology provides a robust statistical or analytical basis. For example, it is unclear how additional factors such as density, distribution, or the actual nature of protein-protein interactions influence formation of clusters.

We thank the reviewer for the comment. The data extracted from our simulations are a series of 300 pairs of x- and y-coordinates that denote the positions of our proteins. Within the simulation, positions and co-localization of the proteins are driven solely by their combined interactions with the membrane. In terms of clustering methodology, there are certainly many to choose from. We felt that the most important factor to consider is *what* you are trying to capture. In our case, the analysis that we carried out had the single objective of determining the distribution of cluster sizes, and which protein types were within each cluster. Our defined metric of a ‘cluster’ (or proteins being co-localized with each other) used within the context of the paper was based on a distance cut-off from another protein, thus a simple spatial cut-off percolation method was chosen. We agree that some advanced clustering methodologies, such as K-means or DBSCAN, could yield some more subtle correlations, however the results would likely be contingent on the input search parameters.

13. The authors performed the conversion of coarse-grained (CG) frames to all-atom (AA) and executed AA simulations using CHARMM36 ff. However, specifics about the selection criteria for this conversion are lacking. The manuscript should provide clear details about the specific criteria, such as a) structurally stable frames, b) frames with diverse protein orientations with different CRD membrane insertions, and c) frames with significant protein-membrane interactions.

We thank the reviewer for bringing up this important point. The selection criteria for converting CG frames to AA was done through a dynamic-sampling approach employed in MuMMI. This sampling approach creates 3D histograms of the depth of CRD membrane insertion, and tilt/rotation angles describing the orientation of RAS G-domain. New samples were selected to satisfy a diversity factor of 20% by evaluating the 3D histogram with 1510 total bins. We provided additional details about the selection and performance of this sampling approach in our recent publication (https://pubs.acs.org/doi/full/10.1021/acs.jctc.2c01018#).

14. Given that the membrane composition can vary in different cellular contexts, some discussion on how changes in biologically-relevant lipid composition influence nanocluster formation and the membrane association/activity of the KRAS-RAF complex should be discussed.

We thank the reviewer for the comment. Additional discussion addressing the raised topics has been added to the manuscript (line 865-892) .

15. The MSD plots lack error bars. While the supplemental information contains a data table, the omission of errors on the figures limits the quantifiable assessment of diffusion differences between the wild type and mutant KRAS-RBDCRD complexes.

We thank the reviewer for the comment. The errors associated with each data point in the MSD plot are very small and nearly invisible in the plots. The raw data and the associated error bars for each MSD plots are tabulated in Table S2 -8 in the supplemental information.

16. The author's correlation between β-strand length and the depth of CRD loops in the membrane (Fig. 11a) raises concerns. The reported data appears to lack a significant difference (statistically) in β-strand lengths between various depths.

We thank the reviewer for the comment. We would like to point out that the error bars in Figure 10A corresponds to standard deviation. As we can observe in Figure 10 B, there are two main populations of total β-strand length (18 and 9), therefore standard deviations become large. We also generated a revised figure with error bars based on standard error, which result in extremely small error bars and demonstrate that there is

a statistically significant difference in β-strand lengths between various depths. In addition, Figure 10B clearly shows a population shift from long β-strand lengths (18) at low d_z values to short β-strand lengths (9) at high d_z values.

17. Fig. 11C illustrates potential orientations of the KRAS-RBDCRD complex at varying dz values. The authors should provide more detailed information about the interaction network and the criteria for identifying CRD orientation at distinct dz values.

We thank the reviewer for the comment. In previous work published by our team (Nguyen at al. https://doi.org/10.1016/j.bpj.2022.06.035), we identified and defined the 'CRD loops' and demonstrated that they insert the deepest into the membrane. Figures from that paper are shown below. There is also a two-component criterion (distance and torsion angle) to define the orientation in detail – however, it is difficult to project all the degrees of freedom into our analysis and as such the dz distance was chosen as the most informative metric.

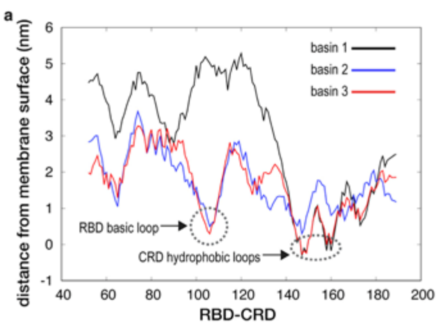

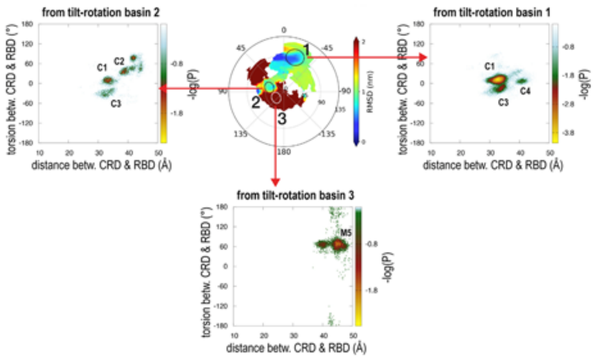

Figures from Nguyen at al. above – (L) the distances of all RBD CRD residues to the *surface* of the membrane (not the middle, like in our analysis) are shown. Highlighted are the CRD hydrophobic loops that we use to define our dz metric. (R) The basins of KRAS orientations can be correlated to the states and conformations of RBDCRD. Basins 1 and 3 have a very low dz – these low dz conformations show one major predominant state in the relative RBD-CRD orientations, indicating that the CRD becomes 'locked' into position when it engages the membrane. By contrast, basin 2 is at a higher dz where the CRD loops have likely not engaged the membrane and it is thus much more flexible.

18. In the discussion section, authors propose a model to describe the RAS-RAF interaction at the membrane, yet molecular models of membrane associated RAS-RAF complexes and pictorial representation(s) of the proposed model are lacking.

We thank the reviewer for the comment. A pictorial representation of our proposed model has been added to the main manuscript in Figure 11, Page 30

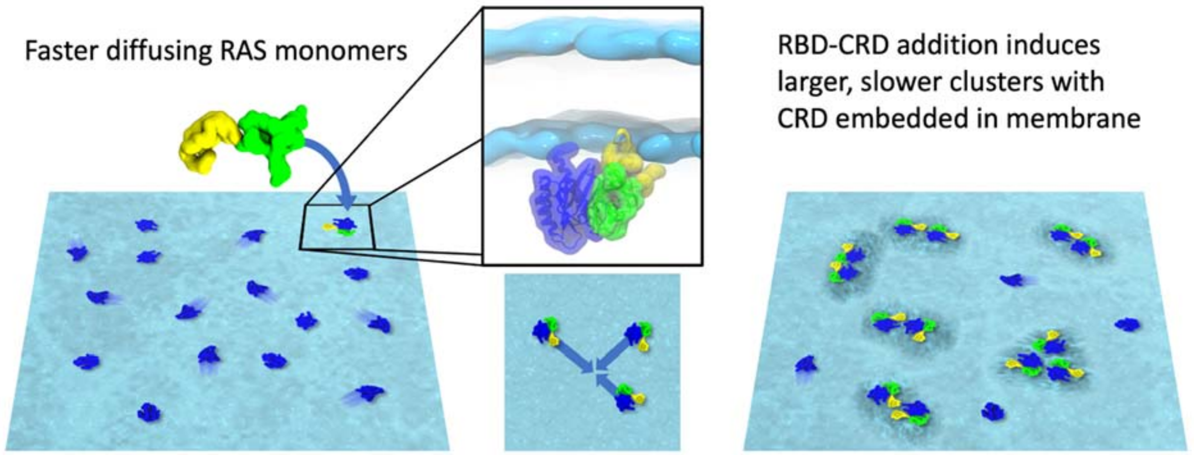

Figure 11. A pictorial representation depicting enhancement of nanoclusters upon RBDCRD binding to KRAS and the subsequent reorganization of lipid environment surrounding the clusters. The shadows around each RAS molecule in blue represents faster diffusion for monomeric KRAS. The absence of shadow around KRAS (blue) and RBDCRD (green-blue) complex infer slower diffusion for the complex. The grey shadow underneath KRAS-RBDCRD nanoclusters represents lipid reorganization.

19. The manuscript would benefit from a more detailed description that highlights novelty of the findings associated with 1) membrane composition playing a role in RAS/RAF diffusion mechanisms and clustering, 2) how simpler artificial membranes promote Brownian diffusion and more complex membranes exhibit a hierarchical diffusion process, and the 3) use of individual and combined RBD and CRD domains to tease out the RAF1 contributions to membrane association.

We thank the reviewer for the comment. Additional text discussing the following points have been added in the discussion section of the main manuscript.

Reviewer #4 (Remarks to the Author):

This manuscript describes results from an interesting piece of work combining single-molecule tracking, NMR, and computational analyses of KRAS-RAFRBDCRD interactions in supported and model membranes of complex lipid compositions. Consistent with previous reports by the same group and others, KRAS was found to diffuse at three different rates, suggesting multiple multimeric (and monomeric) forms. This is true for all lipid compositions tested, including binary PC/PS and PC/PIP2 membranes or more complex membranes made up of 6, 7 and 8 lipid species. In the most complex 8-component supported membrane, the dominant form (72%) diffuses at an intermediate rate whereas the fast-diffusing species represent only 11% of the total.

These distributions changed when RAF-RBDCRD is present, with the intermediate and slow diffusing species now being about equally populated (45 and 47%). This is interpreted as arising from the formation of KRAS-RBDCRD nanoclusters. Using NMR, the authors also show that a number of hydrophobic and basic residues of the CRD engage membrane lipids, which is supported by data from atomistic molecular dynamics (MD) simulations. CRD-membrane interaction is not a particularly new finding but still useful. Overall, this is an important contribution toward elucidating the organizing principles of RAS/effector complexes on membrane surfaces. The manuscript is well written and, for the most part, the conclusions are consistent with the data. However, there are some issues that require clarification.

1. Although not directly stated by the authors, it is remarkable that diffusion rates change little with lipid composition whereas the proportion of the fast, intermediate, and slow-diffusing species changes dramatically (Table S2). This is true irrespective of KRAS being alone or interacting with RBD or RBDCRD. It would seem, therefore, that clustering is an inherent property of KRAS molecules, with the different lipids helping tip the balance between populations. For example, in the 8-lipids system the fraction of the fast-diffusing species decreased by up to 10-fold whereas those of the slow and intermediate fractions increased by a similar amount. The other lipid mixtures have also altered the distributions albeit to a lesser extent. Would the authors comment on this and/or discuss their data in more detail?

We thank the reviewer for the comment. In our previous publication (https://doi.org/10.1016/j.isci.2021.103608), we show that the three-state diffusion of KRAS is achievable only on lipid bilayers with a complex lipid system. Furthermore, we compared KRAS diffusion on 3 different lipid mixtures, simple POPC/POPS, 8-lipid and 6-lipids without Sphingomyelin and Cholesterol. We designed the compositions of each lipid species in such a way that the overall electrostatics of each lipid mixture remained the same. Using atomic force microscopy, we show that 8-lipid bilayer undergoes phase separation while the 6-lipids and 2-lipids remains homogeneous on a mica surface. Therefore, in the publication we proposed that 8-lipid mixture undergoes a transient and mesoscale phase separation that leads to the three-state diffusion, favoring mostly the intermediate diffusion state. Here, we added two additional lipid compositions, POPC/PIP2 and a 7-lipid mix without cholesterol. Once again, the lipid compositions were designed to keep the overall electrostatics constant. We observe 3-state diffusion for not only the complex lipid mixtures but also the two lipid POPC/PIP2 mixtures, which has higher negative charge per lipid compared to PS lipids. Similarly, comparing the 6-, 7- and 8-lipid mixtures, the population of the fast state decreases and the population of the intermediate state increases systematically with an increase in the number of lipid species. Together, the results suggest that both electrostatics and heterogeneity in lipid species controls the lateral mobility of KRAS. We have added a paragraph discussing

the abovementioned points in the discussion section (Page 27, line 865-892) of the main manuscript.

2. Related to the above, it is clear from Fig 4 that the significance of the 8-lipid system lies in increasing confinement. This system differs from the 7-component system by the presence of cholesterol, suggesting that cholesterol is critical for confinement. The authors acknowledge this: "KRAS also randomly diffused in the cholesterol depleted 6-lipid and 7-lipid bilayers" but do not elaborate and explain. What does cholesterol do to confine KRAS? Would it make the bilayer rigid (33% of chol is significant)? How does this relate to the well-established fact that KRAS prefers cholesterol depleted, liquid disordered domains? Also, recent studies suggest that chol is mostly located in the outer leaflet of the plasma membrane, whereas KRAS is in the inner leaflet.

We thank the reviewer for the comment. We agree with the reviewers comment that inclusion of cholesterol in the 8-lipid mixture tends to increase the KRAS confinement on the lipid bilayer. Using atomic force microscopy studies, we have shown in our previous publication that the 8-lipid mixture when deposited onto a mica surface undergoes temperature dependent phase separation. As we mentioned in the added discussion, although we do not directly observe phase separation in TIRF experiments, it very likely that the lipid mixtures form transitory mesoscale domains. Regarding the last point raised by the reviewer, we acknowledge the recent lipodomics studies by Levental lab that shows cholesterol is mostly enriched in the outer leaflet of plasma membrane. However, our simple biomimetic system and the experimental technique used in this paper cannot mimic the real complexity of biological plasma membrane and cannot fully address specific KRAS-lipid interaction in live cells.

3) The authors state (and have shown previously; ref 17) that their 8-lipid system recapitulates diffusion rates measured in cells. Could this be fortuitous, because confined diffusion in cells is likely due to actin and other membrane components than cholesterol, at least for KRAS. It would be nice if the authors shed light on this issue. This is particularly important now that they show it is cholesterol, not just the complexity of the membrane, that plays a critical in yielding a more confined diffusion (per Fig 4).

We appreciate the reviewer's input. We agree with the idea that the restricted movement of molecules in cells may be influenced not only by cholesterol but also by other diverse components like actin and various membrane elements. This confinement could also be linked to the formation of nanoclusters or signaling complexes involving KRAS, such as its assembly with RAF and other effectors. Our previous work in eLife (https://doi.org/10.7554/eLife.47654) discusses the diffusion of KRAS and related isoforms in live cell membranes and how it changes with electrostatic variations in the hypervariable region (HVR). When comparing diffusion in artificial bilayers and cellular membranes, we find that KRAS moves much more slowly in cells, likely due to the

presence of actin filaments, other membrane proteins, and lipids, creating a more crowded, viscous, and heterogenous lipid environment. In our experiments with supported lipid bilayers, KRAS displays confined diffusion in an 8-lipid system (including cholesterol) but freely diffuses in simpler bilayers. However, confined diffusion occurs under specific conditions, such as with a POPC/PIP2 lipid bilayer or when treated with the crosslinker molecule DSSO, even in a simple POPC/POPS lipid bilayer. Additionally, when KRAS binds to RBDCRD, its diffusion becomes confined in all lipid mixtures. This indicates that KRAS's restricted movement is influenced by the heterogeneous membrane composition, including its charge and lipid makeup, as well as the oligomer state of KRAS. We have included a paragraph in the discussion section (line 865-892) to address the implications of different lipid compositions on KRAS diffusion.

4) The authors state “One hypothesis is that upon RBDCRD binding, KRAS-RBDRCRD complexes instantaneously assemble into clusters with slow diffusion” (page 15). As noted in 1 above, it would seem from the data that KRAS itself is able to instantaneously form clusters, so that RBDCRD binding decreases the overall diffusion rate by increasing the population of the slower species rather than driving cluster formation. Would the authors comment on this?

We appreciate the reviewer's observation. Indeed, on a complex 8-lipid bilayer, KRAS has the capability to rapidly form clusters. The reason behind the observed decrease in diffusion speed upon RBDCRD binding could be attributed to either an increase in the number of slower-moving KRAS species, as the reviewer suggested, or an augmentation in the formation of clusters, or potentially both mechanisms working in tandem. To deconvolute this, we also used a simple POPC/POPS lipid bilayer, where KRAS on its own clearly shows Brownian diffusion, implicating a monomer state. Upon the addition of RBDCRD to the system, KRAS exhibited a three-state diffusion, characterized by an increase in slow-moving components. Comparing the Mean Squared Displacement (MSD) plots of KRAS plus RBDCRD on a POPC/POPS lipid bilayer with those of KRAS treated with the dimer-forming BI2852 compound and the cluster-inducing crosslinker molecule DSSO, it was observed that KRAS plus RBDCRD displayed the slowest diffusion. This suggests that the binding of RBDCRD to KRAS induces clustering of the proteins, with this effect being more pronounced on a complex 8-lipid bilayer. We have added additional discussion of the topic in the discussion section (line 865-892) of the manuscript.

5) The significance of most of the simulations data is unclear (at least to this reviewer).

We thank the reviewer for this critique, which we address in line with the reviewer's major points below:

In particular, the data from continuum modeling (Fig 6) does not seem to add much to the discussion…

The continuum model data in that figure shows that we observe a decay in clustering size driven by the lipids. However, the key takeaway from Figure 6 is that the presence of RBDCRD increases the likelihood of clustering by ~20-fold. This complements several of the experimental findings.

and the diffusion rates from the CG simulations shown in Fig 7 are problematic in many respects. First, although understandable from a technical standpoint, the average diffusion rates from the simulations are about 10-fold larger than that experimentally measured for the dominant species (the extrapolation in Fig 8 shows that the simulation failed to sample larger clusters).

With regards to comparing the diffusion rates, the closest and most reliable comparison is the KRAS monomer vs the fastest-diffusing species from the experiments (the systems are not convoluted by the presence of additional RBDCRD domains, uncertainties in higher order clusters and compositions). These values showed good agreement with each other (both ~4 um^2/s). However, we do agree that a large proportion of the diffusion analysis may be over-emphasized due to the inherently noisy diffusion calculations that come from the simulations. Thus, we have removed this section and placed truncated version in the supplemental information.

Secondly, it is not clear what fraction of molecules in the simulation box or for what fraction of time have "clustered". The numbers quoted for KRAS-only 4-mers "~30, compared with >3000" suggest a very small (<<1%) fraction of clusters compared to total. This is concerning, and raises the question of whether or not true cluster formation has occurred as compared to random clashes.

The CG simulations are derived/selected from the continuum model, in which the colocalization of the protein is only driven by lipid interactions and the reorganization of the lipids around the proteins – thus we do not expect clustering to the same level as seen in the experiments.

Thirdly, it appears that the 1-mer, 2-mer and 3-mer KRAS-RBDRDs diffuse with comparable rates and slightly faster than the 4-mer, 5-mer and 6-mer clusters that diffuse at a comparable rate (Fig 7B). This is confusing. Is there any reason for the existence of these two classes despite their different sizes? The same could be said about Fig 7A.

The following was written to address a similar point by one of the other reviewers, which we have amended here:

Diffusion calculations of membrane proteins are inherently difficult – especially for complex, multi-domain systems. There are several, varied reasons for this, such as:

i) Diffusion calculations from simulation data are often extremely noisy (as seen in the violin plots of Figure 7) and can be very **system-dependent** (indeed, being able to obtain data from the KRAS simulations that is even close to fitting the expected decay trends is quite a challenge – the fact that we were able to actually do so is likely a result of the extensive simulation campaign that was carried out, ~35,000!)
ii) There is much less sampling of these KRAS-RBDCRD systems
iii) Longer relaxation times are needed for a big cluster – further compounding the issues in points (i and ii)
iv) The precise metric of how to measure diffusion is complicated – often the center of mass of the protein is chosen and the movement of that coordinate is tracked. However, with so many domains and proteins all in motion, any diffusion measurement become *even more* noisy and muddled. Thus, the increased internal dynamics of the larger clusters (i.e. one KRAS-RBDCRD moving around while still being bound to the others) may likely inflate the diffusion value.

To summarize the above points, the following sections of text have been:

> "Despite diffusion calculations from simulation data often being extremely noisy and variable…" (line 660)
>
> "KRAS-RBDCRD diffusion did not allow for such fitting – likely due to both insufficient sampling of the simulations and additional complexities associated with diffusion calculation of the more intricate, multi-domain systems." (line 666-669)

REVIEWERS' COMMENTS:

Reviewer #1 (Remarks to the Author):

The authors have revised the manuscript and resolved most of my concerns, except for the last point. The revised discussion for Fig 8C is almost the same as the original, in which the concepts of dz and depths are confused.

Reviewer #4 (Remarks to the Author):

The authors have adequately addressed my previous concerns. I don't have any more suggestions.

REVIEWERS' COMMENTS:

Reviewer #1 (Remarks to the Author):

The authors have revised the manuscript and resolved most of my concerns, except for the last point. The revised discussion for Fig 8C is almost the same as the original, in which the concepts of dz and depths are confused.

We thank the reviewer for helping us to clarify and clear up this admittedly confusing issue. We relabeled the inset legend in Fig. 8c and have included the correct description of dz and various values of dz in a revised explanation for Fig. 8c in the manuscript (line 419-427):

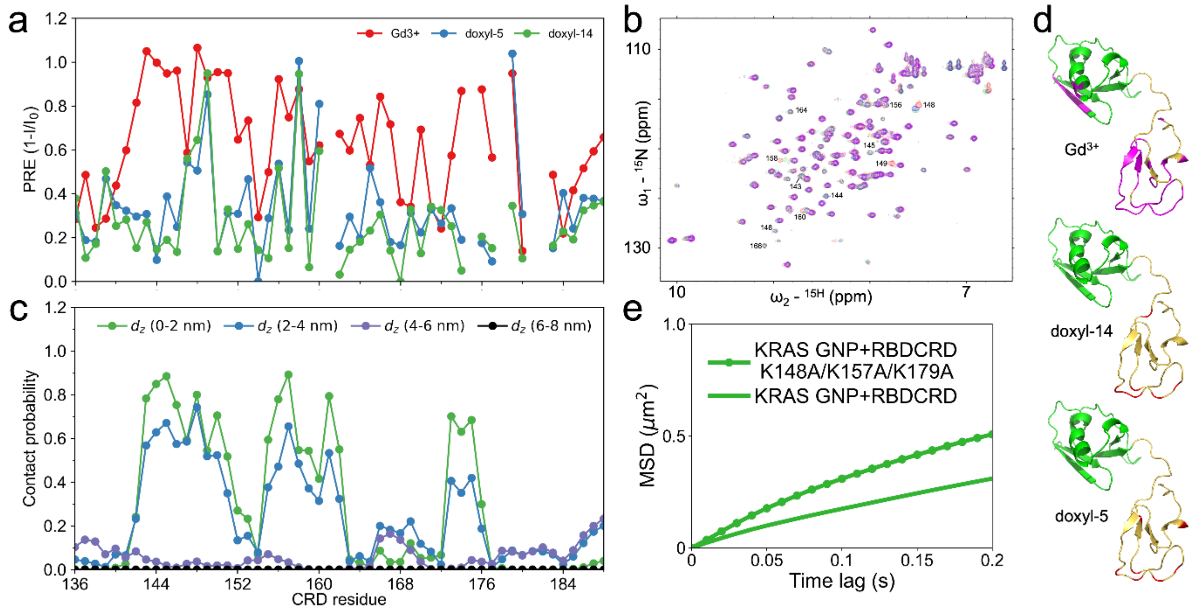

Figure 8. Mechanistic insights into RBDCRD-membrane interactions. (a) Dot plot representation of NMR-PRE ratios (I/I0) plotted as (1-(I/I0) to compare with contact probabilities obtained from the MuMMI AA simulations. (b) HSQC spectra showing reduction in intensities of CRD residues of RBDCRD bound to DMPC:DMPS bicelles (red) upon the addition of doxyl-5 (blue), doxyl-14 (green) and Gd^{3+} (magenta). (c) Contact probability distribution of CRD residues in KRAS-RBDCRD as a function of the distance between the COM of hydrophobic/cationic CRD loop residues and the COM of the bilayer along the bilayer normal (dz), obtained from MuMMI AA simulations. (d) CRD residues with PRE ratios of ≤50% are mapped onto a model of RBDCRD: doxyl-5 and doxyl-14 (red) and Gd^{3+} (magenta). (e) Mean square displacement plots of KRAS on 8-lipid bilayer when bound to wild type RBDCRD (solid) and mutated RBDCRD K148A/K157A/K179A (dotted).

"Fig. 8c shows the contact probability calculated for each CRD residue at different distances between the COM of hydrophobic/cationic CRD loop residues and the COM of the bilayer along the bilayer normal (d_z) (see Fig. 9c for a visual representation of d_z). The d_z of 0-2 nm spans from the center of the lipid bilayer to cover almost all of the lipid tails and aligns with the NMR PRE experiments performed with doxyl-5 lipids and doxyl-14 lipids. The d_z of 2-4 nm includes the outermost layer of the lipid headgroups and a small part of the lipid tails which mostly aligns with NMR experiments with gadolinium spin label on the lipid headgroup. Lastly, the d_z of 4-6 nm and 6-8 nm represents RBDCRD structures that are not in contact with the lipid bilayer".

Reviewer #4 (Remarks to the Author):

The authors have adequately addressed my previous concerns. I don't have any more suggestions.

We appreciate and thank the reviewer for their insightful comments and suggestions in the review process of this paper.
